# Platinum(II) Phenylpyridyl Schiff Base Complexes as Latent, Photoactivated, Alkene Hydrosilylation Catalysts

**DOI:** 10.1021/acscatal.4c01353

**Published:** 2024-04-30

**Authors:** Helena G. Lancaster, Joe C. Goodall, Samuel P. Douglas, Laura J. Ashfield, Simon B. Duckett, Robin N. Perutz, Andrew S. Weller

**Affiliations:** † Department of Chemistry, University of York, Heslington, York YO10 5DD, U.K.; ‡ Johnson Matthey Technology Center, Blounts Court Road, Sonning Common, Reading RG4 9NH, U.K.

**Keywords:** platinum, hydrosilylation, photolysis, photoactivation, kinetics, mechanism

## Abstract

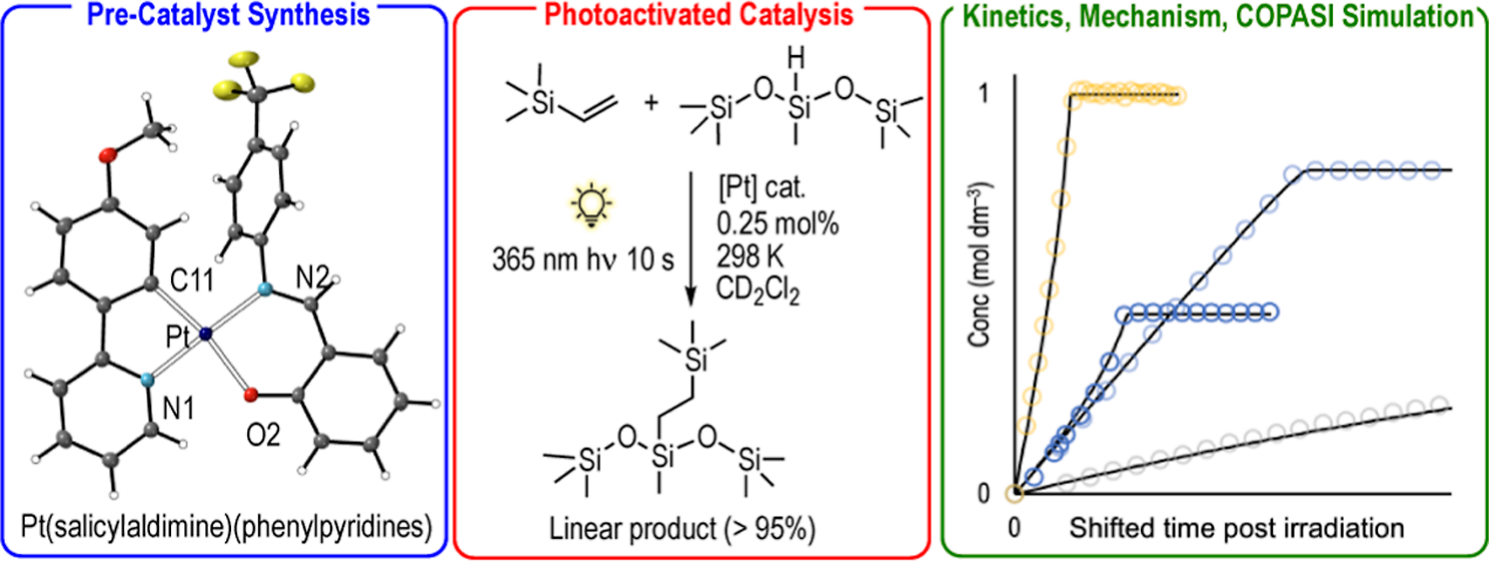

Photoactivated catalysts for the hydrosilylation of alkenes with silanes offer temporal control in manufacturing processes that require silicone curing. We report the development of a range of air-stable Pt(II) (salicylaldimine)(phenylpyridyl), [Pt(sal)(ppy)], complexes as photoinitiated hydrosilylation catalysts. Some of these catalysts show appreciable latency in thermal catalysis and can also be rapidly (10 s) activated by a LED UV-light source (365 nm), to give systems that selectively couple trimethylvinylsilane and hexamethylsiloxymethylsilane to give the linear hydrosilylation product. Although an undetectable (by NMR spectroscopy) amount of precatalyst is converted to the active form under UV-irradiation in the timescale required to initiate hydrosilylation, clean and reliable kinetics can be measured for these systems that allow for a detailed mechanism to be developed for Pt(sal)(ppy)-based photoactivated hydrosilylation. The suggested mechanism is shown to have close parallels with, but also subtle differences from, those previously proposed for thermally-activated Karstedt-type Pt(0) systems.

## 1 Introduction

**Scheme 1:**
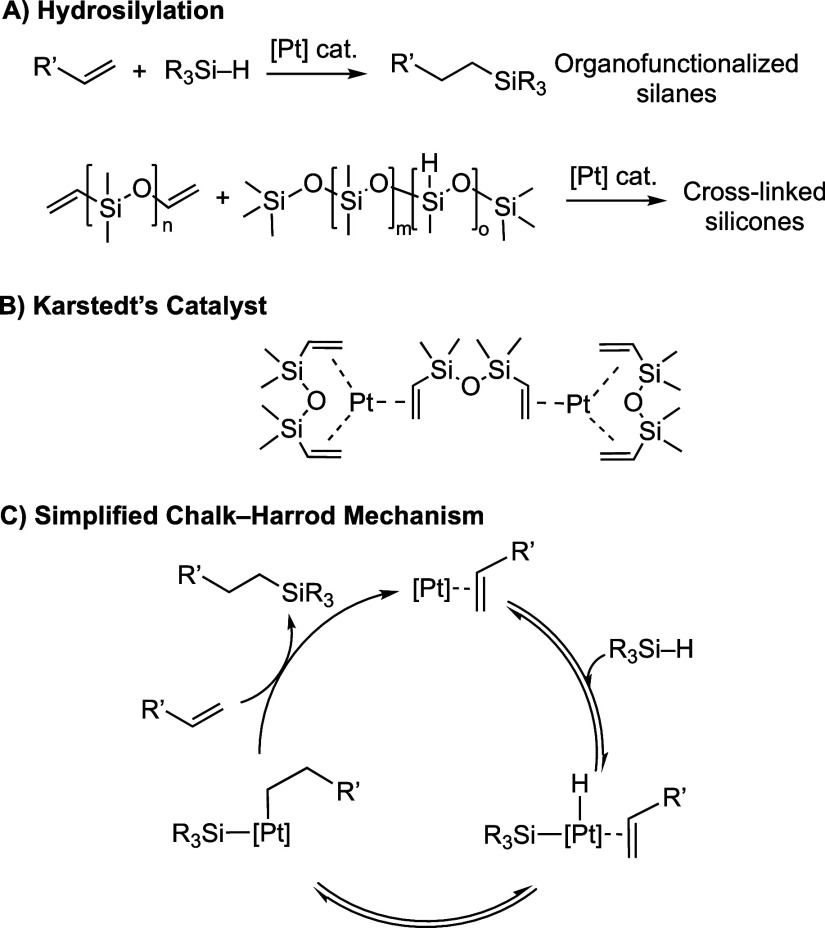
Hydrosilylation, Karstedt’s Catalyst, and a Simplified Chalk-Harrod Mechanism

Catalytic hydrosilylation, the addition of a Si–H bond over an alkene, alkyne, or carbonyl group, is an important reaction in industrial, fine-chemicals, and materials chemistry.^1−5^ Hydrosilylation is widely employed for the synthesis of organofunctionalized silanes and silicone polymers that find applications as sealing materials and adhesives. Silicone-curing applications are particularly important as cross-linking between multifunctional Si–H and vinyl polymers leads to three-dimensional networks that can be used as silicone elastomers or release coatings (Scheme 1A).^2^ While many catalyst systems have been reported,^1^ the most commonly used are based on Pt(0) systems, and in particular Karstedt’s catalyst,^2,6^ Pt_2_(dvtms)_3_ (dvtms = 1,3-divinyl-1,1,3,3-tetramethyldisiloxane) or derivatives thereof^7,8^ (Scheme 1B). Despite the considerable maturity of the field, such Pt(0)/alkene catalysts have remained the benchmark for hydrosilylation, given the very low loadings used and high activities they present.^2,4,9,10^ Hydrosilylation by such catalysts is broadly accepted to proceed in a homogeneous regime via the Chalk-Harrod mechanism (Scheme 1C),^11,12^ or closely related variants.^9,10,13^ Karstedt’s catalyst has attracted significant mechanistic scrutiny,^9,10,13^ and the active catalyst is likely based upon Pt(alkene)_3_-type species. However, the role of nanoparticles, that can be formed under catalytic conditions, adds further complexity,^1,10,14−17^ especially as changes in speciation may be dynamic with regard to reaction progress.^18^

**Scheme 2:**
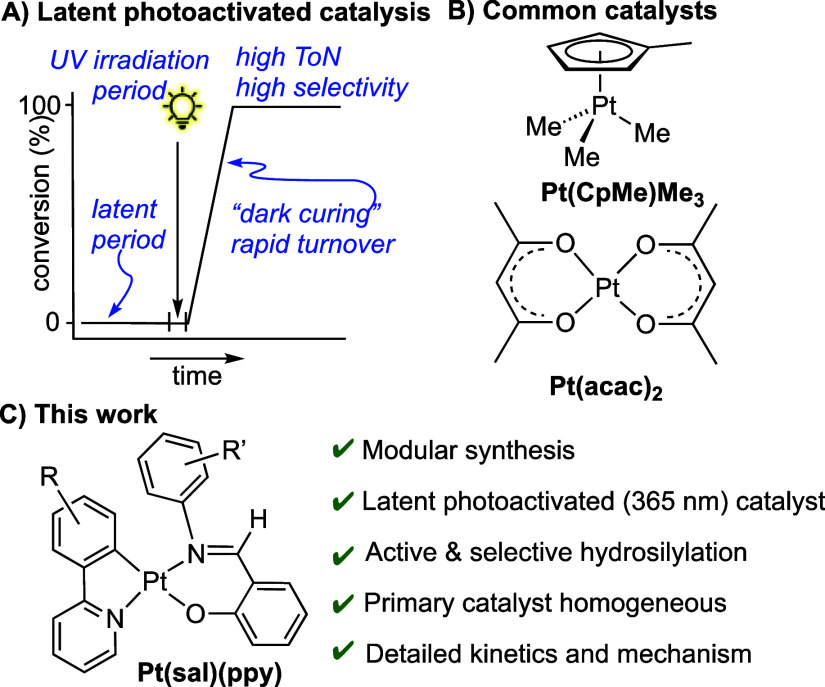
(A) Idealized Dark-Curing Time/Conversion Plot in Which Hydrosilylation Is Triggered by Time-Gated UV-Irradiation. (B) Common Photoactivated Hydrosilylation Catalysts. (C) This Work

Photoactivated catalysts for the hydrosilylation of multifunctional Si–H and vinyl polymers to give cross-linked silicones are of particular interest as they give temporal control as well as the potential for increased shelf life of premixed catalyst/substrate mixtures.^19−26^ Some important applications of such catalysts come from the production of fast-curing films and in additive or continuous manufacturing processes.^19^ Ideally such systems combine high precatalyst stability in the silane/vinyl polymer mixture (latency) with rapid photoactivation, to afford an active catalyst that works at low loadings with fast rates of turnover. In addition, so-called “dark-curing”^19^ is particularly advantageous as cross-linking can be triggered by a short burst of UV-irradiation that is followed by catalysis (Scheme 2A). The best studied photoactivated catalysts, both in academic journals and the patent literature,^19^ are those based upon Pt(CpMe)Me_3_ (CpMe = η^5^-C_5_H_4_Me)^21,23^ and Pt(II)(β-diketonates), exemplified by Pt(acac)_2_^22,24,27−29^ (Scheme 2B). The former catalysts are acutely toxic and volatile but more reactive than the latter under photohydrosilylation conditions,^30^ while the latter suffer from poor solubility in siloxane substrates.^29^ As for thermally activated catalysts, there has been considerable activity devoted to understanding the mechanisms of action of these systems. Pt(CpR)Me_3_ type-catalysts are believed to operate via the formation of Pt(0) nanoparticles,^23^ while Pt(acac)_2_ is suggested to operate via a fast homogeneous catalyst, alongside a slower heterogeneous one.^22^ While there have been reports in the open literature of their uses as dark-curing catalysts, these studies are often run under very different experimental conditions, and there is relatively limited attention paid to any latency periods prior to UV-irradiation or indeed a detailed kinetic analysis of subsequent catalysis.^22,29,31,32^ Other photocatalysts, or photoactivated catalysts, for hydrosilylation have been reported.^25,26,33−40^

In this contribution, we report on the use of Pt(salicylaldimine)(phenylpyridyl), Pt(sal)(ppy), complexes as photoinitiated hydrosilylation catalysts (Scheme 2C), which are related to Pt(acac)_2_ in that they are Pt(II) catalysts with LX-ligands. The ability to vary the ligand-set in these complexes in a modular way, in contrast to homoleptic Pt(acac)_2_, allows for catalysts to be developed that show latency toward thermal hydrosilylation, that can be rapidly (10 s) activated by a simple LED UV-light source (365 nm). Despite an undetectable (NMR spectroscopy) amount of precatalyst being converted to the active form under UV-activation, clean and reliable kinetics can be measured for these systems that allow for a detailed mechanism to be developed for Pt-based photoactivated hydrosilylation, aided by simulation (COPASI^41^). The suggested mechanism is shown to have close parallels with, but also subtle differences from, those proposed for thermally activated Karstedt-type systems.

## 2 Results and Discussion

### 2.1 Synthesis of a Modular Catalyst System, Pt(sal)(ppy)

Cyclometalated complexes based around Pt(salicylaldimine)(phenylpyridyl), Pt(sal)(ppy), have well established photophysical properties (e.g., they display aggregation-induced photoluminescence),^42,43^ are considered to have low cytotoxicity,^43^ offer potential for hemilabile reactivity profiles through decoordination of the imine-group,^44^ and have modular synthetic routes from readily accessible ligands.^42,43,45^ While such complexes, to our knowledge, have not been used in hydrosilylation catalysis, they are structurally related to Pt(acac)_2_-photoinitiated catalysts (Scheme 2B). Related complexes such as Pt(ppy)Cl(PPh_3_) have been used for thermal hydrosilylation at 100 °C.^46^

**Scheme 3:**
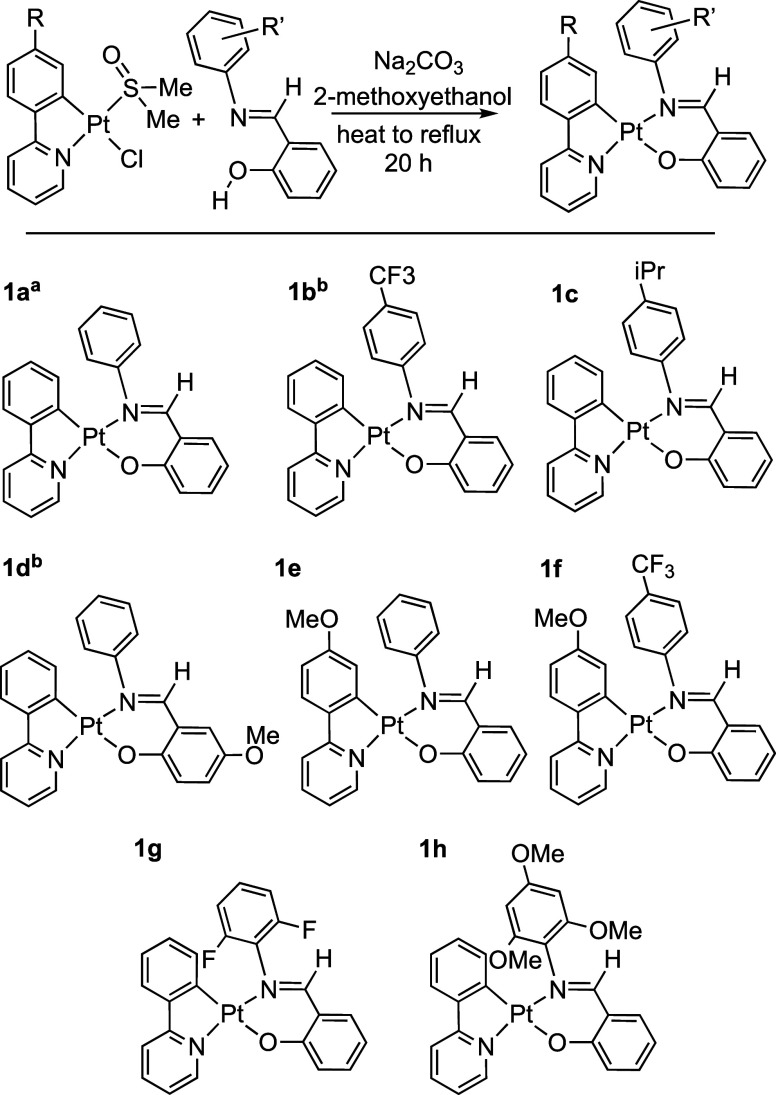
Synthesis of Pt(sal)(ppy) Complexes. ^a^Ref (42). ^b^Ref (43)

Starting from a solution of the Pt(ppy)(dmso)Cl precursor in 2-methoxyethanol,^47,48^ the appropriate proto-ligand was added, and the solution was heated to reflux overnight with Na_2_CO_3_. The target complexes **1a** to **1h** (Scheme 3) were isolated in moderate (40–50%) yield as orange powders following removal of solvent, precipitation with hexane, and recrystallization. The new complexes have been characterized by solution NMR/UV–visible spectroscopies/elemental analysis/electrospray ionization mass spectrometry, and the data are in full accord with the proposed structures, some of which have been previously synthesized.^42,43^ Recrystallization from CH_2_Cl_2_/hexane provided material suitable for single crystal X-ray diffraction for selected examples. These complexes are all air-stable (at least 12 months) and require no special conditions for storage.

**Figure 1:**
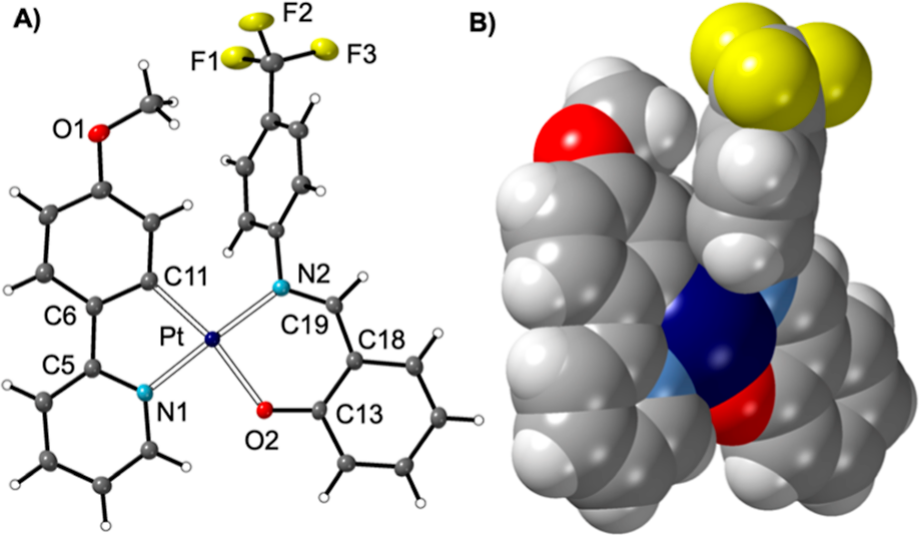
Molecular structure of complex **1f**. (A) Displacement ellipsoids are shown at the 50% probability level. Selected bond lengths (Å) and angles (deg): Pt–C11, 2.008(3); Pt–N1, 2.017(2); Pt–O2, 2.0655(19); Pt–N2, 2.028(2); C11–Pt–N1, 81.03(10); N1–Pt–O2, 88.01(8); O2–Pt–N2, 90.44(8); N2–Pt–C11, 100.90(10). Angle between planes from C11–C6–C5–N1–Pt/Pt–N2–C19–C18–C13–O2 = 8.58(9)°. (B) Space filling representation (van der Waals radii).

Using the new complex **1f** as a representative example, the ^1^H NMR spectrum (CD_2_Cl_2_) presents distinctive resonances in the aromatic region that show ^195^Pt–H coupling, which are assigned to the proton ortho to nitrogen on the phenylpyridyl ligand and the imine-proton [δ 9.42, *J*(PtH) = 33 Hz; δ 8.25, *J*(PtH) = 71 Hz], respectively (Figure S16). In the UV–vis spectrum (CH_2_Cl_2_), intense bands below 270 nm are assigned to π–π* ligand-centered transitions, while lower-energy transitions between 350 and 450 nm are assigned to metal-to-ligand charge transfer (Figure S28), as informed by previous studies.^43,45^ The solid-state structure of **1f** shows a pseudo-square-planar Pt(II) center (Figure 1), with the ligands twisted away from each other due to a steric clash between the N–Ar group and the phenyl–pyridine ligand [angle between Pt(ppy) and Pt(sal) ligand planes = 8.58(9)°].

### 2.2 Catalyst Scoping for Latency and Activity: Optimization of NMR Acquisition Parameters, and Thermal Activation in the Absence of UV-Light

**Scheme 4:**
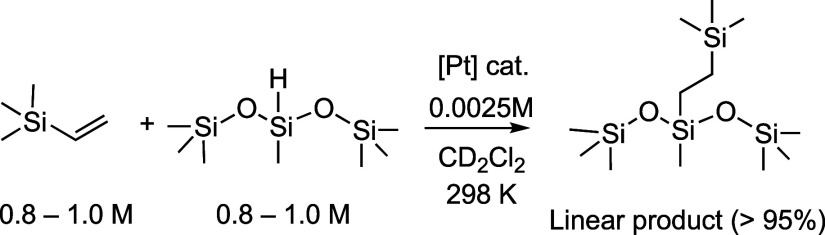
Hydrosilylation Substrates and Conditions for Thermally Activated Catalysts

Initial studies used catalyst systems **1a–h** under thermal conditions, i.e., in the dark at ambient temperature. Experiments were performed in dried and degassed^22^ CD_2_Cl_2_, in an inert atmosphere in J. Young’s NMR tubes, using 0.0025 M precatalyst solutions (i.e., 0.25–0.30 mol %) and 0.8–1 M solutions of dried (CaH_2_) and vacuum distilled trimethylvinylsilane/hexamethylsiloxymethylsilane with an internal standard of mesitylene (Scheme 4). These substrates were chosen to avoid the complications of alkene isomerization^10^ and to reflect commercially relevant substrates.^1,19^ Detailed optimization of the NMR acquisition parameters resulted in the need for a long relaxation delay of 45 s to ensure quantitative integration of the Si–H, alkene, and methylene groups for the NMR analysis of reaction mixtures.^49^ Solutions containing catalyst/substrate mixtures were kept in the dark before being inserted into the NMR spectrometer.

**Figure 2:**
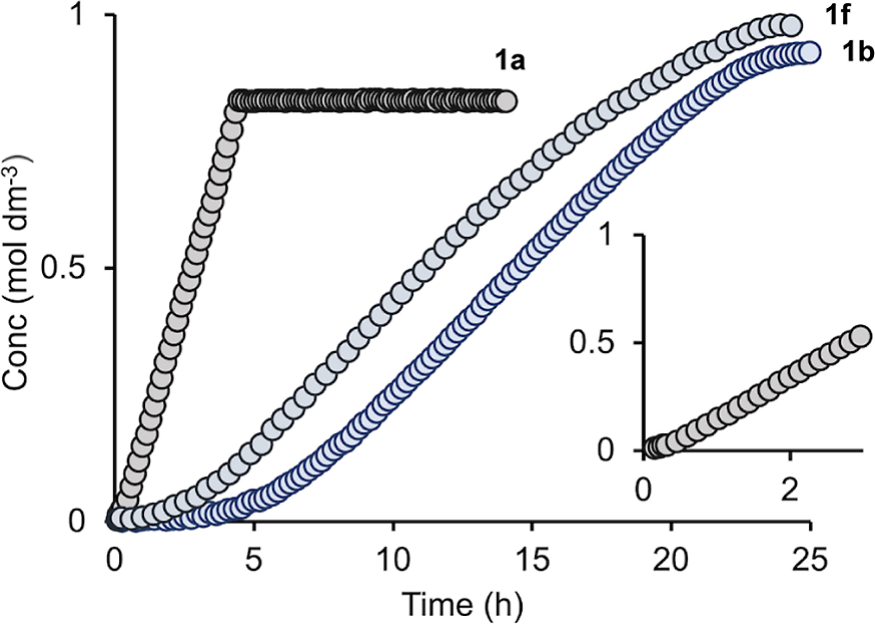
Concentration of linear product versus time for thermally activated catalysis, using **1a**, **1b**, and **1f** precatalysts (298 K, 0.0025 M, CD_2_Cl_2_). Inset shows an expansion for **1a**, revealing the induction period.

All of the catalysts **1a**–**h** promote complete turnover for hydrosilylation, with greater than 95% selectivity for the linear (anti-Markovnikov) product. Consideration of the temporal profiles, however, shows very different rates of reaction and reaction profiles, Table 1 and Figure 2. A sigmoid profile, with an associated induction period^50^ and a linear pseudo-zero-order regime of maximum rate of turnover, is observed for many of the precatalysts (**1b**, **1e**, **1f**, and **1g**). However, other precatalysts (**1a**, **1c**, **1d**, and **1h**) show much shorter induction periods and apparent zero-order reaction profiles that operate over nearly all of the reaction profile. Three representative examples are given in Figure 2. The time measured to completion varies from 1.7 (**1c**) to 25 h (**1b**). While induction periods and sigmoid profiles can signal the formation of a colloidal catalyst,^51−53^ they can also be consistent with a process that turns a homogeneous precatalyst to an active homogeneous catalyst. There is no reaction in the absence of a catalyst.

**Table 1:** Thermally Activated Hydrosilylation of Trimethylvinylsilane and Hexamethylsiloxymethylsilane^a^

entry	[Pt]-catalyst	time/h (TOF_app_/×10^–5^ s^–1^)^b^	induction period^c^/h
1	**1a**	4.5 (5.51 ± 0.06)	0.16
2	**1b**	25.0 (1.47 ± 0.02)	2.80
3	**1c**	1.7 (15.3 ± 0.09)	0.13
4	**1d**	8.0 (3.40 ± 0.03)	0.21
5	**1e**	11.0 (3.8 ± 0.1)	0.35
6	**1f**	24.3 (1.61 ± 0.04)	1.40
7	**1g**	7.2 (8.1 ± 0.2)	0.70^d^
8	**1h**	5.6 (5.12 ± 0.03)	0.45

a Conditions: 0.8–1.0 M substrates, CD_2_Cl_2_, [cat.] = 0.0025 M, 298 K.

b TOF_app_ = Apparent^54^ maximum turnover frequency as measured from the pseudo-zero-order region of the reaction profile.

c Induction period is estimated as the time taken for 1% conversion to occur.

d The method of Morris was used to estimate the induction period.^55^

Post catalysis solutions appear homogeneous, with no observable change of the original orange color of the precatalyst. In the corresponding ^1^H NMR spectra, the distinctive signals due to the precatalysts [that show *J*(PtH) coupling] are still observed at the end of catalysis. These signals integrate to demonstrate that the precatalyst is essentially unchanged in concentration to the detection limit of this low intensity (0.25 mol %) signal in the ^1^H NMR spectrum, ∼5%. No signals are observed in the ^1^H NMR spectrum that would indicate either a new species or decomposition. Thus, we suggest that only a very small amount (less than 5%) of the precatalyst is activated, meaning that the TOF_app_^54^ determined represent lower bounds for catalyst activity. Catalyst **1c** (^i^Pr substituted) presents the most active thermal catalyst, while catalysts **1b** and **1f** are the slowest (CF_3_ substituted) but also show the longest induction periods. Thus, in terms of our stated objective, to develop controllable, latent, photoactivated, hydrosilylation catalysts, complexes **1b** and **1f** show the most promise as, although they do not promote the fastest turnover under thermal activation, they do show good latency.

### 2.3 Thermally Activated Catalyst **1b**: Heterogeneous or Homogeneous?

Distinguishing between heterogeneous and homogeneous catalysis in alkene hydrosilylation has been a long-standing issue.^14,16,18,22,23,29^ Commonly used methods^56^ are the Hg-drop test^57^ and addition of the, tub-shaped, dialkene DBCOT (dibenzocyclooctatetraene).^58^ The former is known to suppress colloidal catalysis, while the latter is a selective poison for homogeneous, molecular, species. Using catalyst **1b** as an exemplar, addition of excess Hg after productive catalysis was well under way (∼40% conversion and vigorous shaking of the NMR tube^59^) did not result in a significant attenuation of activity (Figure S40). Addition of 0.3 equiv of DBCOT at a similar stage of conversion had a significant decelerating effect but did not completely inhibit turnover (Figure S41). We interpret these observations as signaling that the active catalyst for this thermally activated hydrosilylation is homogeneous. Since the Hg drop test was negative, we explain the partial suppression by DBCOT by reversible exchange of diene with the small amount (less than 5%) of thermally generated active catalyst, so that it only strongly attenuates, rather than halts, catalysis. Exchange of DBCOT in Pt-complexes has been reported^13^ as has partial suppression by DBCOT in Pt-catalyzed hydrosilylation.^22^

### 2.4 Photoactivation of Precatalyst **1b**. Significant Rate Acceleration on UV-Activation and Recharging Experiments

All of the precatalysts, **1a**–**1h**, have been evaluated as photoactivated catalysts (Supporting Information). While all show significant rate enhancements on brief irradiation with UV light (10 s), **1b** and **1f** combine thermal latency with photoactivity. These two precatalysts **1b** and **1f** were thus taken forward for studies in photoactivated hydrosilylation. Studies with **1b** are reported in detail here (see Supporting Information for **1f**).

The same catalyst and substrate concentrations were used as for the thermal studies, and catalysis was performed in a standard 5 mm J. Young’s NMR tube. The latency period was confirmed by acquiring data for ∼1.5 h at 298 K, during which time no turnover was observed. The NMR tube was then removed from the spectrometer and placed in a bespoke UV reactor that has four, power-controlled, 365 nm LED lamps each with a 1.36 W radiant output (see the Experimental Section). Cooled compressed air was purged through the reactor housing. A thermocouple located close to the NMR tube showed that there was only a minimal increase in temperature during photolysis (maximum of 2 °C rise over 160 s). This isolates any observed changes in activity to the photochemical activation process. The NMR tube was irradiated for a predetermined time that was controlled by a timer: 10, 60, and 120 s. The NMR tube was then returned to the spectrometer and data acquisition restarted after shimming, in the dark. As the molar absorption coefficient of **1b** at 365 nm is ∼10^4^ dm^3^ mol^–1^ cm^–1^ (Table S3), the penetration of the radiation is much less than the internal radius of the NMR tube, i.e., the precatalysts should be considered to be optically dense under our conditions.^60^

**Figure 3:**
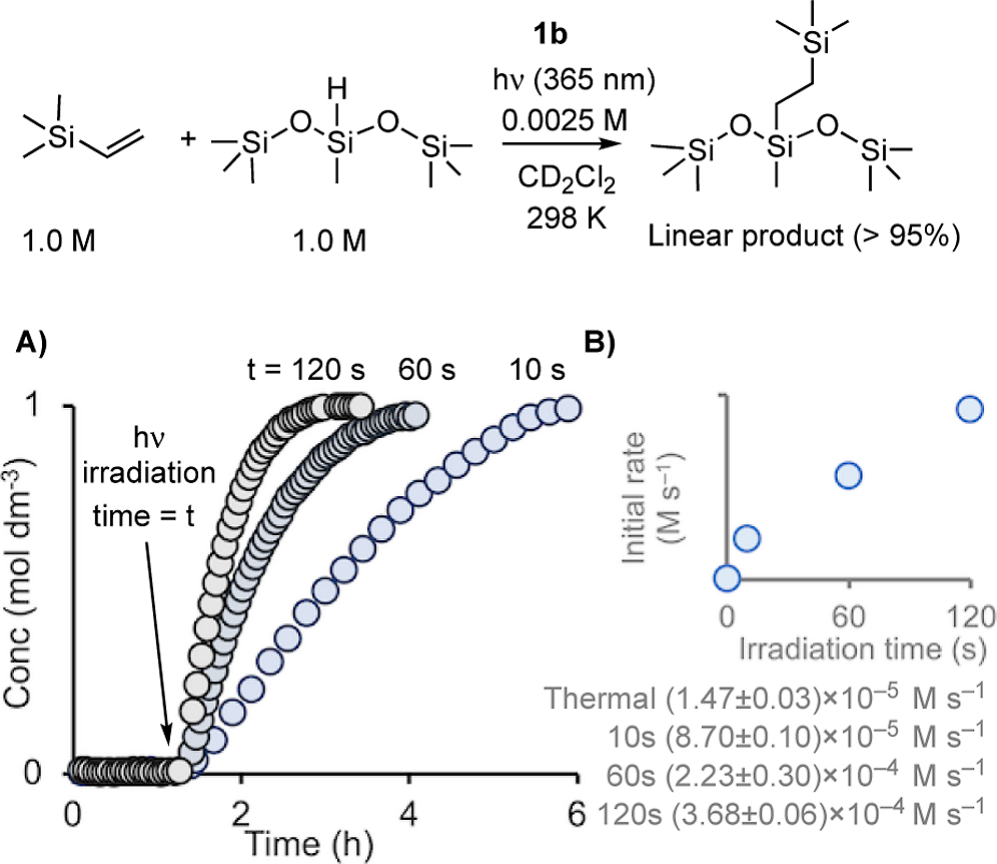
Photoactivated hydrosilylation. (A) Concentration of linear product versus time using precatalyst **1b** (298 K, 0.25 mol %, CD_2_Cl_2_, 1 M substrates) and irradiation at 365 nm for 10 s, 60, and 120 s after ∼1.5 h without irradiation. (B) Initial rates, post induction period, versus irradiation time.

Figure 3A shows the resulting data for these three irradiation times. Immediately apparent is that turnover continues after irradiation has stopped, identifying this as photoactivated catalysis rather than photocatalysis (i.e., one that requires photolysis for turnover). This signals that the activated catalytic species is generated from the precatalyst, in the presence of substrate, by a photochemical process. Using the method of initial rates, measured after the latency period in the pseudo-zero-order regime of catalysis, photoactivation results in a significant rate acceleration (25-fold for 120 s irradiation) compared with the thermally activated precatalyst (Table 1), and this scales approximately linearly with time of irradiation, Figure 3B. This relationship suggests that the hydrosilylation reaction is first order in the active catalyst that is formed after photoactivation. Interestingly, there is a nonzero intercept for these initial rate measurements (Figure 3B), which suggests that a kinetic burst of catalyst generation operates in the photoactivation period. Repeating these experiments using different batches of substrate/solvent showed a small but significant variation in the kinetic profiles. We thus confine quantitative comparisons of rate data to each set of coherent experiments presented that used the same batch of substrates.

**Figure 4:**
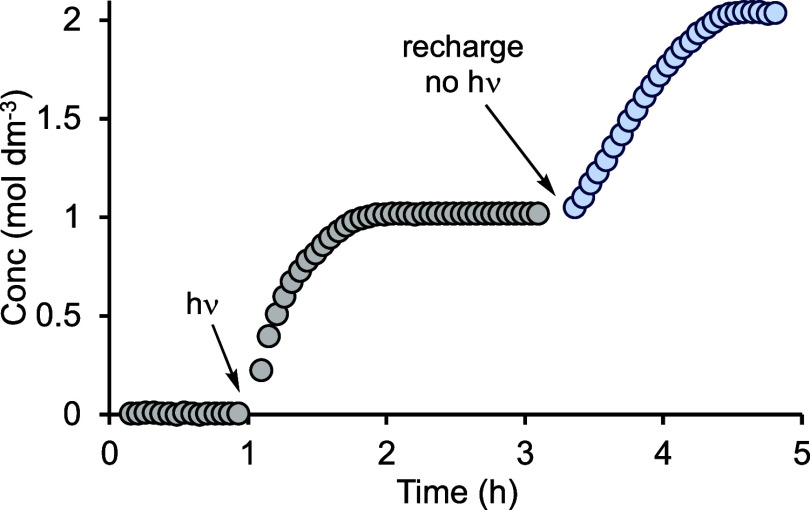
Concentration of linear product versus time using precatalyst **1b** (298 K, 0.0025 M, CD_2_Cl_2_, 1 M substrates) and irradiation at 365 nm for 120 s, after a latency period of 1 h, followed by substrate recharge after 3.4 h.

As for the thermal reactions under photoactivation conditions, post catalysis mixtures show no change in the precatalyst speciation to the detection limit of ^1^H NMR spectroscopy (5%), Figure S32. Confirmation that the active catalyst comes from initial photolysis of the precatalyst is provided by the observation that activity is retained on recharge of solutions with more substrate at the end of catalysis (e.g., after 3.4 h) without further irradiation, Figure 4. While the kinetic profile of the recharged reaction is likely to have a small contribution from the background thermal reaction and catalyst aging, the retention of activity is unambiguous.

The following control experiments were carried out to determine whether the photochemical activation required a substrate. Using our standard conditions of concentration, solutions of **1b** were irradiated for 10 s with various combinations of substrate: none, alkene only, silane only, or both. The remaining substrate partner was then immediately added to the NMR tube and reaction progress was monitored using ^1^H NMR spectroscopy with no further irradiation (Figure S42). No catalysis occurred when just the catalyst is irradiated, while alkene has a more promoting effect than silane. Optimal catalytic turnover occurs when both substrates are present during irradiation. This shows that the generation of the active catalyst requires both substrates, but the effect of the alkene dominates. A similar role of substrates has been noted in the photoactivation of Pt(acac)_2_ as a hydrosilylation catalyst.^22^

Photolysis of precatalyst **1a**(61) in the absence of substrates (0.0025 M, 365 nm, CD_2_Cl_2_) for 10 min revealed low conversion (∼20%) to new species in the ^1^H NMR spectrum. This experiment demonstrates the photoactivity of the precatalyst over a considerably longer photolysis period than is used for hydrosilylation (10 s–120 s irradiation time), for which analysis by ^1^H NMR spectroscopy shows no conversion to new species to the detection limit (∼5%). The ^1^H NMR spectrum suggests that a cis-isomer may be formed,^62^ but its identity remains unresolved.

### 2.5 Photochemically Activated Catalyst **1b**: a Homogeneous Catalyst? Selective Poisoning Experiments and Analysis of Aged Catalyst Samples

**Figure 5:**
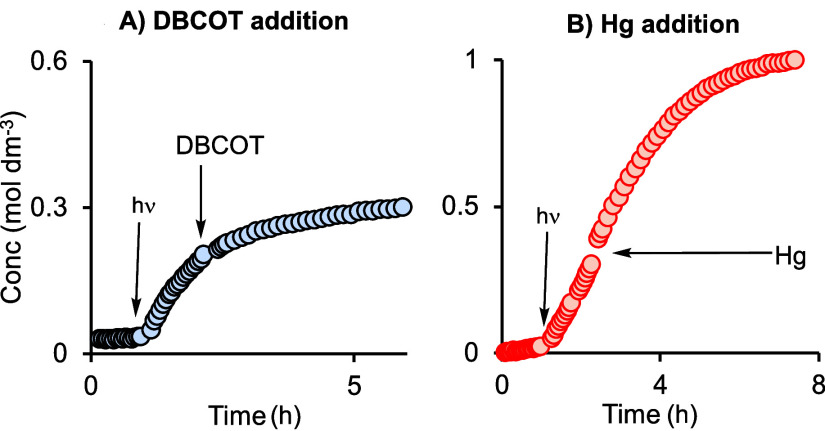
Concentration of linear product versus time using precatalyst **1b** (298 K, 0.25 mol %, CD_2_Cl_2_, 1 M substrates) and irradiation at 365 nm for 10 s, after a latency period of 1 h, followed by addition of (A) DBCOT or (B) Hg, as marked.

As for the thermally activated systems, the Hg- and DBCOT-tests were carried out for the photochemically activated precatalyst. Precatalyst **1b** was irradiated in the substrate mixture, as before, and DBCOT was added after ∼25% conversion. This resulted in a considerable reduction in turnover frequency (Figure 5A) and catalysis did not reach completion, with only ∼30% conversion achieved. In a complementary experiment, Hg was added after ∼35% conversion and the NMR tube vigorously shaken. No significant diminution in the rate of turnover was observed (Figure 5B). These observations are very similar to those of the thermally generated catalysts and signal a homogeneous photochemically activated catalyst.

A competition experiment using a 9:1 ratio of trimethylvinylsilane and the chelating alkene norbornadiene (NBD) resulted in no catalytic turnover, showing that NBD inhibits catalysis—presumably by binding strongly with the Pt-center—similarly to DBCOT. Inhibition by NBD has been reported for Pt(0)-based precatalysts.^13^ Under similar conditions, cyclohexene did not inhibit catalysis but was not hydrosilylated itself.

Analysis of the postcatalysis mixture after the sample had been aged (2 h) using transmission electron microscopy (TEM) and dynamic light scattering (DLS) showed a bimodal distribution of Pt-nanoparticles (20–30 nm and less than 2 nm, Figure S43). The role of Pt-nanoparticles has been widely discussed in hydrosilylation. While some heterogeneous systems are shown to be active,^15,18^ the formation of inactive, or less active, Pt-nanoparticles with molecular precatalysts has been suggested to be a result of catalyst deactivation.^9,10,22^ For the system under discussion here, the Hg- and DBCOT-experiments described above lead us to conclude that a similar scenario is operating and that the active catalyst that is formed on irradiation is homogeneous.

These observations on catalyst latency, photoactivation, activity on recharging, and homogeneity provide a framework to explore the mechanism of the active catalyst using more detailed kinetic methods as described next.

### 2.6 Determining the Order in Silane and Alkene Post Irradiation

**Figure 6:**
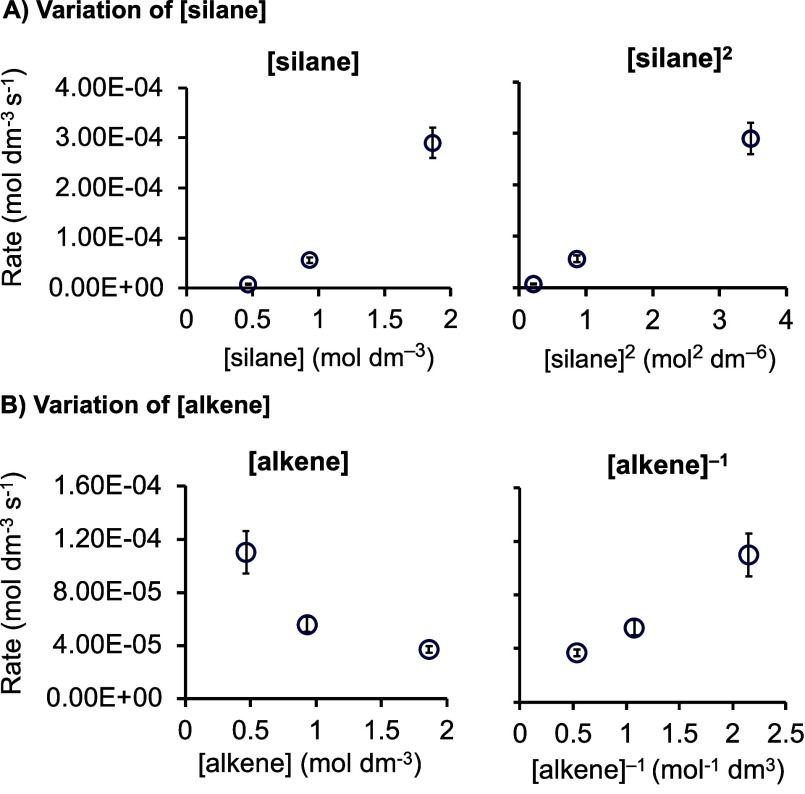
Plots of initial rate (post irradiation) versus concentration determining the order in (A) silane and (B) alkene (298 K, **1b** = 0.0025 M, CD_2_Cl_2_, 10 s irradiation).

Using precatalyst **1b**, the order in alkene (trimethylvinylsilane) and silane (hexamethylsiloxymethylsilane) after irradiation was determined. The method of initial rates was used, with data collected immediately after irradiation (10 s irradiation time) in the pseudo-zero-order regime of turnover. Precatalyst **1b** concentration was kept constant, and [silane] or [alkene] concentrations were varied. Figure 6A shows that plotting the resulting initial rates against [silane] did not provide a linear relationship, while an order of [silane]^2^ provided an acceptable fit. This second-order relationship in [silane] also holds for longer precatalyst irradiation times [120 s, Figure S46]. Figure 6B shows that alkene, in contrast, plays an inhibitory role in turnover, with an order determined to be close to [alkene]^−1^. Equivalent initial rate measurements using precatalyst **1f** showed the same relationship for [silane] and [alkene] (Figures S47–S50), demonstrating the generality of these observation.

The second-order behavior with respect to [silane] has been noted previously by Kühn and co-workers for the hydrosilylation of 1-octene with HSiCl_3_ using Karstedt’s catalyst.^9^ An inverse first order was also determined for the alkene. These orders were explained by a set of pre-equilibria prior to turnover limiting migratory insertion, that involve activation of two silanes at a {Pt(alkene)_2_} fragment, an intermediate that arises from alkene dissociation from a Pt(alkene)_3_ complex. Girolami and co-workers have recently proposed a different explanation,^13^ in that a second equivalent of silane promotes migratory insertion as the turnover limiting step by coordination to form a transient five coordinate intermediate. Related bis-silyl Pt(II) complexes have been postulated as intermediates in alkene hydrosilylation,^17^ and a positive order in silane and inhibition of catalysis by alkene has been noted for other Pt-based catalysts.^10,63^ Seitz and Wrighton^34^ proposed a 2-silicon cycle for a photoactivated cobalt carbonyl system, and Duckett and Perutz proposed a similar cycle for a cyclopentadienyl rhodium system.^35^ In both, the resting state is a metal silyl complex that is formed outside the cycle, and a further molecule of silane undergoes oxidative addition within the cycle. We offer an explanation for the second-order behavior in [silane] for the systems under discussion here, in which the rate of precatalyst activation and turnover are both positively dependent on [silane]. We return to this discussion after the effects of isotopic labeling.

### 2.7 Photochemical H/D Experiments

**Figure 7:**
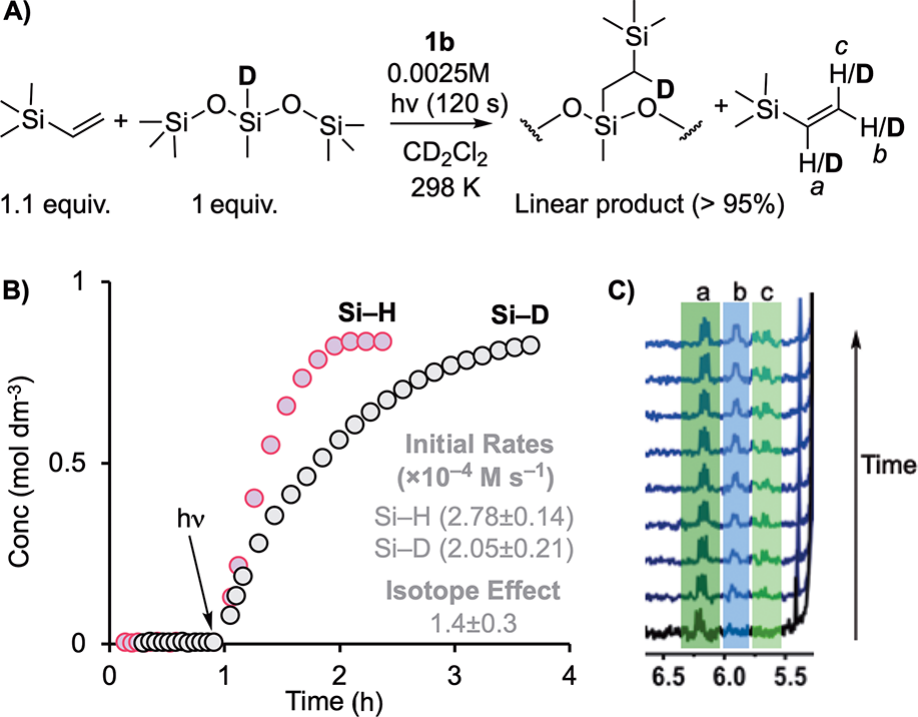
H/D labeling experiments showing product distribution. (A) Catalysis (120 s irradiation time) using an [alkene]/[silane] ratio of 1.1:1. (B) Comparison of temporal profiles using Si–H and Si–D labeled silanes, with measured initial rates and isotope effect; (C) ^2^H NMR of the alkene region (CD_2_Cl_2_ solution) showing the H/D exchange into free alkene.

To provide information on the turnover limiting step, the photoactivated hydrosilylation study was repeated using *d*_1_-hexamethylsiloxymethylsilane (0.8 M), labeled at the Si–D position, and trimethylvinylsilane/catalyst **1b** (see Supporting Information for synthesis). The ratio of alkene/silane was 1.1:1 (Figure 7A). An irradiation time of 120 s was used for experimental expediency, and the reaction was monitored by ^1^H and ^2^H NMR spectroscopy. The temporal evolution of catalysis post irradiation showed that the d-labeled substrate was fully converted, and there was no change in product selectivity,^64^ but turnover was significantly slower (Figure 7B). An isotope effect on the initial rate of product formation of 1.4 ± 0.3 was determined in a side-by-side experiment with protio-substrate. This isotope effect is similar to that measured for Karstedt’s catalyst combined with similar substrates to those used here (1.8), for which reductive elimination is proposed for the turnover-limiting step, that is preceded by Si–H bond breaking.^10^ However, for Karstedt’s catalyst combined with chlorosilanes and either norbornene or 1-octene alkene substrates, significantly larger isotope effects are measured, of 2.4(1) and 3.9(4), respectively. Here, the turnover-limiting step is proposed to be migratory insertion of the hydride to the alkene.^9^

Under these conditions, ^2^H NMR spectroscopy shows that there is H/D exchange into free trimethylvinylsilane. At the early stages of reaction, this favors the internal C–H vinyl bond, but over the course of reaction, all the vinyl C–H bonds undergo H/D exchange (Figure 7C). These observations support all of the following mechanistic conclusions: (i) alkene coordination is reversible, (ii) hydride insertion is reversible, (iii) the overall barrier to give a branched intermediate is higher than for the linear, and (iv) reductive elimination is likely the turnover limiting step. Similar observations have been made for related reactions, such as alkene hydroacylation, which also show similar isotope effects to those reported here (∼1.4).^65,66^

The overall isotope effect of 1.4 likely represents a combination of effects arising from precatalyst activation (see later) and steps in the catalytic manifold. On the catalytic cycle, it is likely that an equilibrium isotope effect (EIE)^67,68^ would result from reversible migratory insertion, and possibly reversible Si–H activation, prior to rate determining reductive elimination. As precatalyst activation may well involve breaking an Si–H bond, this could also result in an isotope effect (a KIE). This complexity makes delineating the individual contributions challenging. An experiment where d-labeled silane is first used as a substrate under photoactivation conditions, and then the system is recharged with protio-silane without photoactivation, reveals that the initial rate of hydrosilylation after recharge is ∼1.3 faster than the initial rate with Si–D (Figure S51). As catalyst aging will contribute to a deceleration in the rate on recharge, this represents a minimum for the isotope effect operating in the catalytic manifold, suggesting only a relatively small isotope effect for catalyst activation.

### 2.8 COPASI Modeling and a Suggested Mechanism. Variation in Catalyst Preactivation

**Scheme 5:**
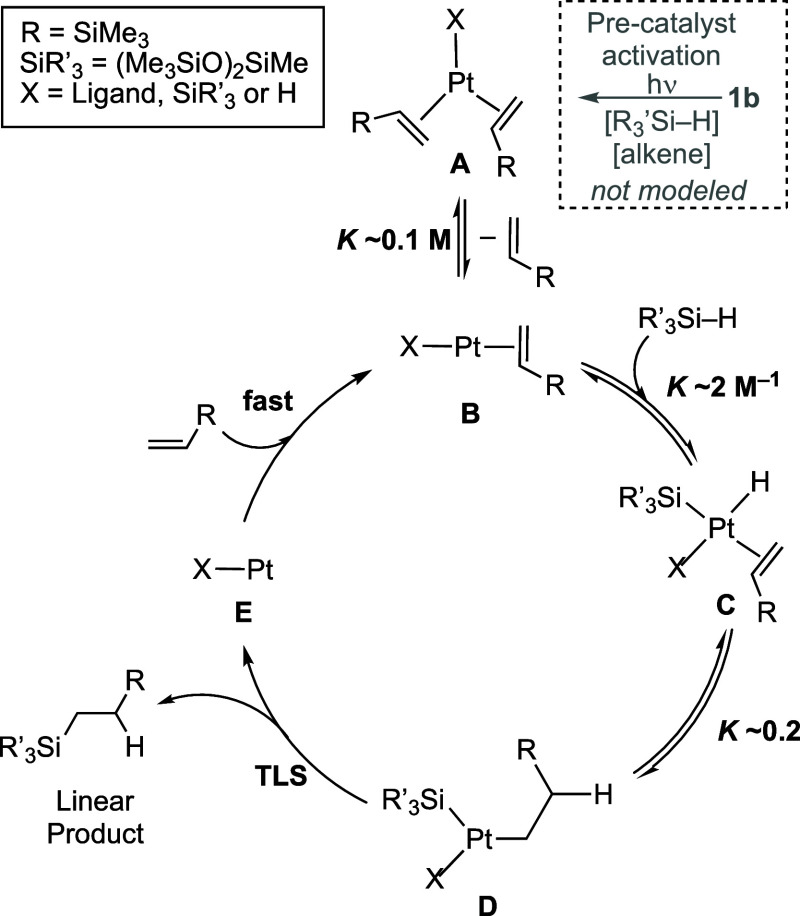
Simplified Catalytic Cycle, Showing the Modeled Elementary Steps, Associated Intermediates, and Selected Equilibrium Constants as Simulated Using COPASI Precise speciation of the catalyst is undetermined (i.e., oxidation state, charge, and identity of X).

With the preceding observations of precatalyst activation, overall order in substrates, and H/D labeling experiments, a reaction mechanism was modeled using COPASI^41^—informed by previous studies on the mechanism of hydrosilylation using Pt-based catalysts.^9,10,13^ Four independent sets of [alkene]/[silane] starting concentrations were holistically and simultaneously modeled against the corresponding experimental data, post irradiation. Experimental conditions were ∼0.5–2 M for each substrate, a constant precatalyst concentration (i.e., 0.0025 M), and a 10 s irradiation time. Both catalyst systems **1b** and **1f** were modeled resulting in very similar kinetic fits (see Supporting Information for **1f**). In the absence of any specific measured rate constants, these models only provide overall relative rates, rather than absolute values, and some consecutive individual steps were telescoped for simplicity to avoid overparameterization. For the same reason, only the principal, linear-product pathway was modeled, while precatalyst activation was not. Scheme 5 shows the defined elementary steps of the model with associated relative equilibria and key intermediates. While the precise speciation of the active catalyst (**A**) is not known (see later), the model captures an endergonic alkene dissociation to form **B**, which undergoes reversible Si–H oxidative addition followed by migratory insertion to give **D**. Turnover-limiting reductive elimination of the linear product is followed by rapid alkene coordination to return **B**. This model also supports the proposed isotope effect of 1.4. Notably, only one equivalent of silane is added in the catalytic cycle (**B** to **C**), while alkene plays a role as both a substrate and an inhibitor (**B** to **A**).

**Figure 8:**
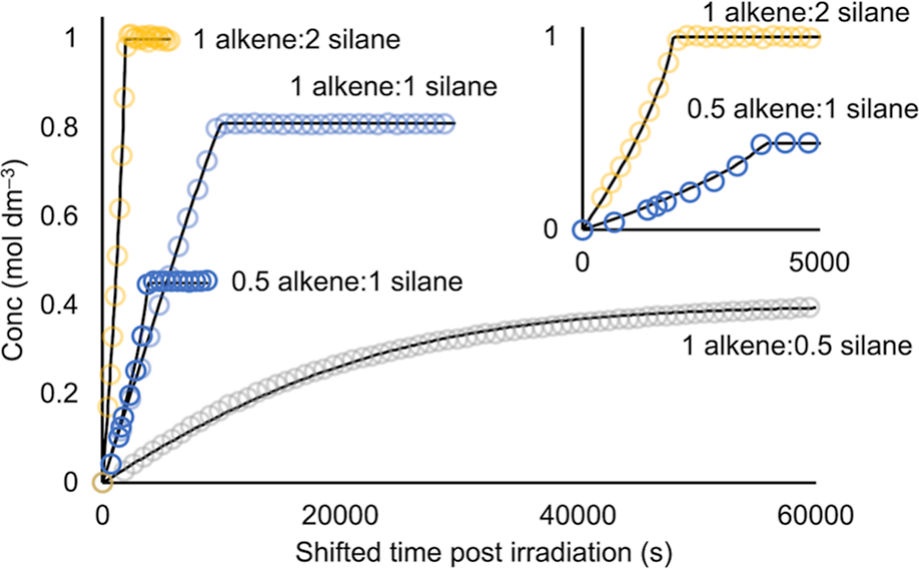
Concentration of linear product versus time for a variety of starting concentrations of alkene and silane substrates (∼0.5 to 2 M) and precatalyst **1b** (0.0025 M, 10 s irradiation time) showing the evolution of the linear product. Open circles = experimental data; solid lines = holistically simulated data derived from the catalytic manifold outlined in Scheme 5. Data is time-shifted to remove the latent periods prior to irradiation. Inset shows the first 5000 s for experiments with an excess of silane that show the increase in rate of turnover with time.

Figure 8 shows the fits of simulated to experimental data for the evolution of the final linear product. These high-quality fits capture a number of nuances, which provide confidence that, holistically, this is a reasonable solution for the catalytic manifold. In particular, the inhibitory role of alkene is captured well in the model. For example, when compared to an initial 1:1 ratio of [alkene]/[silane], for an initial 1:0.5 ratio (i.e., an excess of alkene), the rate of catalysis shows a pronounced deceleration with time. This effect is a consequence of increasing enrichment in alkene relative to silane as catalysis proceeds, which in turn inhibits turnover by pushing the off-cycle equilibrium toward **A**. The opposite effect is observed when alkene is deficient (initial ratios of 1:2 or 0.5:1). Here, as catalysis evolves, the ratio of [silane]/[alkene] increases, and the inhibitory effect of alkene becomes weaker. The consequence of this is that the rate of catalysis increases as the substrates become enriched in silane with each turnover (see Figure 8, inset). When the ratios are balanced and constant throughout the reaction (i.e., ∼1:1 starting ratio) apparent pseudo-zero-order evolution of the linear product is observed. The corollary of this is that even a small initial excess of alkene substrate would be expected to have a decelerating effect near the end of the catalysis. Very similar kinetic profiles have been discussed in detail for the anion-initiated trifluoromethylation of ketones using Me_3_SiCF_3_. As found here, initial rate measurements showed initiator (anion) and ketone to be first order and Me_3_SiCF_3_ approximately inverse order.^69^

At the start of the fitting process, it became apparent that a universal solution for the four different sets of substrate concentration regimes was not possible using the same catalyst concentration. Although the precatalyst concentration was the same (0.0025 M) for all four sets of [silane]/[alkene] ratios, successful modeling required different effective catalyst concentrations, [cat]_effective_, Table 2. To do this, each individual experiment was manually iterated for different [cat]_effective_ values, and the model was simulated holistically over all four data sets using the same set of rate constants. This model also accounts for less than 5% of the active catalyst being formed (i.e., [cat]_effective_ = 15 × 10^–6^ M or lower). The same process was run for precatalyst **1f**, that resulted in a very similar, but different, set of [cat]_effective_ for those particular starting substrate concentrations (Table S7).

**Table 2:** Experimental Details of [Alkene] and [Silane] Used for the COPASI Model Using Catalyst **1b**, and Resulting, Iterated, [cat]_effective_ Used in the Fitting^a^

entry	[alkene]/M	[silane]/M	[cat]_effective_/× 10^–6^ M
1	0.81	0.84	6.0
2	0.79	0.40	2.4
3	1.00	1.94	15
4	0.45	0.97	3.3

a Conditions as in Figure 8.

The growth of the linear product, using the derived rate constants for catalyst **1b** from the fitting of the experimental data, was also simulated for different [cat]_effective_ at fixed [alkene] and [silane] concentrations. This demonstrates that the derived model provides a linear, first-order relationship between the simulated rate of turnover and [cat]_effective_ (Figure S53). This is consistent with the experimental observations (Figure 3) of a linear relationship between the precatalyst irradiation time and initial rate of turnover.

**Figure 9:**
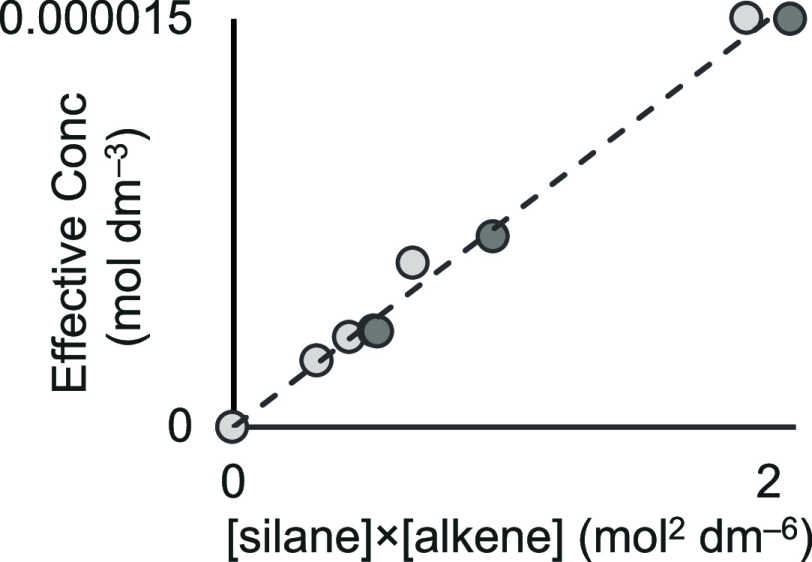
Relationship of [alkene] and [silane] to [cat]_effective_ for precatalysts **1b** (light gray) and **1f** (dark gray).

Insight into the role of substrates in precatalyst activation comes from plotting [cat]_effective_ used in the successful model against the experimental data of [silane] × [alkene], leading to a linear relationship (Figure 9). This diagram also includes data for precatalyst **1f**, showing that this relationship holds for both systems. This suggests that the amount of precatalyst that is converted to the active catalyst, i.e., [cat]_effective_, is dependent on each set of starting substrate concentrations, implicating the role of substrates in both precatalyst activation and productive turnover. This is consistent with control experiments described earlier that show the fastest turnover occurs when both alkene and silane solutions are present during precatalyst irradiation.

The apparent second order in [silane] that is derived from the initial rate experiments can now be explained as this reflects a precatalyst photoactivation that is first order in [silane] (not modeled in COPASI) coupled with productive catalysis after photoactivation also being first order in [silane], i.e., overall pseudo [silane]^2^. This explanation of the second-order behavior in [silane] using photoactivated Pt(sal)(ppy) is different from that of Kühn^9^ and Girolami^13^ for Karstedt’s catalyst under thermal conditions that requires two equivalents of silane per catalytic turnover. Supporting this different mechanistic scenario using Pt(sal)(ppy) is that COPASI modeling of two equivalents of silane being involved in the catalytic cycle, while keeping the concentration of [cat] fixed at 2.5 × 10^–6^ M, did not result in an acceptable fit (Figure S54).

The kinetics indicate an apparent positive order in alkene that controls precatalyst activation and thus [cat]_effective_. This is more difficult to reconcile with the inverse order in [alkene] for overall catalysis that comes from the initial rate experiments. One explanation is the involvement of a trace activator present in the alkene, the concentration of which would scale proportionately with [alkene], and that photoactivation is zero-order in alkene.^70^ This hypothesis is also consistent with the burst in catalysis measured for different irradiation times but the same [alkene] and [silane] (Figure 3B), which would result from a small, but consistent, concentration of an additional activator that operates under photoactivation conditions. The role of trace contaminants in modifying catalysis has been reported before.^71−74^

### 2.9 Comments on Precatalyst Activation and the Overall Mechanism

**Scheme 6:**
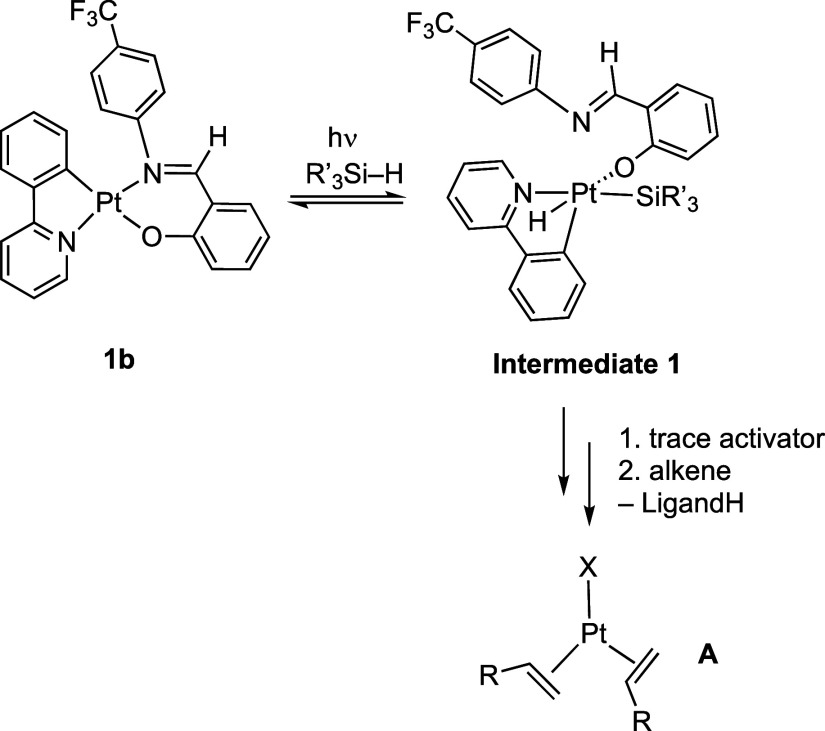
Suggested Mechanism of Photoactivation of the Precatalysts. X = SiR_3_′ or H. The Overall Charge/Pt Oxidation State of A Is Not Defined

While the identity of the actual catalyst remains unresolved due to the very low precatalyst conversion, it must be a highly active species that operates at the ppm-levels. A tentative mechanism for photopromoted catalyst activation is shown in Scheme 6. We propose that initial activation occurs under photochemical conditions, likely via coordination and activation of the silane, to generate **Intermediate 1**. This could be aided by ligand hemilability, and cis/trans isomerization. Similar activation manifolds that are aided by ligand dissociation (e.g., a change from κ^2^ to κ^1^ binding) have also been proposed for the formation of the active catalysts in Pt(acac)_2_-based photoinitiated systems.^22,24,29,75^ The formation of the active catalyst could also occur by a pre-equilibrium trans to cis isomerization of the salicylaldimine ligand in the precatalyst.^62^ We see no evidence (from ^1^H NMR studies) for the initial formation of an alkene adduct under these conditions, such as reported for the related Pt(hfac)_2_ complex (hfac = 1,1,1,5,5,5-hexafluoro-2,4-pentanedionato) that forms a five coordinate ethene adduct on photoreaction at 350 nm, albeit in the absence of silane.^24^ The challenges associated with determining trace levels of highly active catalyst have been discussed for other catalyst systems.^76^

**Figure 10:**
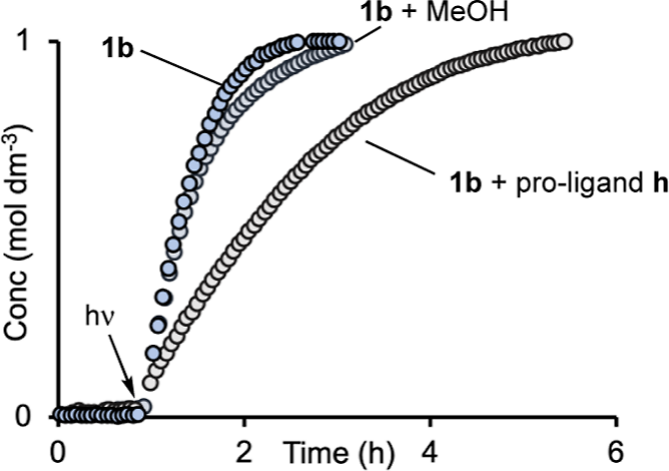
Concentration of linear product versus time using precatalyst **1b** (298 K, 0.0025 M, CD_2_Cl_2_, 1 M substrates) and irradiation at 365 nm for 120 s, after a latency period of 1 h, in the presence of 10 equiv (compared with **1b**) of MeOH and 2 equiv of proligand **h**. The data for **1b** has been time shifted by 0.4 h to allow for a comparison of postirradiation profiles.

One possible role of a putative trace activator is to protonate off the ligand(s) in the precatalyst. To probe this, addition of a 10-fold excess of MeOH to precatalyst **1b** (0.25 mol %) and irradiation for 120 s^77^ after the latency period resulted in no significant change in the initial rate, Figure 10 [3.57 (±0.02) × 10^–4^ M s^–1^ compared with 3.68 (±0.06) × 10^–4^ M s^–1^], and no change in selectivity or overall TON. ^1^H NMR spectroscopy at the end of catalysis revealed that precatalyst **1b** is essentially unchanged in concentration to the detection limit of the experiment (∼5%). Combined, these observations suggest that irreversible protonation can be discounted in an activation (or deactivation) step. Addition of a 2-fold excess of protonated proligand **h** (Scheme 3) to precatalyst solutions of **1b** resulted in considerably slower catalysis post 120 s irradiation [initial rate = 1.27(±0.02) × 10^–4^ M s^–1^]. There was no change to the precatalyst **1b** remaining at the end, i.e., complex **1h** was not formed. This suggests that a pre-equilibrium ligand exchange process is not occurring in the activation process. The approximate ^2^/_3_ decrease in initial rate is best accounted for by the high molar absorption coefficient of the salicylaldimine proligands at 365 nm (Table S2) that would act to slow down photoactivation of the precatalyst, and thus lower[cat]_effective_.

We can, however, discount the role of radicals within the limits of the TEMPO method^78,79^ as addition of 10 equiv of the radical trap TEMPO to a precatalyst solution of **1b** resulted in no significant change in the rate, TON, or selectivity (Figure S39). Photocatalytic hydrosilylation by radical species arising from the Mn_2_(CO)_10_ precatalyst has been shown to be quenched by TEMPO addition.^40^

**Scheme 7:**
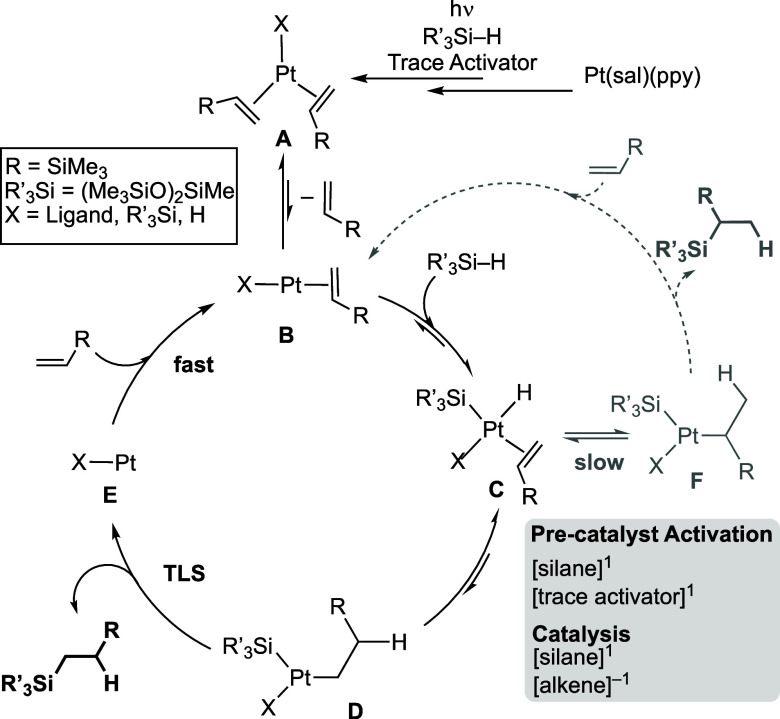
Suggested Mechanism for Hydrosilylation

Scheme 7 presents the overall mechanistic landscape for the photoactivated hydrosilylation using Pt(sal)(ppy) precatalysts that incorporate catalyst activation and the linear/branched product selectivity and combines the mechanistic observations, kinetics, and COPASI simulations. For the systems under consideration here, the identity of X and the oxidation state of the active Pt-species formed in trace quantities remain to be determined. A Pt(0), Pt(alkene)_3_, or a Pt(II) species such as [XPt(alkene)_2_]^+^ (X = ligand, H, SiR_3_′) are possibilities. Pt(II)/Pt(IV) or Pt(0)/Pt(II) cycles have both been postulated for hydrosilylation.^9,13,17^

### 2.10 1-Octene and Styrene Substrates

**Scheme 8:**
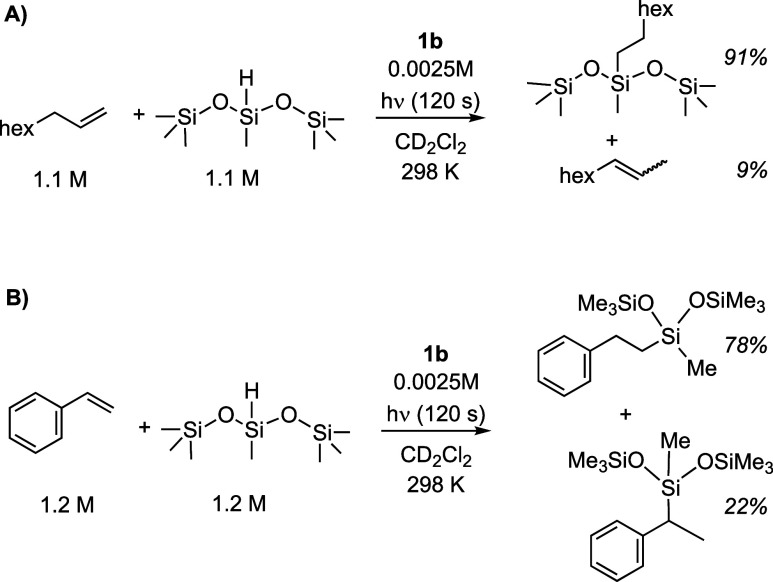
Photoactivated Hydrosilylation of (A) 1-Octene and (B) Styrene

The photoactivated hydrosilylation of 1-octene and styrene using precatalyst **1b** has been examined, to probe selectivity for the linear product over alkene isomerization (1-octene) or the branched hydrosilylation product (styrene).^4^ Under the conditions of 0.25 mol % **1b** and 120 s irradiation time, complex **1b** promotes hydrosilylation of both substrates (Scheme 8 and Figures S33 and S35). A latency period is observed before photoactivation. For 1-octene, while only the linear product was formed, significant isomerization to form internal alkenes was observed; in contrast a mixture of linear and branched isomers of the final products are observed for styrene. These regioselectivities are consistent with competitive insertion of the hydride to form the branched intermediate **F** (Scheme 7) and are similar to those reported using Karstedt’s catalyst with these substrates.^9,80^

### 2.11 Practical Applications of Photoactivated Pt(sal)(ppy) Precatalysts: ppm-Level Catalyst Loadings and Cross-Linked Polysilicones

**Scheme 9:**
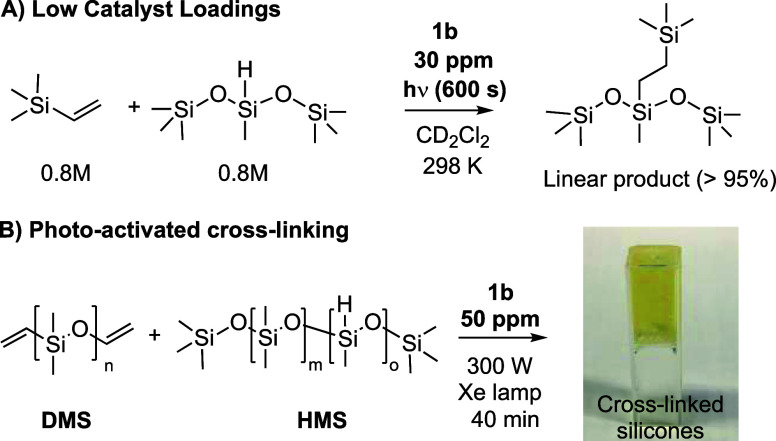
Photoactivated Hydrosilylation Using Catalyst **1b** at Different Loadings. (A) 30 ppm of Catalyst. (B) Commercial Formulation, Showing Inverted Cuvette Postcatalysis

To demonstrate the wider utility of these catalysts, we have studied the application of precatalyst **1b** at very low loadings, using 30 ppm (i.e., 0.003 mol %, 2.5 × 10^–5^ M) and a concomitantly extended irradiation time of 600 s (Scheme 9A). Under these conditions, the now optically dilute precatalyst **1b** promotes complete turnover in 2.6 h at 298 K (TON_app_ = 32,000, TOF_app_ = 12,300 h^–1^) for the reaction between trimethylvinylsilane and hexamethylsiloxymethylsilane. A latent time of ∼1 h was also established prior to irradiation. While these TON_app_ and TOF_app_ do not match some of the very best reported for other Pt-based hydrosilylation catalysts, by at least an order of magnitude,^13,81,82^ the air-stability, operation at room temperature, and photoactivated nature of the precatalyst **1b**, when combined with such low loading, are noteworthy.

Precatalyst **1b** was also tested in the photocuring of commercial substrates, methylhydrosiloxane/dimethylsiloxane copolymer (**HMS**, ∼1000 g/mol) and vinyl-terminated polydimethylsiloxane (**DMS**, ∼6000 g/mol), that results in cross-linked polysilicone (Scheme 9B). The conditions used were chosen to capture those used in a commercial setting. A 50 ppm loading of precatalyst **1b** was used in conjunction with a commercial 300 W Xe arc lamp (Oriel Instruments. Model 6259) that was placed 110 mm away from a cuvette containing the 1:1 substrate formulation (2 g total in 0.1 cm^3^ CH_2_Cl_2_), precatalyst, and stirrer bar. The reaction was deemed complete when the cross-linking in the product resulted in a sufficiently viscous solution to stop the stirrer bar and allow the contents of the cuvette to be inverted. No precautions for the ingress of air or water were taken, and the sample was not cooled. Under these conditions, precatalyst **1b** promoted curing in 40 min. As a comparison, the commercially relevant Pt(CpMe)Me_3_^19^ takes 3 min to cure this formulation. While **1b** clearly does not match this in terms of curing time, it does offer advantages of air-stability, low toxicity, and ease of synthesis.

## 3 Conclusions

Photoactivated catalysts that allow for time- and light-gated hydrosilylation are important for a variety of industrially related photocuring processes. Here, we show that simple-to-prepare, air-stable, Pt(salicylaldimine)(phenylpyridyl) complexes are effective precatalysts for photoactivated hydrosilylation, some of which show appreciable latency under thermal conditions. Irradiation at 365 nm, for only 10 s, results in the formation of very active homogeneous catalysts that promote the efficient and selective hydrosilylation of trimethylvinylsilane and hexamethylsiloxymethylsilane. This result contrasts with other photocatalysts for hydrosilylation that require irradiation during catalysis. These precatalyst systems allow for a detailed kinetic study of photoactivated alkene hydrosilylation, the data from which are of sufficient quality to allow for successful simulation and interrogation of the catalytic cycle. Combined, the resulting kinetic and mechanistic observations point to a catalytic cycle where the turnover limiting step is reductive elimination of the linear product, while precatalyst activation is dependent on the concentrations of solutions of silane and alkene used.

The formation of only trace amounts of active catalyst represents a challenge in determining the active species for this photochemically promoted hydrosilylation. Nevertheless, the modular nature of these precatalysts will allow for targeted variation in the steric and electronic influence of the ligand and tuning of precatalyst activation. Such variation, in conjunction with time-resolved spectroscopic methods, may also allow for the identity and mechanism of formation of the active component to be determined. Of course, any improvements in precatalyst activation would need to be balanced with appropriate thermal latency. The development of a bench-stable, low-toxicity, precatalyst system that combines latency with very high levels of activity and selectivity for commercially relevant hydrosilylation, all on only brief irradiation, is a clear goal moving forward.

## 4 Selected Experimental Details

**Figure 11:**
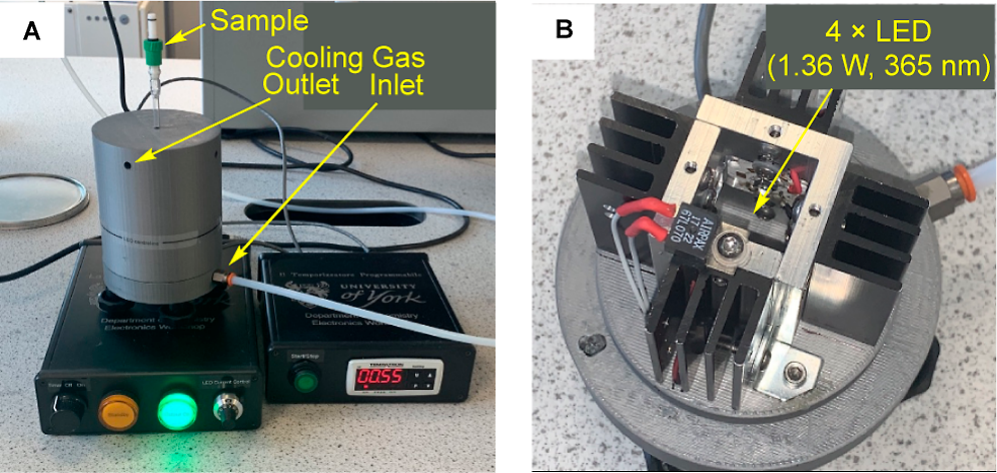
(A) UV-reactor set up, with an NMR tube in situ. (B) View of the reactor cavity showing the arrangement of the LEDs.

To assess and compare the catalytic activity of precatalysts **1a**–**1h** in the model hydrosilylation reaction, a series of NMR experiments were conducted. For each experiment, the precatalyst (0.0025 M) was loaded into a J. Youngs NMR tube, and to it was added *d*_2_-dichloromethane, along with hexamethylsiloxymethylsilane (0.8–1.0 M), trimethylvinylsilane (0.8–1.0 M), and mesitylene (0.10 M) from a stock solution. The sample was inserted into the NMR spectrometer, and an array of ∼20 ^1^H NMR spectra was acquired to determine the thermal latency period over ∼1 h. The sample was then removed from the spectrometer and irradiated using the bespoke 365 nm LED for either 10, 60, or 120 s before being returned to the spectrometer, Figure 11 (note that this step is omitted for experiments conducted under thermal conditions). The UV photoreactor comprised four Osram LZ1-00UV0R LEDs (radiant flux 1.36 W, emission centered at 365 nm with a full width at half-maximum of 11 nm) mounted vertically in a square array to surround a 5 mm NMR tube at a distance of 8.5 mm. The angular distribution at half-maximum radiant flux is ca. 60°. Cooled compressed air was purged through the reactor housing, and a thermocouple located close to the NMR tube showed that there was only a minimal increase in temperature during photolysis (maximum of 2 °C rise over 160 s). The controller provided power and timing control (10–120 s). An array of 10–100 ^1^H NMR spectra were acquired with appropriate delays between each acquisition (depending on the catalytic activity), and the concentrations of hexamethylsiloxymethylsilane, trimethylvinylsilane, and beta product were calculated using the absolute quantitation method. The absolute NMR integrals of the Si**H** (δ 4.6), olefinic C**H** (δ 6.2), and Si–C**H**_2_–C**H**_2_–Si (δ 0.40) resonances were compared with the (C**H**_3_)_3_ (δ 2.3) resonance of the internal standard. The kinetics were probed using the initial rates method, by monitoring the rate of linear product formation over the first 3–5 data points after catalytic activity had been established. For the thermal profiles with an associated induction period, the rate was measured after the induction period at the maximum rate of turnover.

## Supporting Information Available

Full details of the experimental methods, including general techniques and synthesis, characterizations including NMR, UV-vis, TEM, and DLS studies, details of in situ catalysis, poisoning experiments, kinetic plots, and COPASI modeling (PDF)
Single-crystal X-ray diffraction data complex **1b** (CIF)
Single-crystal X-ray diffraction data complex **1f** (CIF)
Single-crystal X-ray diffraction data complex**1g** (CIF)

## Supplementary Material

cs4c01353_si_001.pdf

cs4c01353_si_002.cif

cs4c01353_si_003.cif

cs4c01353_si_004.cif
