# Supplementary material for: Platinum(II) Phenylpyridyl Schiff Base Complexes as Latent, Photoactivated, Alkene Hydrosilylation Catalysts
Source: ACS Catal. 2024 Apr 30;14(10):7492–505. doi: 10.1021/acscatal.4c01353 (PMC11106775; doi:10.1021/acscatal.4c01353)
Supplement: Supplementary file 1 — cs4c01353_si_001.pdf [file cs4c01353_si_001.pdf]

Electronic Supporting Information

Platinum(II) Phenylpyridyl Schiff Base Complexes as Latent, Photoactivated, Alkene Hydrosilylation Catalysts

Helena G. Lancaster,[a] Joe C. Goodall,[a] Samuel P. Douglas,[b] Laura J. Ashfield,[b] Simon B. Duckett,[a]* Robin N. Perutz,[a]* Andrew S. Weller.[a]*

[a]Department of Chemistry, University of York, Heslington, York, YO10 5DD (UK)

[b]Johnson Matthey Technology Center, Blounts Ct Rd, Sonning Common, Reading RG4 9NH (UK)

Corresponding author email addresses

andrew.weller@york.ac.uk

simon.duckett@york.ac.uk

robin.perutz@york.ac.uk

1 Experimental ... 4
1.1 General Techniques ... 4
1.2 Synthesis ... 6
1.2.1 [Pt{NC_5H_4-6-(4'-R-C_6H_3)}Cl(κ_1-S-dmso)] complexes ... 6
1.2.2 Schiff base ligands ... 9
1.2.3 Pt(sal)(ppy) complexes ... 16
1.2.4 Synthesis of d_1-hexamethylsiloxymethylsilane ... 25
2 NMR spectra ... 26
2.1 NMR spectra of [Pt{NC_5H_4-6-(4'-R-C_6H_3)}Cl(κ_1-S-dmso)] complexes ... 26
2.1.1 1-dmso ... 26
2.1.2 2-dmso ... 28
2.2 NMR spectra of Pt(sal)(ppy) complexes ... 30
2.2.1 NMR spectra of 1a ... 30
2.2.2 NMR spectra of 1b ... 32
2.2.3 NMR spectra of 1c ... 35
2.2.4 NMR spectra of 1d ... 37
2.2.5 NMR spectra of 1e ... 39
2.2.6 NMR spectra of 1f ... 41
2.2.7 NMR spectra of 1g ... 44
2.2.8 NMR spectra of 1h ... 47
2.2.9 NMR spectra of d_1-hexamethylsiloxymethylsilane ... 49
3 UV-Vis Spectra ... 51
3.1 dmso complexes ... 51
3.2 Schiff base ligands ... 52
3.3 Pt(sal)(ppy) complexes ... 53
4 In-situ catalysis ... 54
4.1 Method for in-situ reaction monitoring ... 54
4.2 Thermally activated catalysis ... 56
4.3 Photoactivated catalysis ... 57
4.4 ^{1}H NMR data of precatalyst after photoactivation and catalysis ... 58
4.5 Substrate Scope ... 59
4.5.1 Oct-1-ene ... 59
4.5.2 Styrene ... 61
4.6 Addition of methanol ... 63
4.7 Addition of a different Schiff base ligand ... 63

4.8 Addition of TEMPO 64
5 Poisoning experiments for thermally activated catalysis 64
5.1 Hg poisoning 64
5.2 DBCOT poisoning 65
6 Adding substrates before and after irradiation 66
7 TEM images 67
8 Dynamic light scattering 67
9 Determining order in silane and alkene post irradiation 68
9.1 Precatalyst 1b order in silane (post 120 s irradiation) 68
9.2 Precatalyst 1f order in silane (post 10 s irradiation) 70
9.3 Precatalyst 1f order in alkene (post 10 s irradiation) 72
10 Kinetic Isotope Effect 74
10.1 Recharges 74
11 COPASI simulations 75
12 Crystallographic Information 79
13 References 81

1 Experimental

1.1 General Techniques

Dichloromethane-d_2 (CD_2Cl_2), hexamethylsiloxymethylsilane and vinyltrimethylsilane were dried overnight with CaH_2 before vacuum transfer and subsequent degassing by three freeze-pump-thaw cycles and stored over 3 Å molecular sieves. All NMR samples for the in-situ reaction monitoring experiments were prepared under a nitrogen atmosphere using standard Schlenk line and glove-box techniques.

Commercially sourced solvents and reagents were purchased from Acros Organics, Alfa Aesar, Fisher Scientific, Fluorochem or Sigma-Aldrich and used as received unless otherwise noted. The synthesis of the [Pt{NC_5H_4-6-(4'-R-C_6H_3)}Cl(κ_1-S-dmso)] and Pt(sal)(ppy) complexes was performed under a nitrogen atmosphere despite the air stability of the complexes, the main concern being the oxidative and thermal stability of reaction intermediates at elevated temperatures (< 110 °C).

The UV photoreactor comprised four Osram LZ1-00UV0R LEDs (radiant flux 1.36 W, emission centred at 365 nm with full width at half maximum 11 nm) mounted vertically in a square array to surround a 5 mm NMR tube at a distance of 8.5 mm. The angular distribution at half-maximum radiant flux is ca. 60 degrees. Cooling compressed air was purged through the reactor housing, and a thermocouple located close to the NMR tube showed that there was only a minimal increase in temperature during photolysis (maximum of 2 °C rise over 160 s). The controller provided power and timing control (10-120 s).

Solution state NMR spectra were recorded on a Bruker Avance III 500 MHz NMR spectrometer or a Bruker Ultrashield 400 MHz or 600 MHz NMR spectrometer at 298 K unless otherwise specified. Residual protio solvent was used as a reference for ^1H and $^{13}C\{^1H\}$ NMR spectra in deuterated solvent samples. ^{19}F spectra were externally referenced to $CFCl_3$. All chemical shifts (δ) are quoted in ppm and coupling constants (J) in Hz. NMR assignments were aided by 2D spectra ($^1H,^1H$-COSY, $^1H,^{13}C$-HSQC, $^1H,^{13}C$-HMBC) where required. Coupling

constants to ^{195}Pt are listed where satellites are observed. The satellites are generally quite broad. For the in-situ ^{1}H NMR hydrosilylation experiments, the time between FID encodings (d1) was set to 45 s to ensure accurate quantitative analysis.

All UV-vis absorption spectra were recorded using an Evolution Array UV-vis spectrophotometer, with a window of 190–1100 nm. Data was processed using VISIONcollect software. 'Background' absorption spectra of the solvents used were subtracted from sample spectra. Herein, the wavelengths of maximum absorption (λ_{max}) are given in nm, and molar absorption coefficients (ε) are reported in $mol^{-1}\ dm^{3}\ cm^{-1}$.

Electrospray Ionisation Mass Spectrometry (ESI-MS) was carried out using a Bruker compact® Time of Flight mass spectrometer by Mr Karl Heaton at the University of York. All platinum complexes are quoted for ^{195}Pt. Elemental analyses were conducted by Dr Graeme McAllister and Dr Scott Hicks at the University of York, or by Dr Orfhlaith McCullough at London Metropolitan University.

TEM images we collected at the University of York Jeol Nanocentre using a EOL 2010 TEM conventional (2Å) High Resolution TEM.

Dynamic light scattering (DLS) was conducted using a Malvern Panalytical Zetasizer Ultra/Pro instrument equipped with a laser with a wavelength of 633 nm and a detector oriented at 173º to the incident radiation. Sample measurements were performed at 25 °C and the results were processed using the Zetasizer software application V. 7.01.

Details of the X-ray diffraction methods are provided with crystallographic information on p. S79

1.2 Synthesis

1.2.1 [Pt{NC_5H_4-6-(4'-R-C_6H_3)}Cl(κ_1-S-dmso)] complexes

All dmso complexes were synthesised in the same way, via a literature reported procedure.[1,2] For **1-dmso**, K_2PtCl_4 (1.00 g, 2.41 mmol) solubilised in the minimum amount of hot water was added to a solution of 2-phenylpyridine (0.376 g, 2.42 mmol) in acetic acid (100 mL). The mixture was stirred under a nitrogen atmosphere and heated under reflux overnight. The orange precipitate was recovered by filtration and washed with water (10 mL) to afford crude [Pt(*N^C*)(μ-Cl)]$_2$, which was used in the next step without further purification. The [Pt(*N^C*)(μ-Cl)]$_2$ complex (590 mg, 0.762 mmol) was then dissolved in dmso (10 mL) and heated to reflux with stirring for 1 h (4 h for **2-dmso**). The solvent was removed *in vacuo* to afford **1-dmso** in good yield (604 mg, 1.30 mmol, 83%).

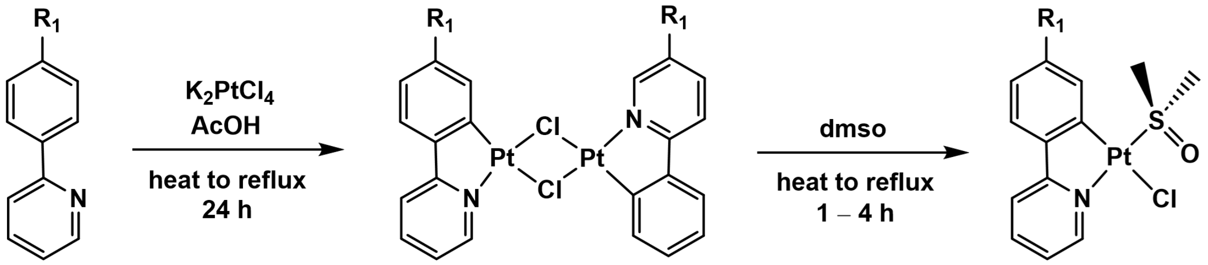

Scheme S1. Synthetic route towards **1-dmso** (R_1 = H) and **2-dmso** (R_1 = OMe).

1-dmso yield (604 mg, 1.30 mmol, 83%).

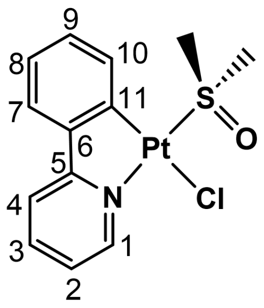

^{1}H NMR (600 MHz, d_2-dichloromethane, 298 K): δ 9.61 (d, 1H, $^3J_{H\text{-}H}$ = 5.9 Hz, $^3J_{H\text{-}Pt}$ = 35.6 Hz, H-1), 8.30–8.28 (m, 1H, $^3J_{H\text{-}Pt}$ = 43.7 Hz, H-10), 7.91 (t, $^3J_{H\text{-}H}$ = 7.7 Hz, 1H, H-3), 7.78 (d, 1H, $^3J_{H\text{-}H}$ = 8.0 Hz, H-4), 7.57–7.54 (m, 1H, H-7), 7.27 (t, 1H, $^3J_{H\text{-}H}$ = 6.8 Hz, H-2), 7.20–7.17 (m, 2H, H-8/9), 3.60 (s, 6H, $^3J_{H\text{-}Pt}$ = 23.4 Hz, 2 x CH_3).

^{13}C{^{1}H} NMR (151 MHz, d_2-dichloromethane, 298 K): δ 166.5 (s, C-5/6/11), 150.5 (s, $^2J_{C\text{-}Pt}$ = 25.1 Hz, C-1), 145.2 (s, C-5/6/11), 141.1 (s, C-5/6/11), 134.6 (s, $^2J_{C\text{-}Pt}$ = 50.2 Hz, C-10), 131.0 (s, $^2J_{C\text{-}Pt}$ = 50.2 Hz, C-8/9), 125.6 (s, C-8/9), 124.3 (s, $^3J_{C\text{-}Pt}$ = 41.7, C-7), 122.6 (s, $^3J_{C\text{-}Pt}$ = 29.3 Hz, C-2), 119.3 (s, $^3J_{C\text{-}Pt}$ = 39.6 Hz, C-4), 47.7 (s, $^2J_{C\text{-}Pt}$ = 60.1 Hz, 2 x CH_3).

These NMR data are in accordance with the literature reported data.[3]

ESI-MS (CH_2Cl_2): m/z $[M\text{-}Cl^-]^+$ 427.0434 (calc. 427.0439) with the correct isotope pattern.

UV-Vis (CH_2Cl_2): λ_{max}/nm [log(ε dm^3 mol^{-1} cm^{-1})] 242 (4.77), 282 (4.55), 316 (4.22), 328 (4.22).

2-dmso yield (209 mg, 0.42 mmol, 39%).

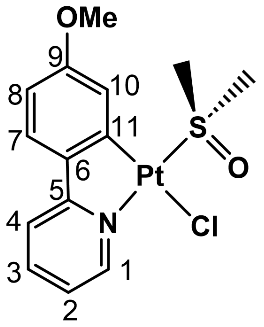

^{1}H NMR (600 MHz, d_2-dichloromethane, 298 K): δ 9.56 (d, 1H, $^3J_{H\text{-}H}$ = 5.9 Hz, $^3J_{H\text{-}Pt}$ = 31.3 Hz, H-1), 7.92 (d, 1H, $^4J_{H\text{-}H}$ = 2.5 Hz, $^3J_{H\text{-}Pt}$ = 52.3 Hz, H-10), 7.83 (t, 1H, $^3J_{H\text{-}H}$ = 7.8 Hz, H-3), 7.62 (d, 1H, $^3J_{H\text{-}H}$ = 8.2 Hz, H-4), 7.48 (d, 1H, $^3J_{H\text{-}H}$ = 8.5 Hz, H-7), 7.16 (t, 1H, $^3J_{H\text{-}H}$ = 6.7 Hz, H-2), 6.72 (dd, $^3J_{H\text{-}H}$ = 8.5 Hz, $^4J_{H\text{-}H}$ = 2.5 Hz, H-8), 3.83 (s, 3H, –OCH_3), 3.76 (s, 6H, $^3J_{H\text{-}Pt}$ = 21.3 Hz, 2 x CH_3).

^{13}C{^{1}H} NMR (151 MHz, d_2-dichloromethane, 298 K): δ 166.4 (s, C-5), 161.7 (s, C-9), 150.1 (s, $^2J_{C\text{-}Pt}$ = 22.3 Hz, C-1), 143.0 (s, C-6/11), 140.8 (s, C-3), 137.7 (s, C-6/11), 125.7 (s, $^3J_{C\text{-}Pt}$ = 49.2 Hz, C-7), 121.2 (s, $^3J_{C\text{-}Pt}$ = 30.0 Hz, C-2), 119.3 (s, $J_{C\text{-}Pt}$ = 54.0 Hz, C-10), 118.6 (s, $^3J_{C\text{-}Pt}$ = 38.6 Hz, C-4), 111.6 (s, C-8), 55.9 (s, –OCH_3), 47.6 (s, $^3J_{C\text{-}Pt}$ = 58.5 Hz, 2 x CH_3).

These NMR data are in accordance with the literature reported data.[1]

ESI-MS (CH_2Cl_2): m/z $[M\text{-}Cl^-]^+$ 457.0539 (calc. 457.0544) with the correct isotope pattern.

UV-Vis (CH_2Cl_2): λ_{max}/nm [log(ε dm^3 mol^{-1} cm^{-1})] 239 (4.68), 298 (4.59), 336 (4.16), 381 (4.06).

1.2.2 Schiff base ligands

All Schiff base ligands (**L-a** to **L-f**) were prepared by the same procedure as reported in the literature.[4] As an example, for **L-b**, an ethanol solution (15 mL) of salicylaldehyde (1.34 g, 0.011 mol) was added to an ethanol solution (15 mL) of 4-(trifluoromethyl)aniline (1.77 g, 0.011 mol) and stirred at room temperature for 1 h. The mixture was then heated to reflux until complete consumption of the starting materials was observed. Subsequently, ethanol was removed under reduced pressure and crude **L-b** was purified by dissolving in minimal dichloromethane and layering with hexane. The pure material was obtained as a yellow crystalline solid in excellent yield (2.77 g, 0.010 mol, 95%).

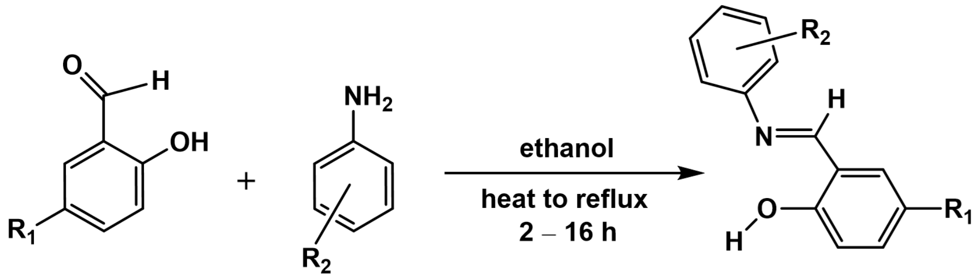

Scheme S2. General synthesis of Schiff base ligands (**L-a** – **L-f**).

L-a yield (2.25 g, 0.011 mol, 95%).

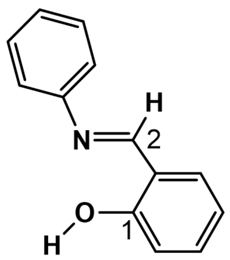

^{1}H NMR (600 MHz, d_2-dichloromethane, 298 K): δ 13.20 (s, 1H, OH), 8.67 (s, 1H, H-2), 7.47-7.45 (m, 3H), 7.40 (1H, t, $^3J_{H-H}$ = 7.6 Hz), 7.33-7.30 (m, 3H), 7.02 (d, 1H, $^3J_{H-H}$ = 8.3 Hz), 6.97 (t, 1H, $^3J_{H-H}$ = 7.6 Hz).

^{13}C{^{1}H} NMR (151 MHz, d_2-dichloromethane, 298 K): δ 163.5 (s, C-2), 161.7 (s, C-1), 149.1 (s, Ar), 133.7 (s, Ar), 133.0 (s, Ar), 130.0 (s, Ar), 127.5 (s, Ar), 121.7 (s, Ar), 119.9 (s, Ar), 119.6 (s, Ar), 117.6 (s, Ar).

These NMR data are in accordance with the literature reported data.[5]

ESI-MS (CH_2Cl_2): m/z $[M+H]^+$ 198.0908 (calc. 198.0913).

UV-Vis (CH_2Cl_2): λ_{max}/nm [log(ε dm^3 mol^{-1} cm^{-1})] 230 (4.99), 270 (4.80), 302 (4.69), 318 (4.73), 340 (4.76).

L-b yield (2.77 g, 0.010 mol, 95%).

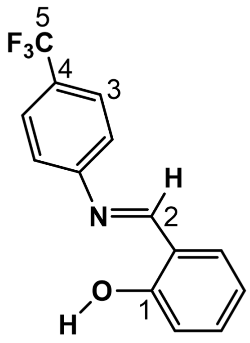

^{1}H NMR (500 MHz, d_2-dichloromethane, 298 K): δ 12.77 (s, 1H, OH), 8.66 (s, 1H, H-2), 7.71–7.70 (m, 2H, H-3), 7.47–7.43 (m, 2H, Ar), 7.42–7.38 (m, 2H, Ar), 7.03–6.97 (m, 2H, Ar).

^{13}C{^{1}H} NMR (126 MHz, d_2-dichloromethane, 298 K): δ 165.4 (s, C-2), 161.8 (s, C-1), 152.4 (s, Ar), 134.4 (s, Ar), 133.4 (s, Ar), 129.3–128.5 (q, $^2J_{C\text{-}F}$ = 32.6 Hz, H-4), 128.1–121.6 (q, $^1J_{C\text{-}F}$ = 272.5 Hz, C-5), 127.2–127.1 (q, $^3J_{C\text{-}F}$ = 3.8 Hz, C-3), 122.1 (s, Ar), 119.9 (s, Ar), 119.6 (s, Ar), 117.7 (s, Ar).

^{19}F NMR (565 MHz, d_2-dichloromethane, 298 K): δ –62.5 (s).

ESI-MS (CH_2Cl_2): m/z $[M+H]^+$ 266.0786 (calc. 266.0787).

These NMR data are in accordance with the literature reported data.[6]

UV-Vis (CH_2Cl_2): λ_{max}/nm [log(ε dm^3 mol^{-1} cm^{-1})] 232 (4.83), 274 (4.70), 340 (4.57).

L-c yield (3.37 g, 0.014 mol, 94%).

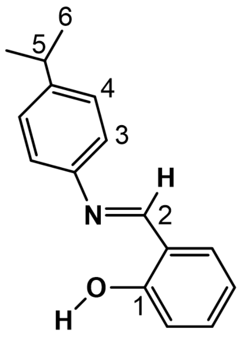

^{1}H NMR (600 MHz, d_2-dichloromethane, 298 K): δ 13.29 (s, 1H, OH), 8.67 (s, 1H, H-2), 7.43 (dd, 1H, $^3J_{H-H}$ = 7.7 Hz, $^4J_{H-H}$ = 1.7 Hz, Ar), 7.37 (t, 1H, $^3J_{H-H}$ = 7.7 Hz, Ar), 7.31–7.30 (m, 2H, H-3/4), 7.27–7.25 (m, 2H, H-3/4), 6.99 (d, 1H, $^3J_{H-H}$ = 8.4 Hz, Ar), 6.95 (t, 1H, $^3J_{H-H}$ = 7.4 Hz, Ar), 2.95 (sept, 1H, $^3J_{H-H}$ = 6.9 Hz, H-5), 1.28 (d, 6H, $^3J_{H-H}$ = 6.9 Hz, H-6).

^{13}C{^{1}H} NMR (151 MHz, d_2-dichloromethane, 298 K): δ 162.6 (s, C-2), 161.7 (s, C-1), 148.7 (s, Ar), 146.8 (s, Ar), 133.4 (s, Ar), 132.8 (s, Ar), 128.0 (s, C-3/4), 121.6 (s, C-3/4), 120.0 (s, Ar), 119.5 (s, Ar), 117.5 (s, Ar), 34.4 (s, H-5), 24.3 (s, H-6).

ESI-MS (CH_2Cl_2): m/z $[M+H]^+$ 240.1380 (calc. 240.1383).

UV-Vis (CH_2Cl_2): λ_{max}/nm [log(ε dm^3 mol^{-1} cm^{-1})] 232 (4.95), 270 (4.73), 308 (4.69), 322 (4.76), 344 (4.81).

L-d yield (2.45 g, 0.012 mol, 90%).

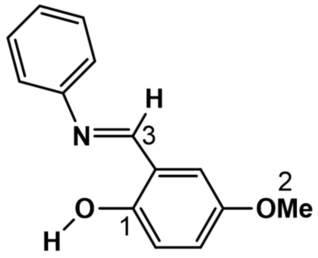

^{1}H NMR (600 MHz, *d*$_2$-dichloromethane, 298 K): δ 12.70 (s, 1H, OH), 8.63 (s, 1H, H-3), 7.44 (t, 2H, $^{3}J_{H\text{-}H}$ = 7.8 Hz), 7.31–7.29 (m, 3H), 7.01–6.99 (m, 1H), 6.94–6.92 (m, 2H), 3.80 (s, 3H, OCH_3).

^{13}C{^{1}H} NMR (151 MHz, *d*$_2$-dichloromethane, 298 K): δ 163.2 (s, C-3), 155.9 (s, C-1), 152.9 (s, Ar), 149.2 (s, Ar), 130.0 (s, Ar), 127.5 (s, Ar), 121.7 (s, Ar), 121.0 (s, Ar), 119.5 (s, Ar), 118.4 (s, Ar), 116.0 (s, Ar), 56.4 (s, C-2).

These NMR data are in accordance with the literature reported data.[7]

ESI-MS (CH_2Cl_2): m/z $[M+H]^+$ 228.1019 (calc. 228.1025).

UV-Vis (CH_2Cl_2): λ_{max}/nm [log(ε dm^3 mol^{-1} cm^{-1})] 240 (4.63), 274 (4.43), 294 (4.39), 308 (4.38), 376 (4.26).

L-e yield (1.56 g, 0.007 mol, 94%).

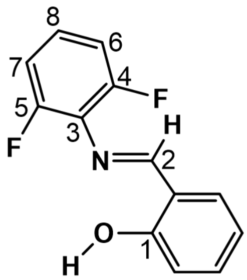

^{1}H NMR (600 MHz, d_2-dichloromethane, 298 K): δ 12.84 (s, 1H, OH), 8.89 (s, 1H, H-2), 7.44–7.71 (m, 2H), 7.21–7.16 (m, 1H), 7.06–7.01 (m, 3H), 6.97 (t, 1H, $^{3}J_{H\text{-}H}$ = 7.6 Hz).

^{13}C{^{1}H} NMR (151 MHz, d_2-dichloromethane, 298 K): δ 169.6 (t, $^{4}J_{C\text{-}F}$ = 5.0 Hz, C-2), 162.0 (s, C-1), 157.4 (d, $^{1}J_{C\text{-}F}$ = 255.5 Hz, C-4), 157.3 (d, $^{1}J_{C\text{-}F}$ = 245.6 Hz, C-5), 134.5 (s, Ar), 133.5 (s, Ar), 127.3 (t, $^{3}J_{C\text{-}F}$ = 10.0 Hz, C-8), 126.0 (t, $^{2}J_{C\text{-}F}$ = 12.9 Hz, C-3), 119.8 (s, Ar), 199.7 (s, Ar), 117.9 (s, Ar), 112.7 (d, $^{2}J_{C\text{-}F}$ = 19.3 Hz, C-6), 112.6 (d, $^{2}J_{C\text{-}F}$ = 19.3 Hz, C-7).

^{19}F NMR (565 MHz, d_2-dichloromethane, 298 K): δ –123.8 (m).

These NMR data are in accordance with the literature reported data.[8]

ESI-MS (CH_2Cl_2): m/z $[M+H]^+$ 234.0719 (calc. 234.0725).

UV-Vis (CH_2Cl_2): λ_{max}/nm [log(ε dm^3 mol^{-1} cm^{-1})] 230 (4.69), 270 (4.57), 300 (4.46), 316 (4.45), 344 (4.45).

L-f yield (3.96 g, 0.014 mol, 92%).

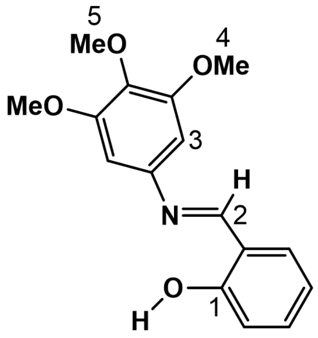

^{1}H NMR (600 MHz, d_2-dichloromethane, 298 K): δ 13.14 (s, 1H, OH), 8.66 (s, 1H, H-2), 7.45 (d, 1H, $^3J_{H-H}$ = 7.6 Hz), 7.38 (t, 1H, $^3J_{H-H}$ = 7.6 Hz), 7.00–7.95 (m, 2H), 6.57 (s, 2H, H-3), 3.88 (s, 6H, 2 x OCH_3), 3.80 (s, 3H, OCH_3).

^{13}C{^{1}H} NMR (151 MHz, d_2-dichloromethane, 298 K): δ 162.5 (s, C-2), 161.6 (s, C-1), 154.5 (s, Ar), 144.8 (s, Ar), 137.9 (s, Ar), 133.6 (s, Ar), 132.9 (s, Ar), 119.6 (s, Ar), 117.6 (s, Ar), 99.1 (s, C-3), 93.0 (s, Ar), 61.1 (s, C-5), 56.7 (s, C-4).

These NMR data are in accordance with the literature reported data.[9]

ESI-MS (CH_2Cl_2): m/z $[M+H]^+$ 288.1231 (calc. 288.1230).

UV-Vis (CH_2Cl_2): λ_{max}/nm [log(ε dm^3 mol^{-1} cm^{-1})] 266 (4.38), 352 (4.51).

1.2.3 Pt(sal)(ppy) complexes

All Pt(sal)(ppy) complexes (**1a** to **1h**) were prepared by the same procedure modified from the literature.[10] In the literature procedure, the Pt(sal)(ppy) complexes were synthesised from the [Pt(*N^C*)(μ-Cl)]$_2$ complexes directly, but herein the [Pt{NC$_5$H$_4$-6-(4'-R-C$_6$H$_3$)}Cl(κ$_1$-S-dmso)] complexes were synthesised first. As an example, for **1f**, a solution of **2-dmso** (340 mg, 0.69 mmol), 3 equiv. of the Schiff base ligand (549 mg, 2.07 mmol) and 10 equiv. of Na_2CO_3 (731 mg, 6.9 mmol) in 2-methoxyethanol (50 mL) were heated to reflux for 20 h under a nitrogen atmosphere. The solvent was then removed under reduced pressure and hexane (15 mL) was added gradually to give an orange precipitate that was subsequently filtered and washed with water (10 mL). Subsequent freeze-drying followed by recrystallisation by slow diffusion of hexane into a dichloromethane solution of the complex resulted in the formation of **1f** in moderate yield (240 mg, 0.37 mmol, 54%).

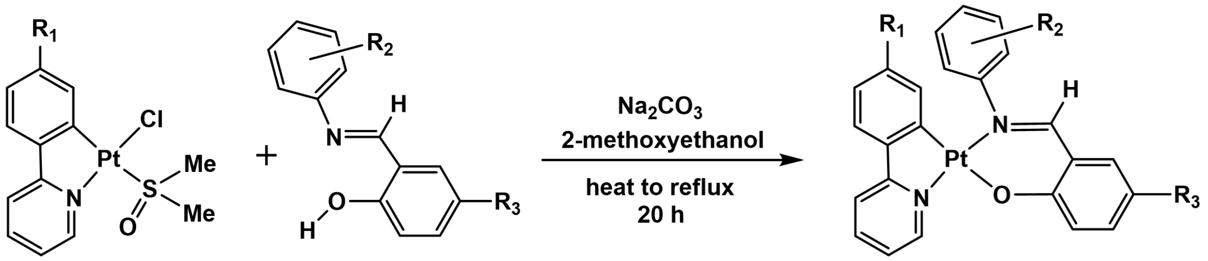

Scheme S3. General synthesis of Pt(sal)(ppy) complexes (**1a–1h**).

1a yield (365 mg, 0.67 mmol, 48%).

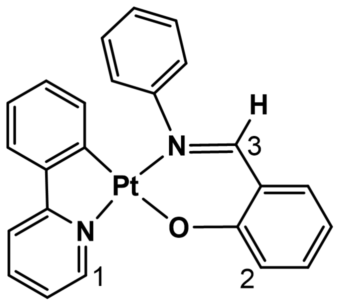

^{1}H NMR (400 MHz, *d*$_2$-dichloromethane, 298 K): δ 9.54 (d, 1H, $^3J_{H-H}$ = 6.0 Hz, $^3J_{H-Pt}$ = 34.9 Hz, H-1), 8.27 (s, 1H, $^3J_{H-Pt}$ = 76.5 Hz, H-3), 7.85 (t, 1H, $^3J_{H-H}$ = 7.8 Hz, ppy), 7.67–7.65 (m, 1H, ppy), 7.58–7.57 (m, 2H, sal), 7.48 (t, 1H, $^3J_{H-H}$ = 7.7 Hz, ppy), 7.42–7.33 (m, 5H, 2 ppy + 3 sal), 7.28 (t, 1H, $^3J_{H-H}$ = 6.7 Hz, ppy), 7.07–7.05 (m, 1H, ppy), 6.85 (t, 1H, $^3J_{H-H}$ = 7.4 Hz, sal), 6.60 (t, 1H, $^3J_{H-H}$ = 7.4 Hz, sal), 6.54 (t, 1H, $^3J_{H-H}$ = 7.5 Hz, sal), 5.68 (d, 1H, $^3J_{H-H}$ = 7.7 Hz, $^4J_{H-Pt}$ = 35.4 Hz, H-2).

^{13}C{^{1}H} NMR (151 MHz, *d*$_2$-dichloromethane, 298 K): δ 168.3 (s, ppy), 166.3 (s, sal), 163.9 (s, C-3), 155.5 (s, sal), 147.0 (s, ppy), 146.2 (s, ppy), 139.4 (s, ppy), 135.9 (s, ppy), 135.1 (s, sal), 129.4 (s, sal), 128.9 (s, sal), 127.8 (s, ppy), 126.2 (s, ppy), 123.4 (s, ppy), 123.1 (s, ppy), 123.0 (s, sal), 122.4 (s, sal), 121.4 (s, ppy), 118.8 (s, ppy), 115.9 (s, sal).

These NMR data are in accordance with the literature reported data.[10]

ESI-MS (CH_2Cl_2): m/z [M+H]$^+$ 546.1146 (calc. 546.1140) with the correct isotope pattern.

UV-Vis (CH_2Cl_2): λ_{max}/nm [log(ε dm^3 mol^{-1} cm^{-1})] 228 (4.33), 266 (4.45), 308 (3.91), 362 (3.89), 398 (3.64).

1b yield (64 mg, 0.104 mmol, 48%).

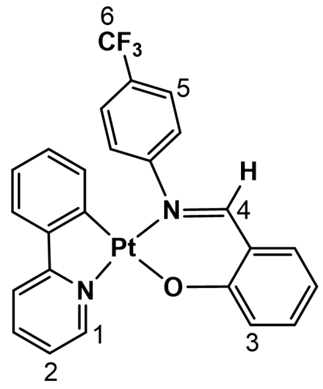

^{1}H NMR (600 MHz, *d_2*-dichloromethane, 298 K): δ 9.51 (d, 1H, $^3J_{H-H}$ = 5.7 Hz, $^3J_{H-Pt}$ = 33.5 Hz, H-1), 8.27 (s, 1H, $^3J_{H-Pt}$ = 70.5 Hz, H-4), 7.86 (1H, t, $^3J_{H-H}$ = 7.7 Hz, ppy), 7.74–7.73 (m, 2H, sal), 7.69–7.66 (m, 3H, 1 ppy + 2 sal), 7.51 (t, 1H, $^3J_{H-H}$ = 7.7 Hz, ppy), 7.41 (d, 1H, $^3J_{H-H}$ = 7.7 Hz, ppy), 7.37 (d, 1H, $^3J_{H-H}$ = 7.7 Hz, sal), 7.29 (t, 1H, $^3J_{H-H}$ = 5.7 Hz, H-2), 7.07 (m, 1H, ppy), 6.87 (t, 1H, $^3J_{H-H}$ = 7.4 Hz, sal), 6.62 (t, 1H, $^3J_{H-H}$ = 7.4 Hz, sal), 6.55 (t, 1H, $^3J_{H-H}$ = 7.4 Hz, sal), 5.63 (d, 1H, $^3J_{H-H}$ = 7.8 Hz, $^4J_{H-Pt}$ = 35.2 Hz, H-3).

^{13}C{^{1}H} NMR (151 MHz, *d_2*-dichloromethane, 298 K): δ 176.0 (s, ppy), 168.2 (s, ppy), 166.7 (s, C-4), 164.1 (s, ppy), 152.3 (s, ppy), 158.3 (s, sal), 147.0 (s, ppy), 146.3 (s, sal), 139.6 (s, ppy), 139.1 (s, ppy), 136.5 (s, ppy), 135.2 (s, ppy), 135.0 (s, sal), 129.8 (s, ppy), 129.6 (s, ppy), 129.0 (s, sal), 126.6 (q, $^3J_{C-F}$ = 3.7 Hz, C-5), 122.6 (q, $^1J_{C-F}$ = 313.8 Hz, C-6), 123.3 (s, sal), 122.3 (s, sal), 119.4 (s, sal), 118.9 (s, sal), 116.2 (s, sal).

^{19}F NMR (565 MHz, *d_2*-dichloromethane, 298 K): δ –62.5 (s).

These NMR data are in accordance with the literature reported data.[10]

ESI-MS (CH_2Cl_2): m/z $[M+H]^+$ 614.1005 (calc. 614.0943) with the correct isotope pattern.

UV-Vis (CH_2Cl_2): λ_{max}/nm [log(ε dm^3 mol^{-1} cm^{-1})] 228 (4.55), 264 (4.69), 362 (4.13), 396 (3.86).

1c yield (154 mg, 0.26 mmol, 27%).

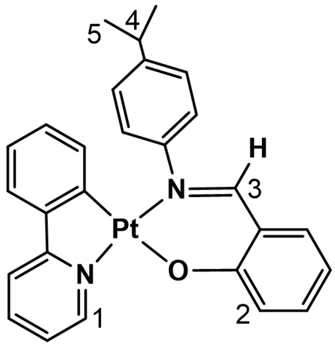

^{1}H NMR (500 MHz, *d_2*-dichloromethane, 298 K): δ 9.54 (d, 1H, $^3J_{H-H}$ = 6.1 Hz, $^3J_{H-Pt}$ = 33.5 Hz, H-1), 8.27 (s, 1H, $^3J_{H-Pt}$ = 73.8 Hz, H-3), 7.85 (t, 1H, $^3J_{H-H}$ = 7.7 Hz, ppy), 7.66 (m, 1H, ppy), 7.48 (m, 3H, 1 ppy + 2 sal), 7.39 (dd, 1H, J_{H-H} = 7.7 and 1.4 Hz, ppy), 7.36 (dd, 1H, J_{H-H} = 7.9 and 1.7 Hz, ppy), 7.28 (m, 3H, 1 ppy + 2 sal), 7.07 (m, 1H, ppy), 6.85 (t, 1H, $^3J_{H-H}$ = 7.4 Hz, sal), 6.59 (t, 1H, $^3J_{H-H}$ = 7.4 Hz, sal), 6.52 (t, 1H, $^3J_{H-H}$ = 7.7 Hz, sal), 5.60 (d, 1H, $^3J_{H-H}$ = 8.1 Hz, $^4J_{H-Pt}$ = 38.2 Hz, H-2), 2.99 (sept, 1H, $^3J_{H-H}$ = 6.9 Hz, H-4), 1.30 (d, 6H, $^3J_{H-H}$ = 6.9 Hz, H-5).

^{13}C{^{1}H} NMR (126 MHz, *d_2*-dichloromethane, 298 K): δ 168.3 (s, ppy), 166.2 (s, ppy), 163.5 (s, C-3), 153.3 (s, sal), 149.1 (s, sal), 146.9 (s, ppy) 146.2 (s, ppy), 139.5 (s, ppy), 139.4 (s, ppy) 135.8 (s, sal), 135.2 (s, sal), 135.0 (s, sal), 128.7 (s, sal), 127.2 (s, ppy), 125.9 (s, sal), 123.3 (s, ppy), 123.1 (s, ppy), 122.9 (s, ppy), 122.4 (s, sal), 121.4 (s, sal), 118.8 (s, ppy), 115.8 (s, sal), 34.4 (s, C-4), 24.5 (s, C-5).

ESI-MS (CH_2Cl_2): m/z $[M+H]^+$ 588.1608 (calc. 588.1609) with the correct isotope pattern.

The sample is pure to the detection limit of NMR spectroscopy.

UV-Vis (CH_2Cl_2): λ_{max}/nm [log(ε dm^3 mol^{-1} cm^{-1})] 266 (4.54), 310 (4.02), 362 (3.99), 398 (3.76).

1d yield (82.5 mg, 0.143 mmol, 44%).

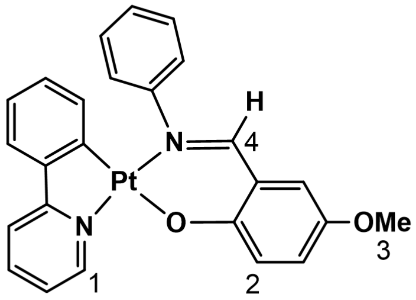

^{1}H NMR (600 MHz, *d_2*-dichloromethane, 298 K): δ 9.52 (d, 1H, $^3J_{H\text{-}H}$ = 5.8 Hz, $^3J_{H\text{-}Pt}$ = 33.2 Hz, H-1), 8.23 (s, 1H, $^3J_{H\text{-}Pt}$ = 74.6 Hz, H-4), 7.84 (t, 1H, $^3J_{H\text{-}H}$ = 7.8 Hz, ppy), 7.66 (d, 1H, $^3J_{H\text{-}H}$ = 7.9 Hz, ppy), 7.58–7.56 (m, 2H, ppy), 7.42–7.38 (m, 3H, 2 ppy + 1 sal), 7.34–7.32 (m, 1H, sal), 7.27 (t, 1H, $^3J_{H\text{-}H}$ = 6.7 Hz, ppy), 7.19–7.16 (m, 1H, sal), 7.03–7.00 (m, 1H, sal), 6.85 (t, 1H, $^3J_{H\text{-}H}$ = 7.5 Hz, sal), 6.77 (1H, d, $^3J_{H\text{-}H}$ = 3.2 Hz, sal), 6.53 (t, 1H, $^3J_{H\text{-}H}$ = 7.5 Hz, sal), 5.68 (d, 1H, $^3J_{H\text{-}H}$ = 8.0 Hz, $^4J_{H\text{-}Pt}$ = 37.5 Hz, H-2), 3.75 (s, 3H, H-3).

^{13}C{^{1}H} NMR (151 MHz, *d_2*-dichloromethane, 298 K): δ 168.3 (s, ppy), 163.1 (s, C-4) 162.0 (s, sal), 155.6 (s, sal), 150.2 (s, sal), 146.9 (s, ppy), 146.2 (s, ppy), 139.8 (s, sal), 139.4 (s, ppy), 135.1 (s, ppy), 129.3 (s, sal), 128.8 (s, sal), 127.7 (s, ppy), 126.8 (s, sal), 126.3 (s, ppy), 124.1 (s, sal), 123.4 (s, sal), 123.0 (s, ppy), 121.4 (s, ppy), 120.6 (s, ppy), 118.8 (s, ppy), 113.8 (s, sal), 56.4 (s, C-3).

ESI-MS (CH_2Cl_2): m/z $[M+H]^+$ 576.1254 (calc. 576.1245) with the correct isotope pattern.

Elemental analysis found (calc. for $C_{25}H_{20}N_2O_2Pt$): C 52.50 (52.17) H 3.12 (3.50) N 4.81 (4.87).

UV-Vis (CH_2Cl_2): λ_{max}/nm [log(ε dm^3 mol^{-1} cm^{-1})] 230 (4.68), 266 (4.77), 310 (4.25), 366 (4.22), 408 (4.01), 476 (3.86).

1e yield (42 mg, 0.073 mmol, 38%).

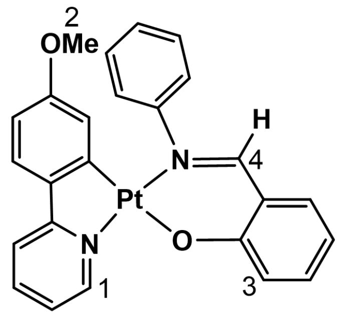

^{1}H NMR (600 MHz, d_2-dichloromethane, 298 K): δ 9.44 (d, 1H, $^3J_{H\text{-}H}$ = 5.7 Hz, $^3J_{H\text{-}Pt}$ = 33.2 Hz, H-1), 8.25 (s, 1H, $^3J_{H\text{-}Pt}$ = 70.7 Hz, H-4), 7.78 (t, 1H, $^3J_{H\text{-}H}$ = 7.5 Hz, ppy), 7.66 (d, 2H, $^3J_{H\text{-}H}$ = 7.6 Hz, sal), 7.53 (d, 1H, $^3J_{H\text{-}H}$ = 7.9 Hz, ppy), 7.47 (t, 1H, $^3J_{H\text{-}H}$ = 7.5 Hz, ppy), 7.42 (t, 2H, $^3J_{H\text{-}H}$ = 7.3 Hz, sal), 7.35–7.30 (m, 3H, 1 ppy + 2 sal), 7.18 (1H, t, $^3J_{H\text{-}H}$ = 6.3 Hz, ppy), 7.06 (d, 1H, $^3J_{H\text{-}H}$ = 8.6 Hz, ppy), 6.60 (t, 1H, $^3J_{H\text{-}H}$ = 7.3 Hz, sal), 6.43 (1H, d, $^3J_{H\text{-}H}$ = 8.3 Hz, sal), 5.42 (s, 1H, H-3), 3.24 (s, 3H, H-2).

^{13}C{^{1}H} NMR (151 MHz, d_2-dichloromethane, 298 K): δ 168.1 (s, sal), 166.4 (s, C-4), 164.4 (s, ppy), 160.2 (s, ppy), 155.4 (s, sal), 146.6 (s, ppy), 141.7 (s, ppy), 139.3 (s, ppy), 138.8 (s, ppy), 136.0 (s, ppy), 135.1 (s, sal), 129.6 (s, sal), 128.0 (s, ppy), 126.2 (s, ppy), 124.8 (s, ppy), 123.0 (s, sal), 122.5 (s, ppy), 120.1 (s, sal), 118.5 (s, sal), 118.1 (s, sal), 116.0 (s, sal), 111.0 (s, sal), 55.5 (s, C-2).

ESI-MS (CH_2Cl_2): m/z $[M+H]^+$ 576.1251 (calc. 576.1245) with the correct isotope pattern.

Elemental analysis found (calc. for $C_{25}H_{20}N_2O_2Pt$): C 52.44 (52.17) H 3.21 (3.50) N 4.80 (4.87).

UV-Vis (CH_2Cl_2): λ_{max}/nm [log(ε dm^3 mol^{-1} cm^{-1})] 230 (4.51), 270 (4.63), 360 (4.18), 394 (3.96).

1f yield (240 mg, 0.37 mmol, 54%).

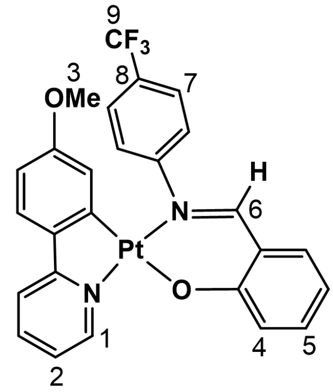

^{1}H NMR (600 MHz, *d_2*-dichloromethane, 298 K): δ 9.42 (d, 1H, $^3J_{H\text{-}H}$ = 5.8 Hz, $^3J_{H\text{-}Pt}$ = 33.2 Hz, H-1), 8.25 (s, 1H, $^3J_{H\text{-}Pt}$ = 70.6 Hz, H-6), 7.81–7.78 (m, 3H, ppy), 7.71–7.69 (m, 2H, sal), 7.53–7.49 (m, 2H, sal), 7.36 (d, 1H, $^3J_{H\text{-}H}$ = 7.9 Hz, ppy), 7.32 (d, 1H, $^3J_{H\text{-}H}$ = 8.5 Hz, ppy), 7.18 (t, 1H, $^3J_{H\text{-}H}$ = 6.7 Hz, H-2), 7.06 (d, 1H, $^3J_{H\text{-}H}$ = 8.6 Hz, sal), 6.61 (t, 1H, $^3J_{H\text{-}H}$ = 7.4 Hz, sal), 6.44 (dd, 1H, $^3J_{H\text{-}H}$ = 8.5 and 2.5 Hz, H-5), 5.30 (d, 1H, $^3J_{H\text{-}H}$ = 2.5 Hz, H-4), 3.23 (s, 3H, H-3).

^{13}C{^{1}H} NMR (151 MHz, *d_2*-dichloromethane, 298 K): δ 168.0 (s, ppy), 166.8 (s, sal), 164.5 (s, C-6), 160.3 (s, ppy), 158.2 (s, ppy), 146.6 (s, ppy), 141.3 (s, ppy), 139.4 (s, ppy), 138.9 (s, ppy), 136.6 (s, sal), 135.2 (s, sal), 129.9 (q, $^2J_{C\text{-}F}$ = 32.3 Hz, C-8), 126.9 (s, sal), 126.8 (q, $^3J_{C\text{-}F}$ = 3.8 Hz, C-7), 124.6 (q, $^1J_{C\text{-}F}$ = 273.1 Hz, C-9), 124.9 (s, ppy), 123.3 (s, ppy), 122.3 (s, sal), 120.2 (s, ppy), 118.7 (s, ppy), 118.2 (s, sal), 116.2 (s, sal), 111.0 (s, sal), 55.2 (s, C-3).

^{19}F NMR (565 MHz, *d_2*-dichloromethane, 298 K): δ –62.6 (s).

ESI-MS (CH_2Cl_2): m/z $[M+H]^+$ 644.1127 (calc. 644.1119) with the correct isotope pattern.

Elemental analysis found (calc. for $C_{26}H_{19}F_3N_2O_2Pt$): C 48.77 (48.53) H 3.30 (2.98) N 4.05 (4.35).

UV-Vis (CH_2Cl_2): λ_{max}/nm [log(ε dm^3 mol^{-1} cm^{-1})] 228 (4.66), 268 (4.75), 362 (4.29), 394 (4.07).

1g yield (136 mg, 0.23 mmol, 54%).

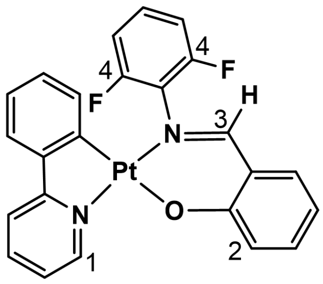

^{1}H NMR (600 MHz, *d_2*-dichloromethane, 298 K): δ 9.55 (d, 1H, $^{3}J_{H\text{-}H}$ = 5.8 Hz, $^{3}J_{H\text{-}Pt}$ = 35.5 Hz, H-1), 8.19 (s, 1H, $^{3}J_{H\text{-}Pt}$ = 73.1 Hz, H-3), 7.84 (t, 1H, $^{3}J_{H\text{-}H}$ = 7.7 Hz, ppy), 7.66–7.65 (m, 1H, ppy), 7.56 (t, 1H, $^{3}J_{H\text{-}H}$ = 7.7 Hz, sal), 7.45 (d, 1H, $^{3}J_{H\text{-}H}$ = 7.7 Hz, ppy), 7.36–7.31 (m, 2H, sal), 7.27 (t, 1H, $^{3}J_{H\text{-}H}$ = 6.6 Hz, ppy), 7.11–7.07 (m, 3H, ppy), 6.92 (t, 1H, $^{3}J_{H\text{-}H}$ = 7.4 Hz, sal), 6.65–6.59 (m, 2H, sal), 5.60 (d, 1H, $^{3}J_{H\text{-}H}$ = 7.9 Hz, $^{4}J_{H\text{-}Pt}$ = 37.2 Hz, H-2).

^{13}C{^{1}H} NMR (151 MHz, *d_2*-dichloromethane, 298 K): δ 168.1 (s, C-3), 166.7 (d, $^{3}J_{C\text{-}F}$ = 4.8 Hz, sal), 158.2–156.6 (dd, 2C, $^{1}J_{C\text{-}F}$ = 249.9 and $^{3}J_{C\text{-}F}$ = 3.5 Hz, C-4), 146.9 (s, ppy), 146.7 (s, ppy), 139.4 (s, ppy), 137.6 (s, ppy), 136.8 (s, ppy), 135.2 (s, sal), 132.2 (s, sal), 129.0 (s, ppy), 128.6 (t, $^{2}J_{C\text{-}F}$ = 9.6 Hz, sal), 123.9 (s, ppy), 123.6 (s, ppy), 123.5 (s, sal) 122.0 (s, sal), 121.5 (s, sal), 118.8 (s, ppy), 116.2 (s, ppy), 112.9 (d, $^{2}J_{C\text{-}F}$ = 19.7 Hz, sal), 112.7 (d, $^{2}J_{C\text{-}F}$ = 19.7 Hz, sal).

^{19}F NMR (565 MHz, *d_2*-dichloromethane, 298 K): δ –120.1 (dd).

ESI-MS (CH_2Cl_2): m/z $[M+H]^+$ 582.0943 (calc. 582.0951) with the correct isotope pattern.

Elemental analysis found (calc. for $C_{24}H_{16}F_2N_2OPt$): C 49.96 (49.57) H 3.09 (2.77) N 4.69 (4.82).

UV-Vis (CH_2Cl_2): λ_{max}/nm [log(ε dm^3 mol^{-1} cm^{-1})] 230 (4.54), 260 (4.67), 300 (4.17), 362 (4.19).

1h yield (109 mg, 0.17 mmol, 36%).

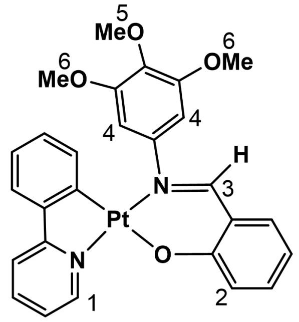

^{1}H NMR (600 MHz, *d_2*-dichloromethane, 298 K): δ 9.58 (d, 1H, $^3J_{H\text{-}H}$ = 5.7 Hz, $^3J_{H\text{-}Pt}$ = 33.7 Hz, H-1), 8.35 (s, 1H, $^3J_{H\text{-}Pt}$ = 71.4 Hz, H-3), 7.85 (t, 1H, $^3J_{H\text{-}H}$ = 7.7 Hz, ppy), 7.67–7.66 (m, 1H, ppy), 7.49 (t, 1H, $^3J_{H\text{-}H}$ = 7.7 Hz, ppy), 7.40 (dd, 1H, $J_{H\text{-}H}$ = 7.6 and 1.2 Hz, ppy), 7.37 (dd, 1H, $J_{H\text{-}H}$ = 7.9 and 1.6 Hz, ppy), 7.27 (t, 1H, $^3J_{H\text{-}H}$ = 6.7 Hz, ppy), 7.06 (d, 1H, $^3J_{H\text{-}H}$ = 8.4 Hz, ppy), 6.89 (t, 1H, $^3J_{H\text{-}H}$ = 7.4 Hz, sal), 6.83 (s, 2H, H-4), 6.65 (t, 1H, $^3J_{H\text{-}H}$ = 7.6 Hz, sal), 6.61 (t, 1H, $^3J_{H\text{-}H}$ = 7.4 Hz, sal), 5.79 (d, 1H, $^3J_{H\text{-}H}$ = 7.9 Hz, H-2), 3.81 (s, 3H, H-5), 3.76 (s, 6H, H-6).

^{13}C{^{1}H} NMR (151 MHz, *d_2*-dichloromethane, 298 K): δ 168.4 (s, ppy), 166.3 (s, ppy), 163.2 (s, C-3), 153.7 (s, sal), 151.2 (s, sal), 146.9 (s, sal), 139.4 (s, ppy), 138.2 (s, ppy), 135.9 (s, ppy), 135.0 (s, sal), 128.9 (s, sal), 123.3 (s, ppy), 123.1 (s, ppy), 123.0 (s, ppy), 122.2 (s, sal), 121.3 (s, sal), 119.9 (s, ppy), 118.8 (s, ppy), 118.1 (s, ppy), 115.9 (s, sal), 104.3 (s, sal), 93.0 (s, sal), 61.5 (s, C-5), 56.9 (s, 2 x C-6).

ESI-MS (CH_2Cl_2): m/z $[M+H]^+$ 636.1468 (calc. 636.1457) with the correct isotope pattern.

The sample is pure to the detection limit of NMR spectroscopy.

UV-Vis (CH_2Cl_2): λ_{max}/nm [log(ε dm^3 mol^{-1} cm^{-1})] 228 (4.45), 264 (4.44), 310 (3.94), 362 (3.92), 396 (3.67).

1.2.4 Synthesis of d_1-hexamethylsiloxymethylsilane

Deuterated hexamethylsiloxymethylsilane was prepared in accordance with the literature procedure.[11] To a solution of hexamethylsiloxymethylsilane (8.0 g, 36 mmol) in THF (10 mL) was added $Rh(PPh_3)_3Cl$ (190 mg, 0.19 mmol). The mixture was degassed by three successive freeze-pump-thaw (FPT) cycles and then charged with D_2 (3 bar) and stirred vigorously at 25 °C. The FPT process was repeated after 2 and 4 h to remove excess HD and the solution was each time recharged with D_2 (3 bar). The product was obtained as a colorless liquid via distillation in good yield (6.0 g, 27 mmol, 75%). The ^1H and ^2H NMR data indicate 99.8% D-incorporation.

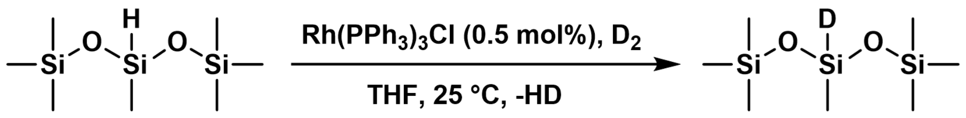

Scheme S4. Synthesis of d_1-hexamethylsiloxymethylsilane.

2 NMR spectra

2.1 NMR spectra of [Pt{NC_5H_4-6-(4'-R-C_6H_3)}Cl(κ_1-S-dmso)] complexes

2.1.1 1-dmso

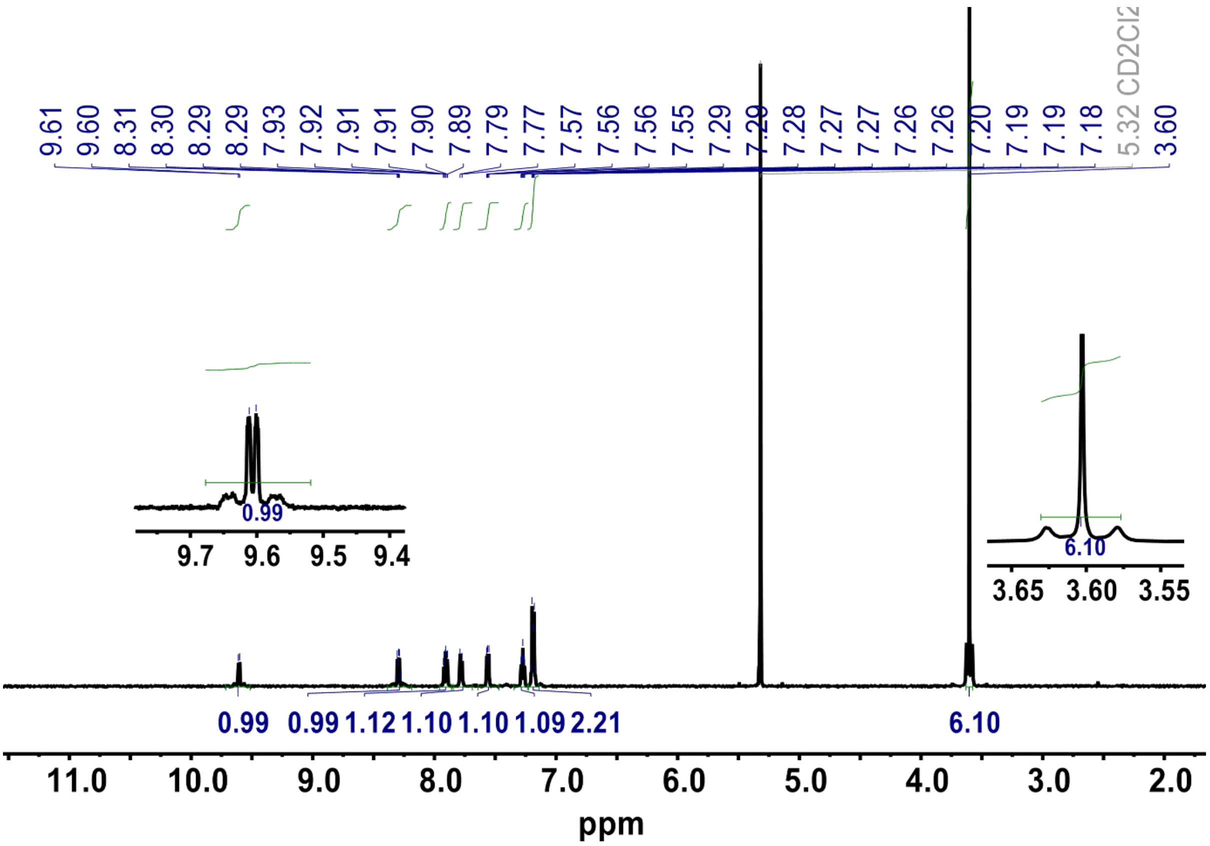

Figure S1. ^{1}H NMR spectrum of **1-dmso** with insets to show ^{195}Pt satellites (600 MHz, CD_2Cl_2, 298 K).

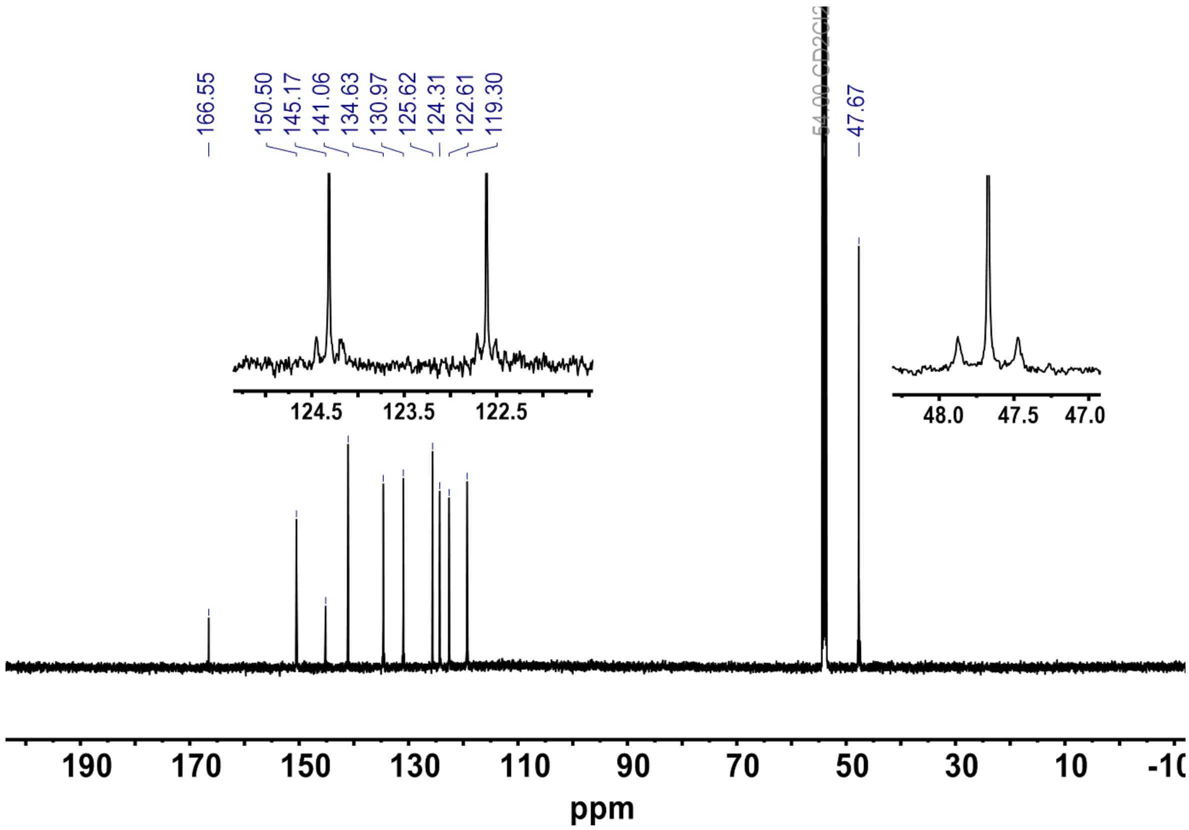

Figure S2. $^{13}C\{^{1}H\}$ NMR spectrum of **1-dmso** with insets to show ^{195}Pt satellites (151 MHz, CD_2Cl_2, 298 K).

2.1.2 2-dmso

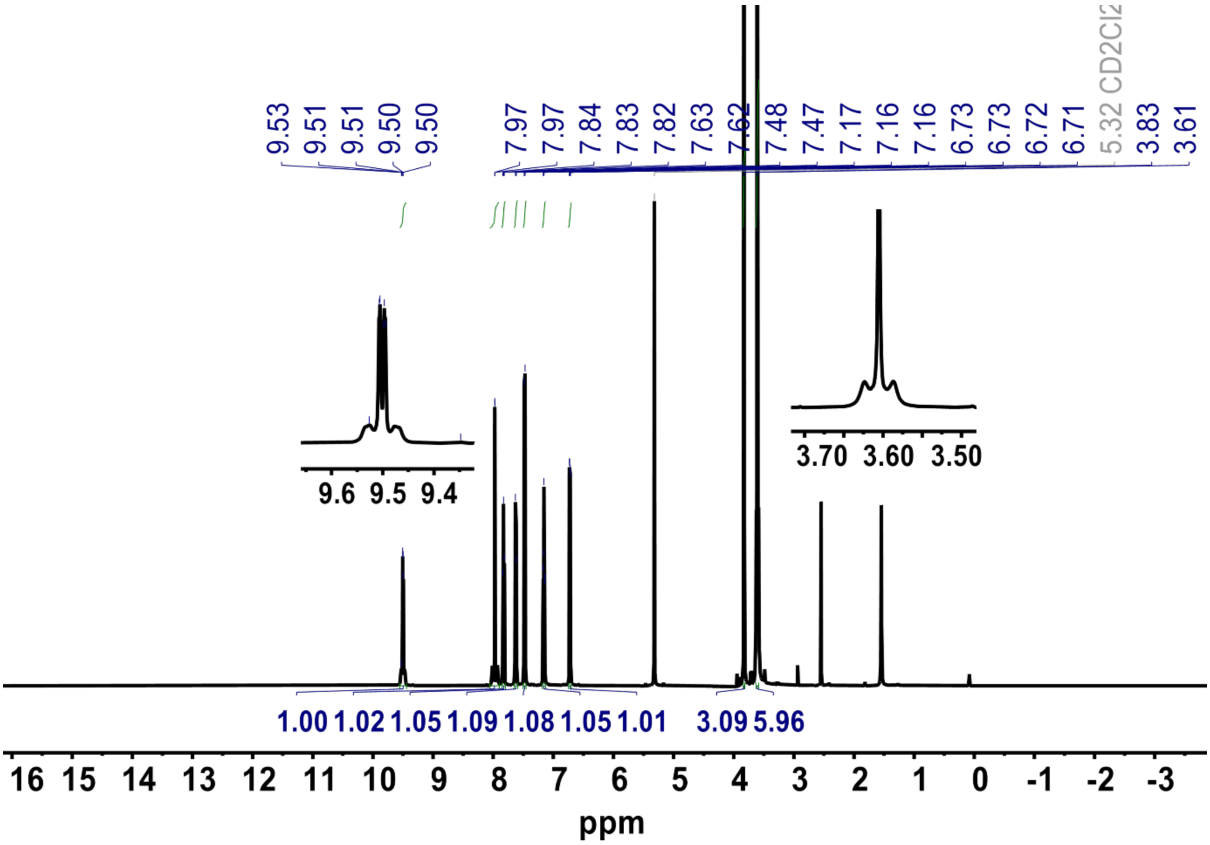

Figure S3. ^{1}H NMR spectrum of **2-dmso** with insets to show ^{195}Pt satellites (600 MHz, CD_2Cl_2, 298K).

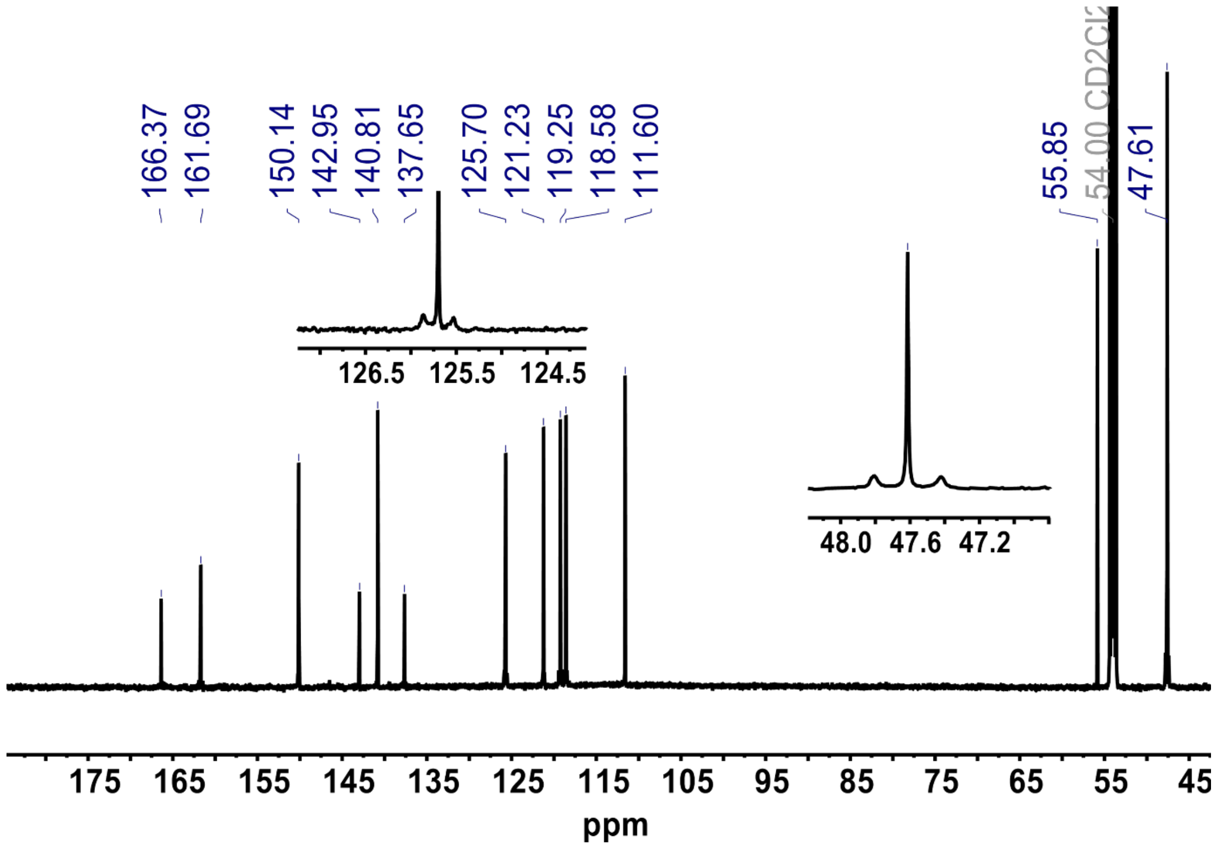

Figure S4. $^{13}C\{^1H\}$ NMR spectrum of **2-dmso** with insets to show ^{195}Pt satellites (151 MHz, CD_2Cl_2, 298 K).

2.2 NMR spectra of Pt(sal)(ppy) complexes

2.2.1 NMR spectra of 1a

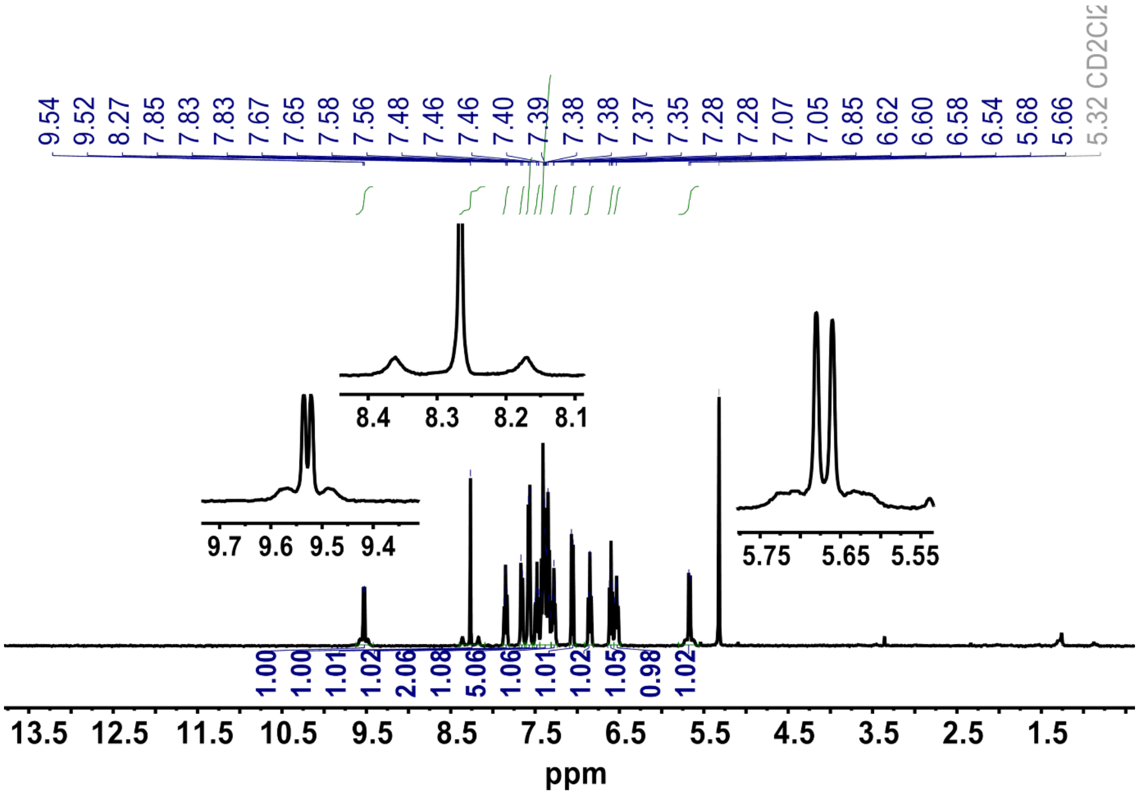

Figure S5. ^{1}H NMR spectrum of **1a** with insets to show ^{195}Pt satellites (600 MHz, CD_2Cl_2, 298K).

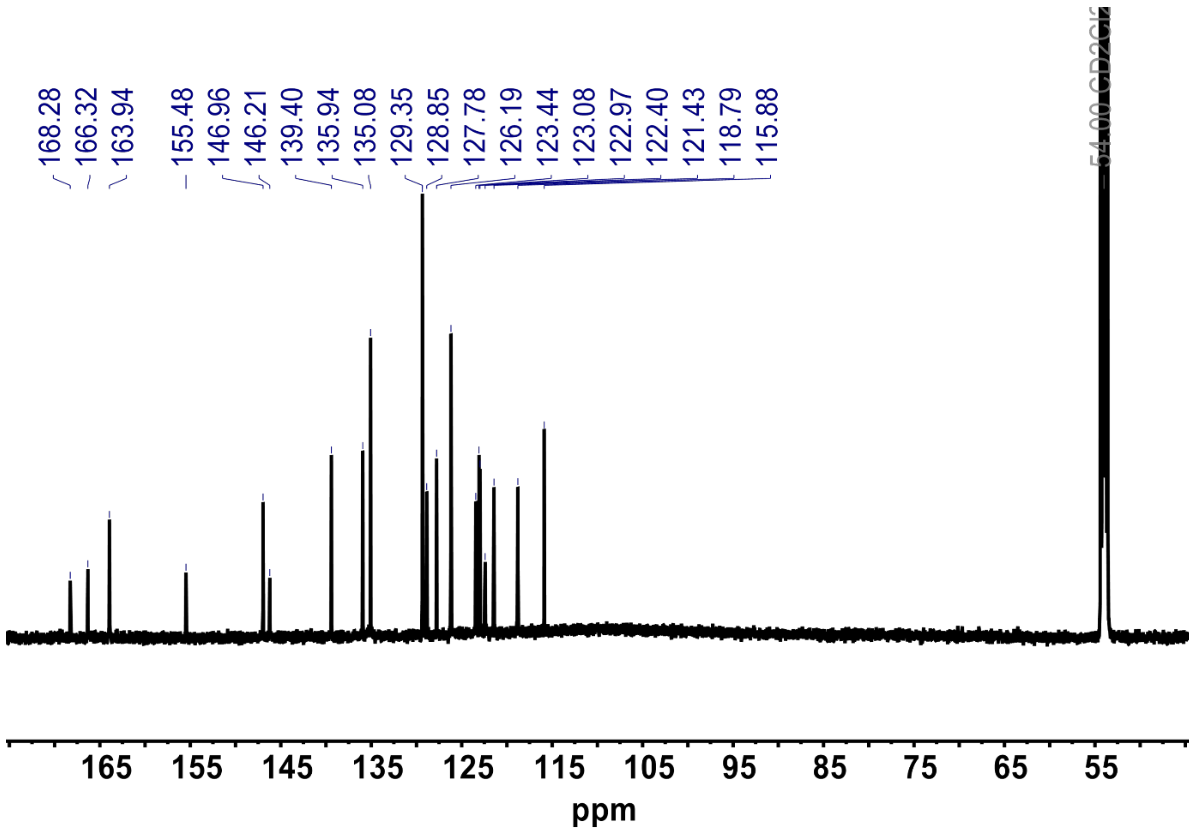

Figure S6. $^{13}C\{^{1}H\}$ NMR spectrum of **1a** (151 MHz, CD_2Cl_2, 298 K).

2.2.2 NMR spectra of 1b

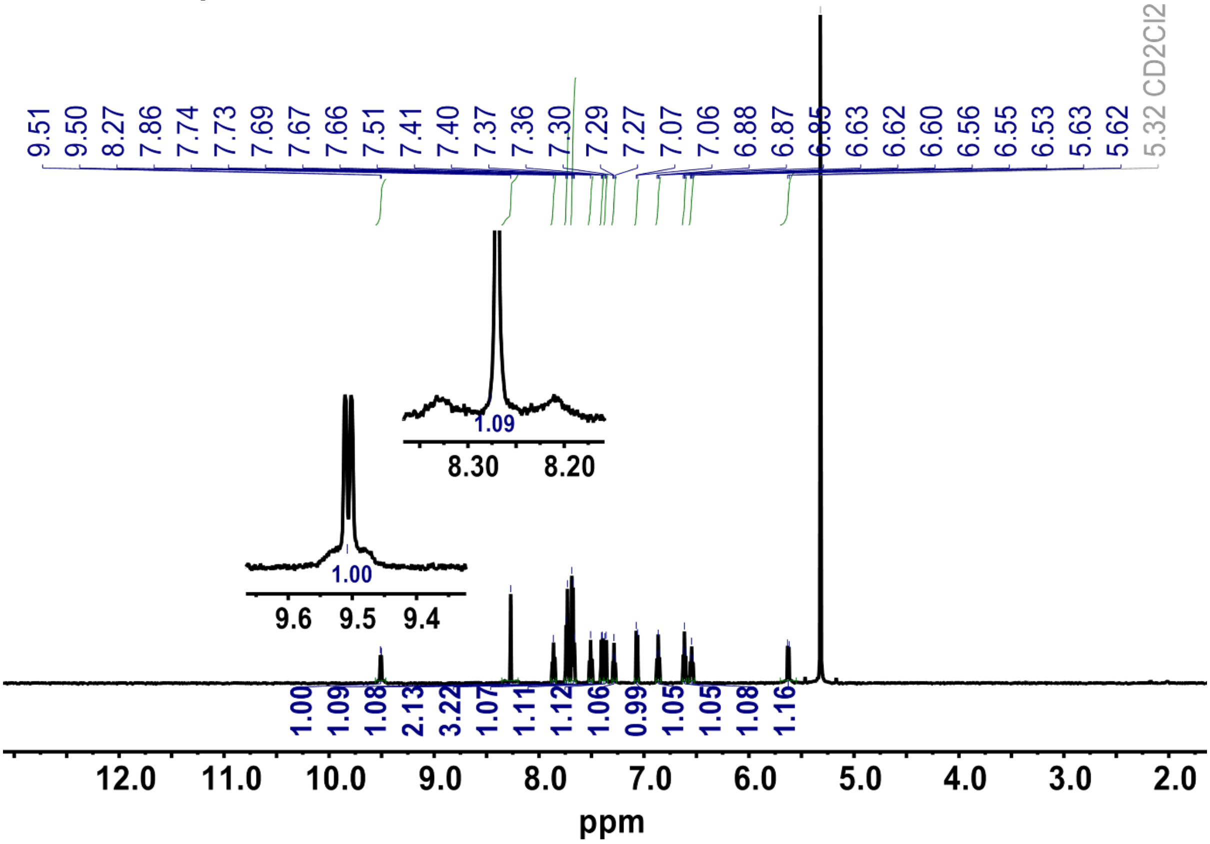

Figure S7. ^{1}H NMR spectrum of **1b** with insets to show ^{195}Pt satellites (600 MHz, CD_2Cl_2, 298K).

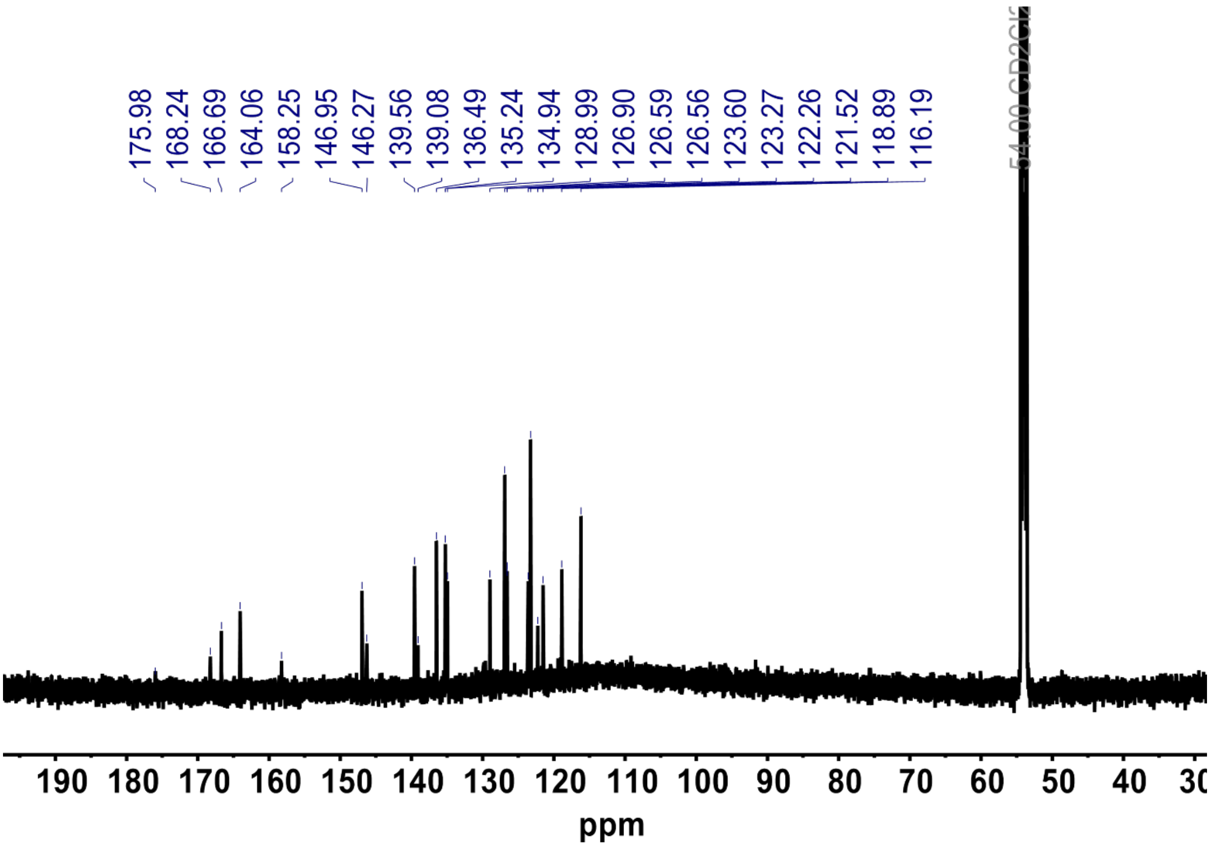

Figure S8. $^{13}C\{^{1}H\}$ NMR spectrum of **1b** (151 MHz, CD_2Cl_2, 298 K).

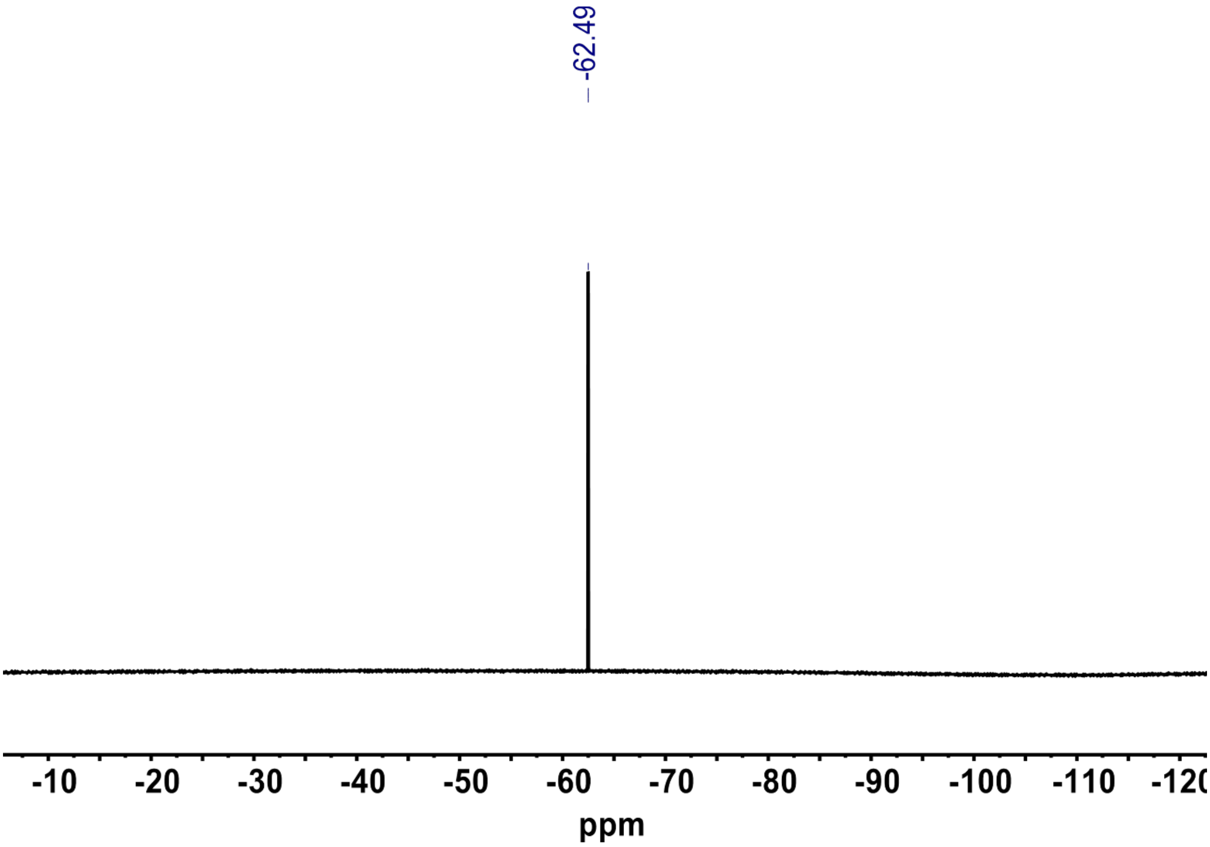

Figure S9. $^{19}F\{^{1}H\}$ NMR spectrum of **1b** (565 MHz, CD_2Cl_2, 298 K).

2.2.3 NMR spectra of 1c

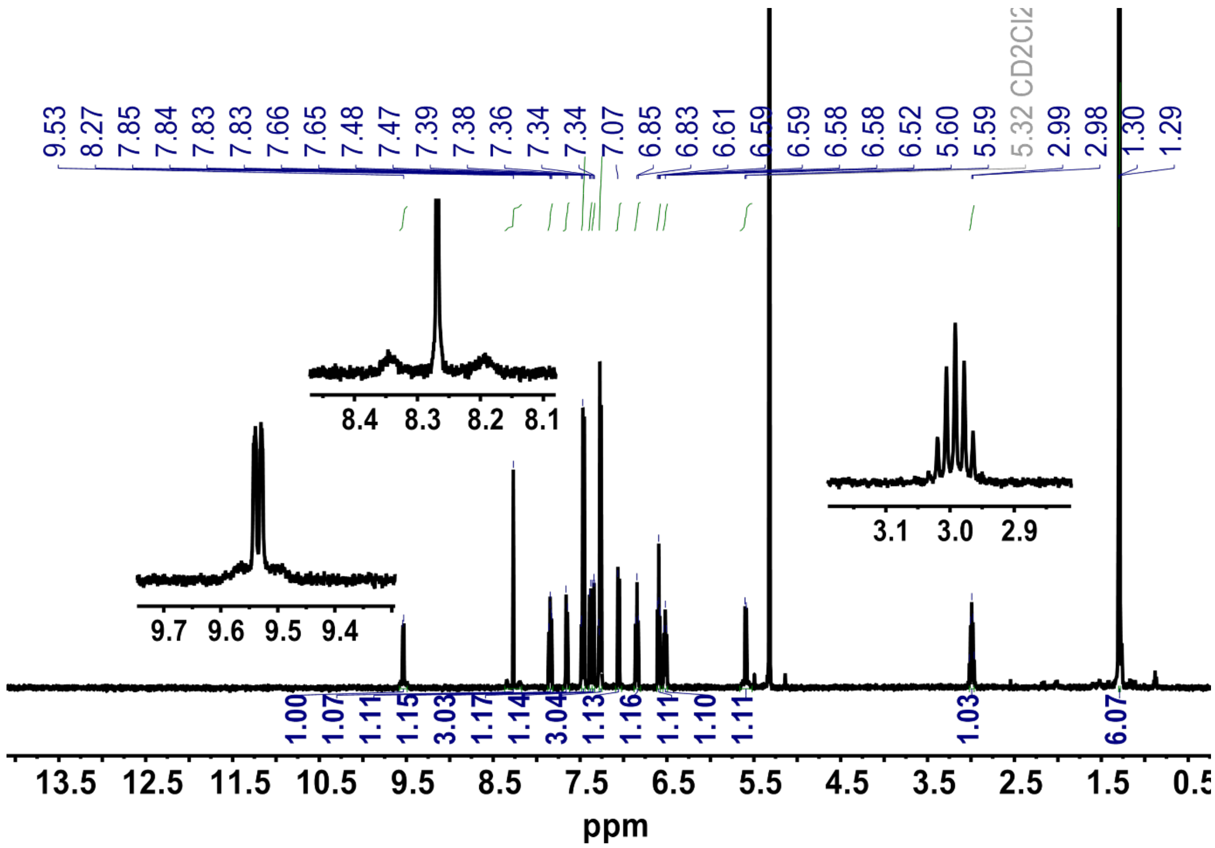

Figure S10. ^{1}H NMR spectrum of **1c** with insets to show ^{195}Pt satellites and multiplicity (600 MHz, CD_2Cl_2, 298K).

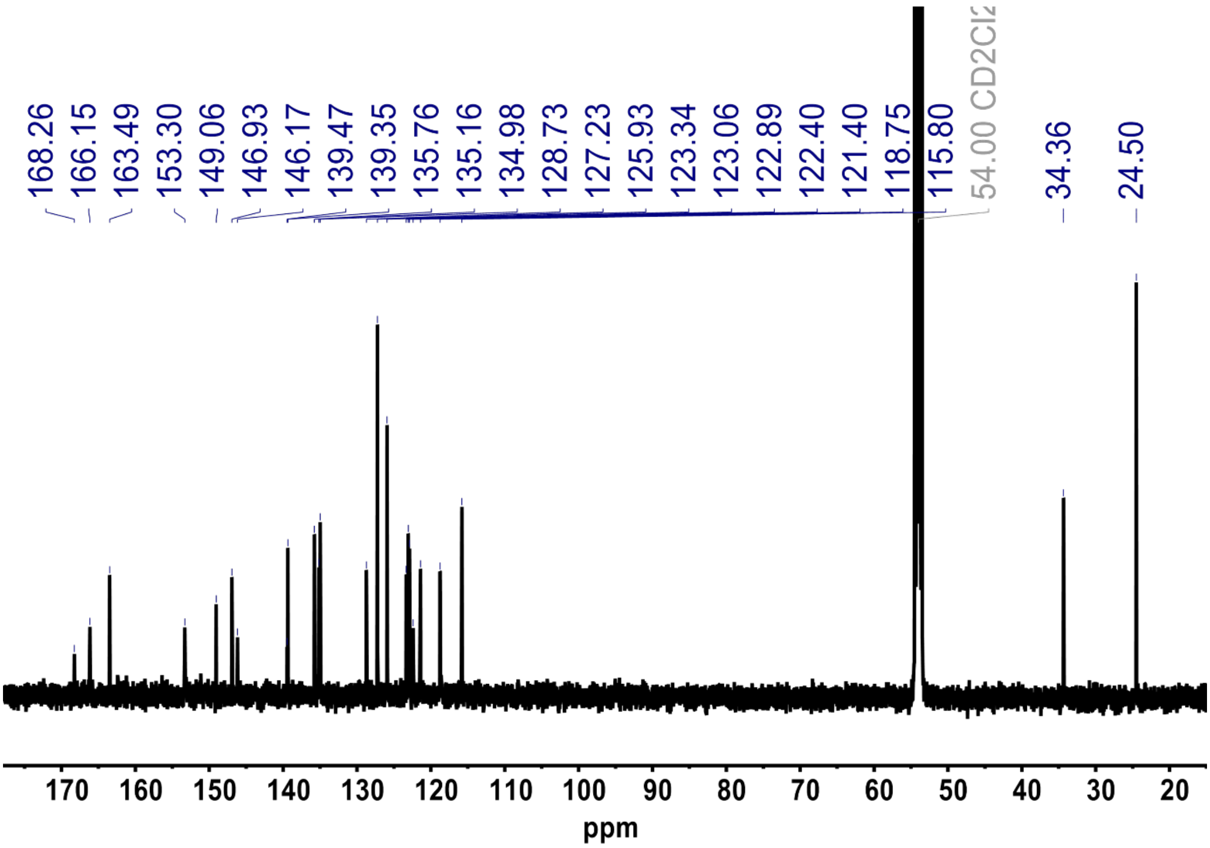

Figure S11. $^{13}C\{^{1}H\}$ NMR spectrum of **1c** (151 MHz, CD_2Cl_2, 298 K).

2.2.4 NMR spectra of 1d

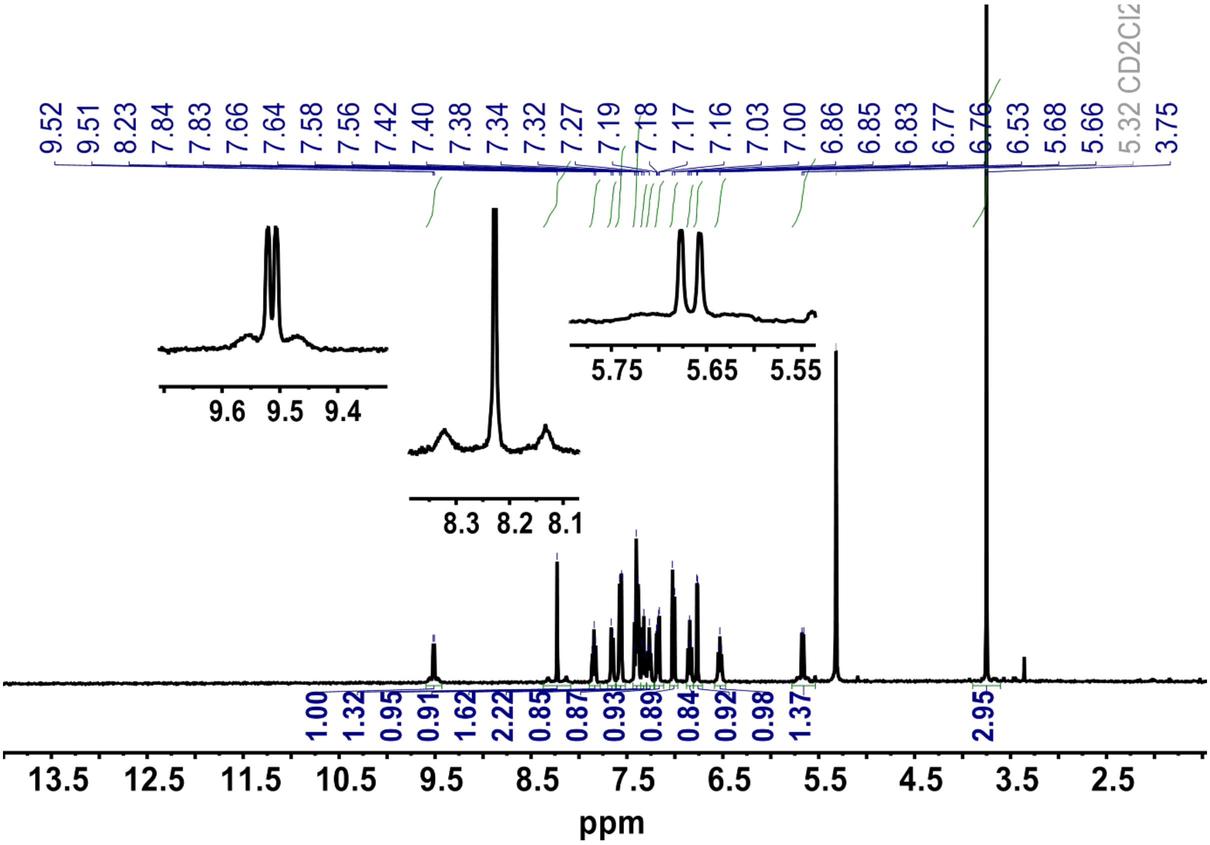

Figure S12. ^{1}H NMR spectrum of **1d** with insets to show ^{195}Pt satellites and multiplicity (600 MHz, CD_2Cl_2, 298K).

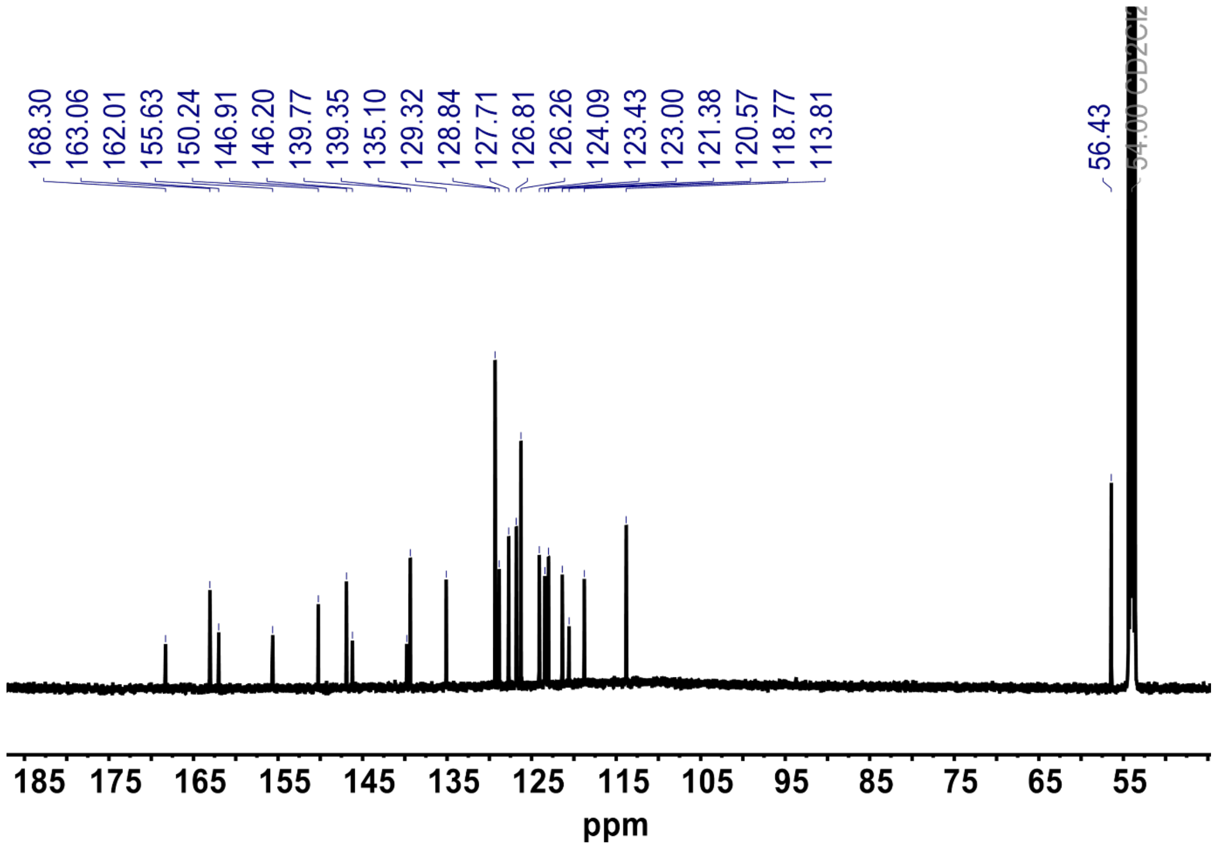

Figure S13. $^{13}C\{^1H\}$ NMR spectrum of **1d** (151 MHz, CD_2Cl_2, 298 K).

2.2.5 NMR spectra of 1e

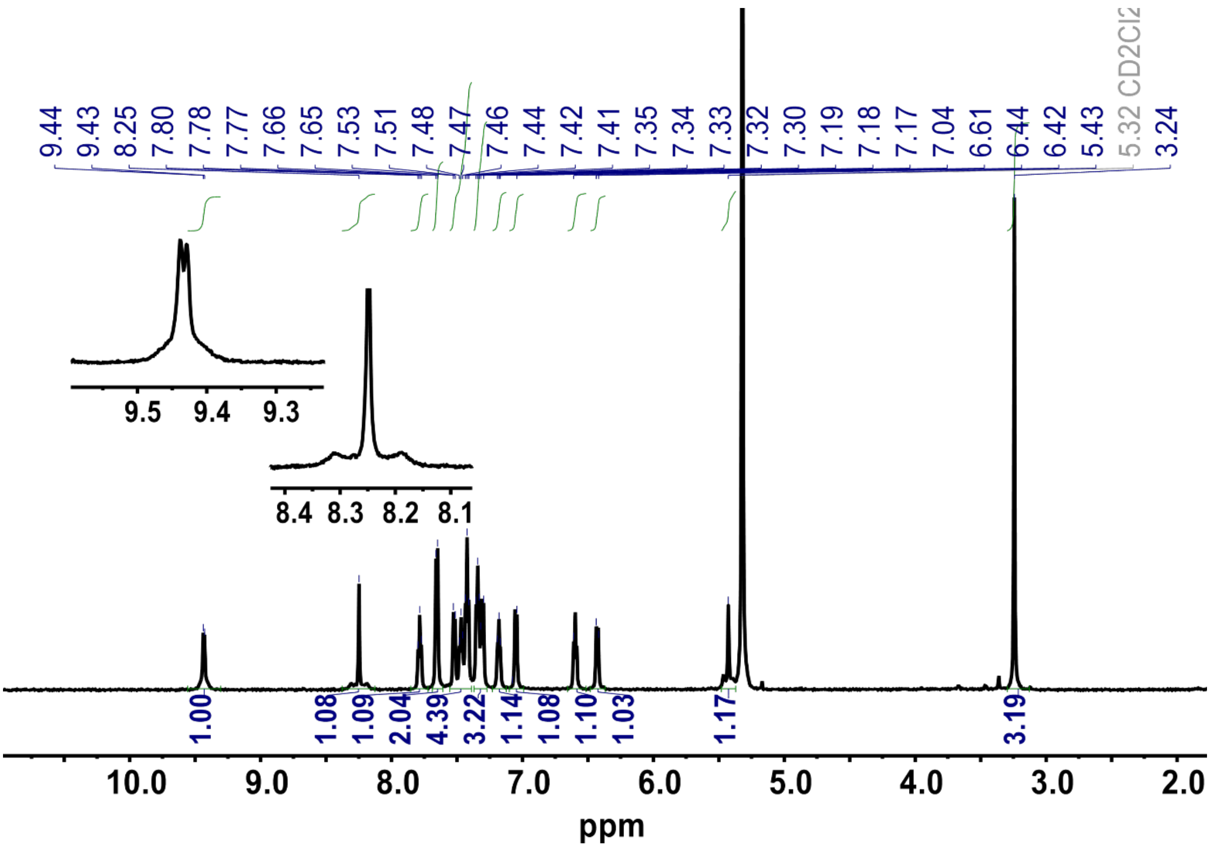

Figure S14. ^{1}H NMR spectrum of **1e** with insets to show ^{195}Pt satellites and multiplicity (600 MHz, CD_2Cl_2, 298K).

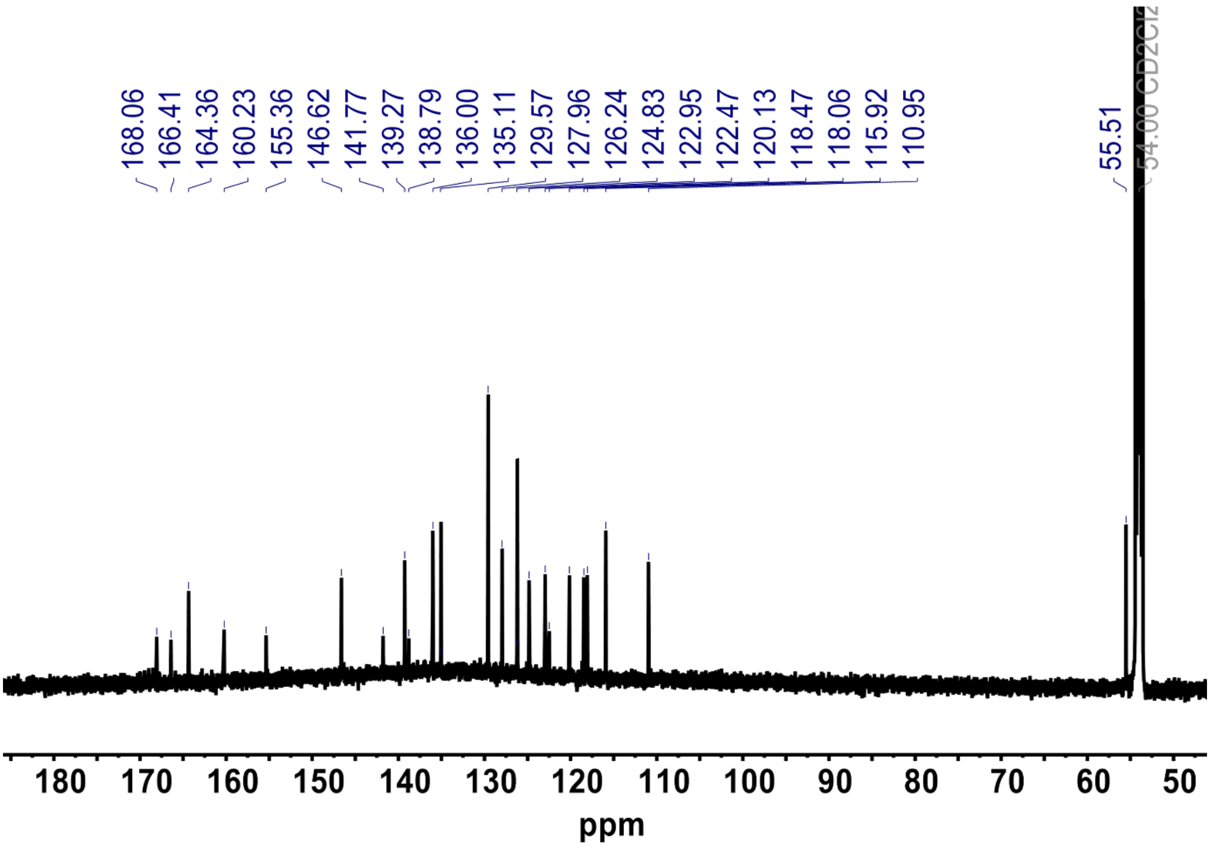

Figure S15. $^{13}C\{^1H\}$ NMR spectrum of **1e** (151 MHz, CD_2Cl_2, 298 K).

2.2.6 NMR spectra of 1f

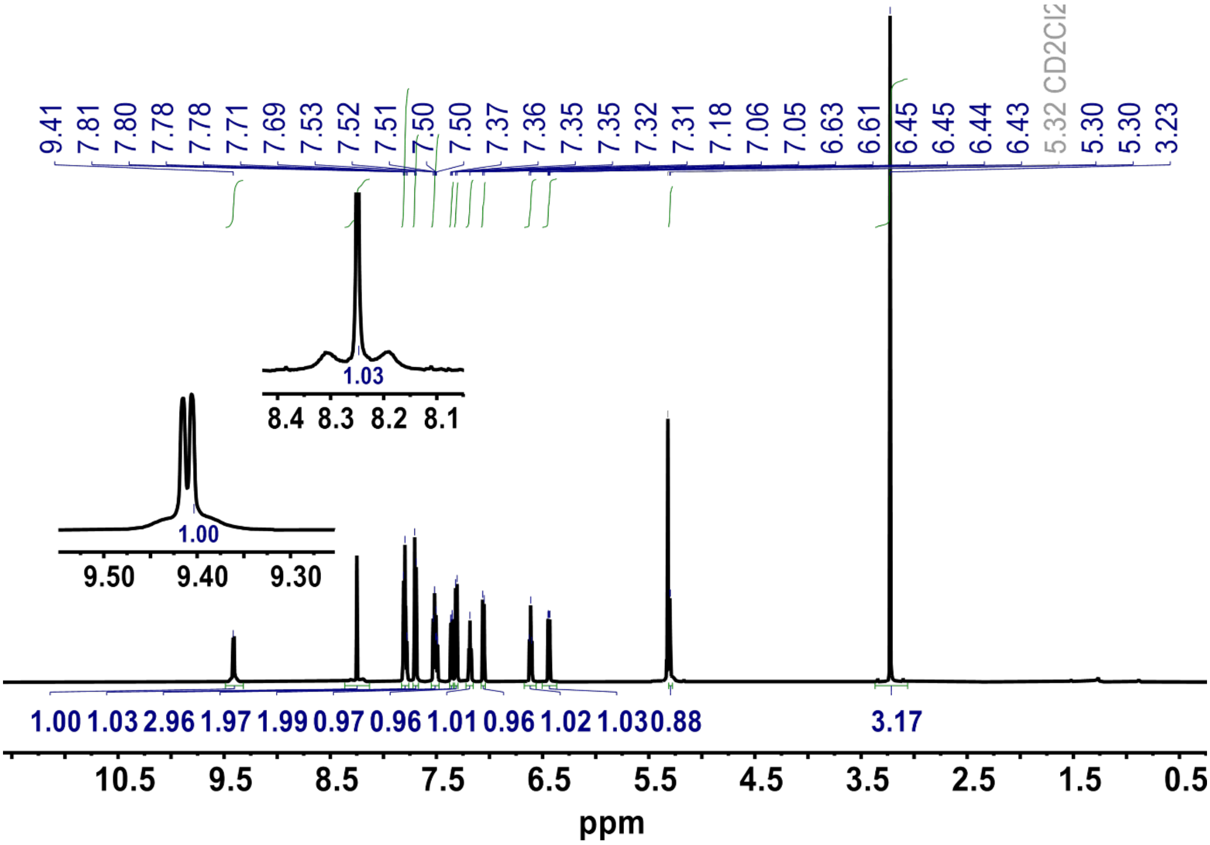

Figure S16. ^{1}H NMR spectrum of **1f** with insets to show ^{195}Pt satellites (600 MHz, CD_2Cl_2, 298K).

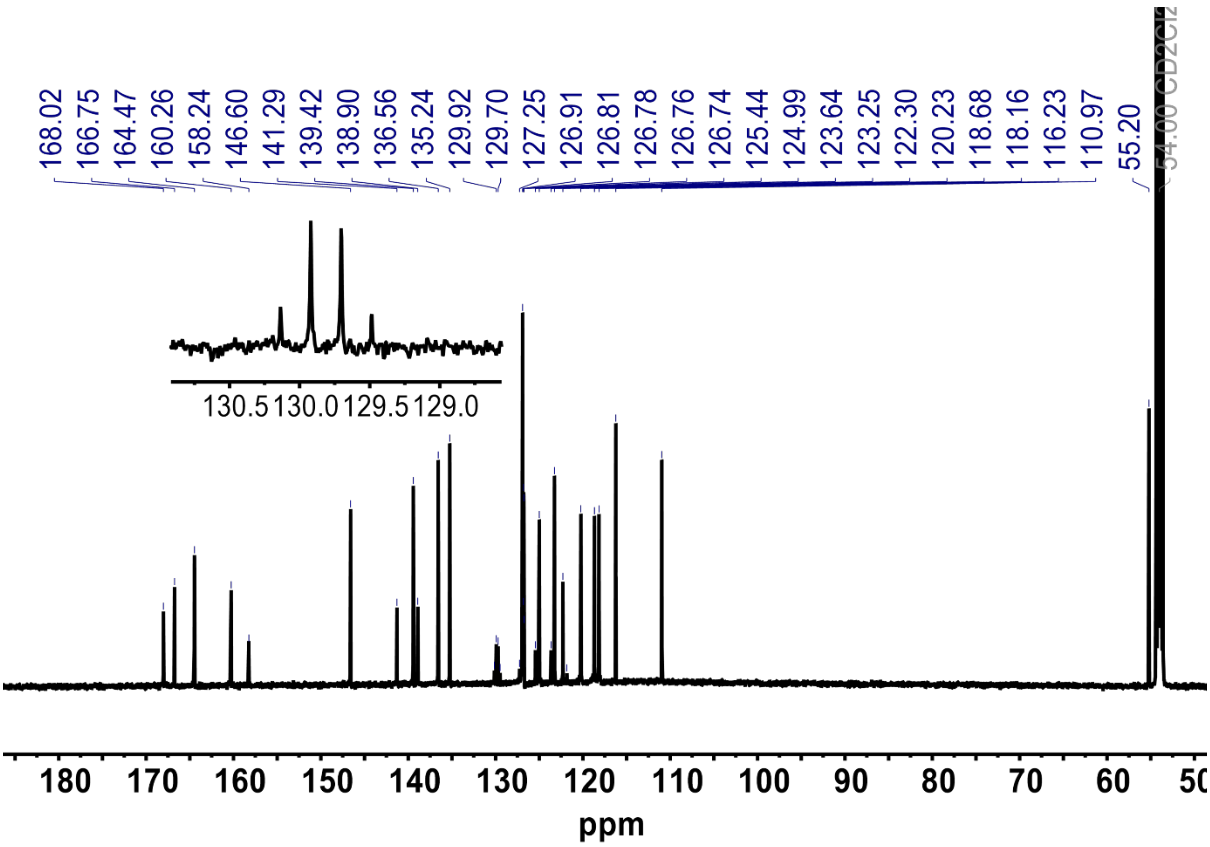

Figure S17. $^{13}C\{^1H\}$ NMR spectrum of **1f** with inset to show J_{C-F} coupling (151 MHz, CD_2Cl_2, 298 K).

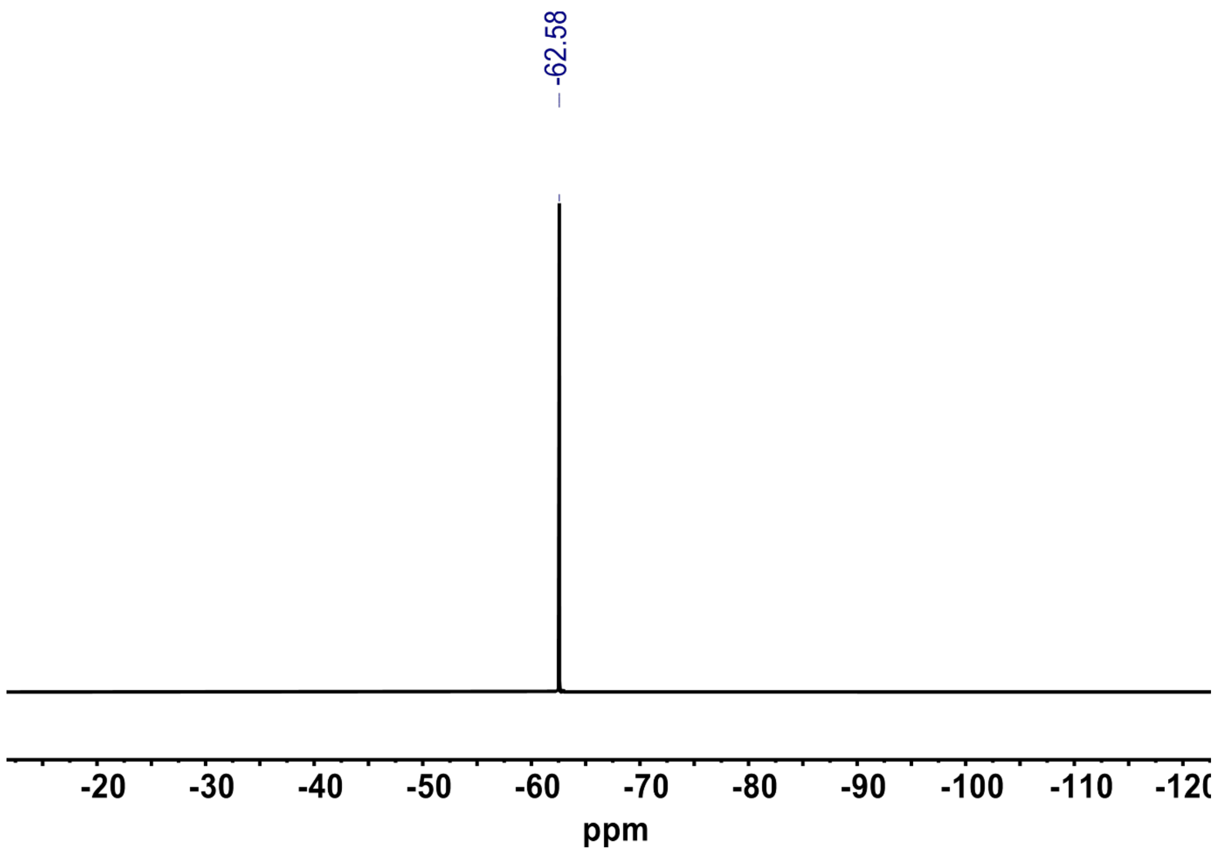

Figure S18. $^{19}F\{^{1}H\}$ NMR spectrum of **1f** (565 MHz, CD_2Cl_2, 298 K).

2.2.7 NMR spectra of 1g

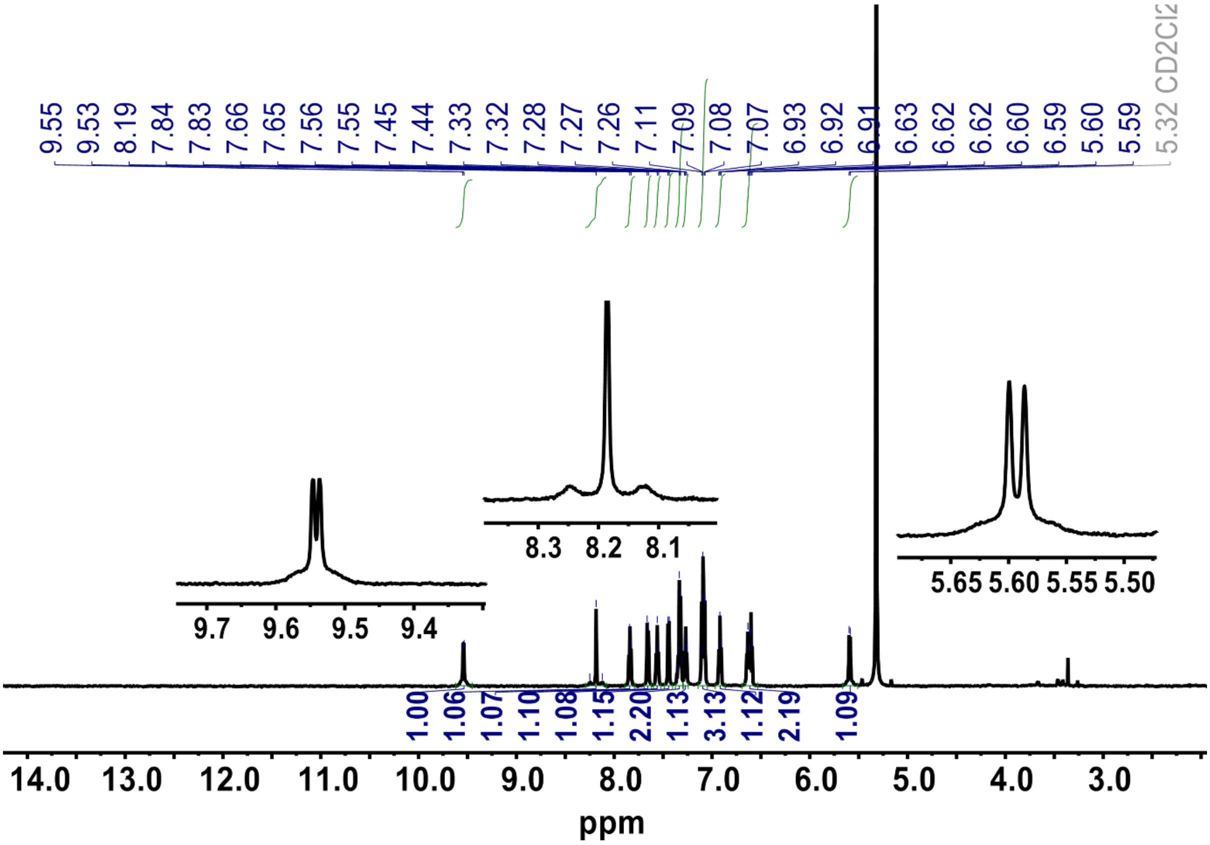

Figure S19. ^{1}H NMR spectrum of **1g** with insets to show ^{195}Pt satellites (600 MHz, CD_2Cl_2, 298K).

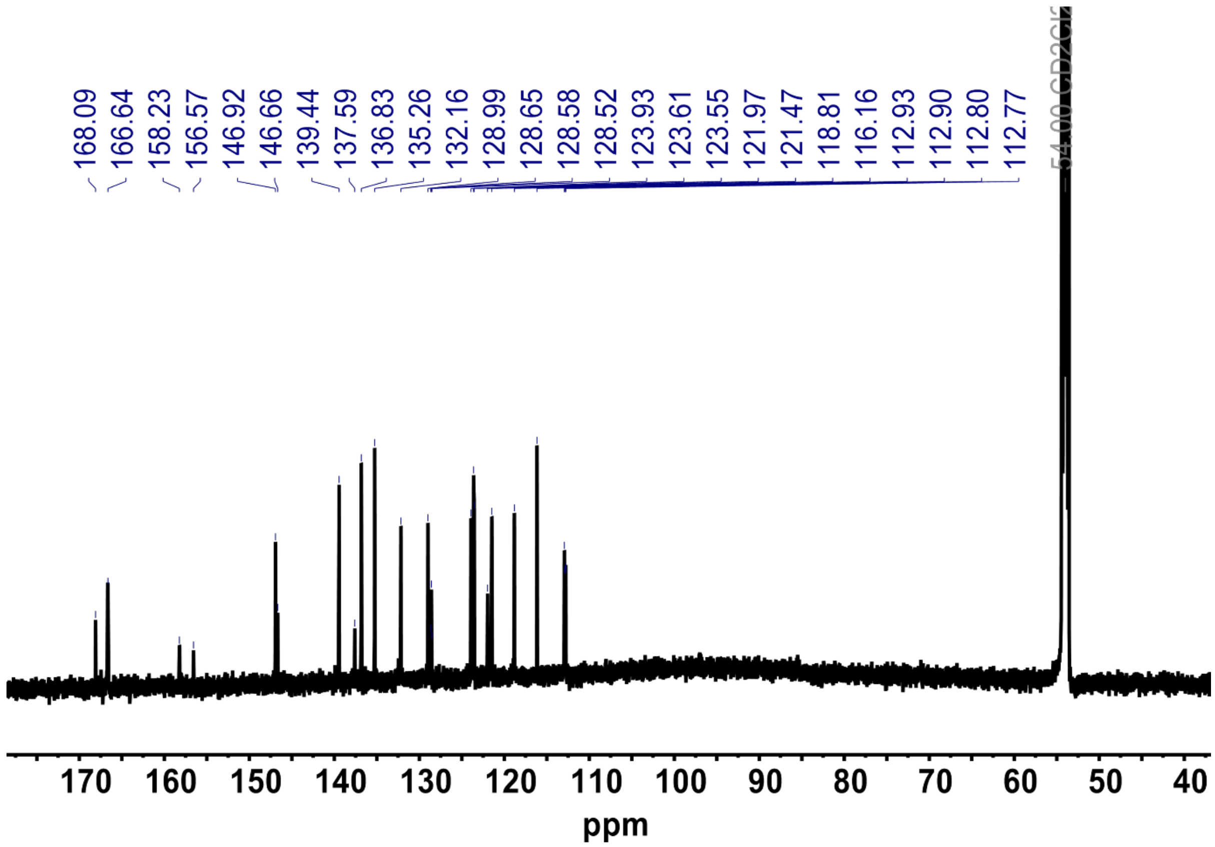

Figure S20. $^{13}C\{^1H\}$ NMR spectrum of **1g** (151 MHz, CD_2Cl_2, 298 K).

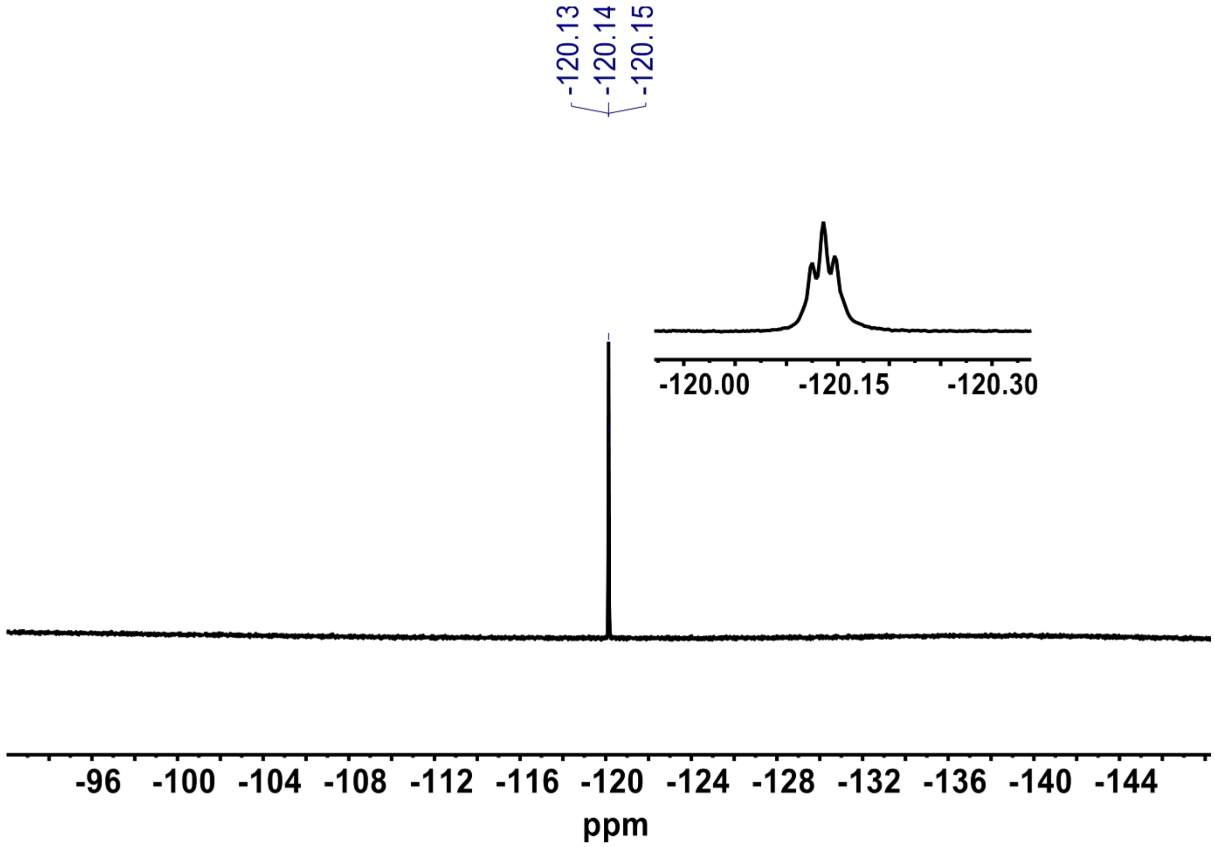

Figure S21. $^{19}F\{^1H\}$ NMR spectrum of **1g** (565 MHz, CD_2Cl_2, 298 K).

2.2.8 NMR spectra of 1h

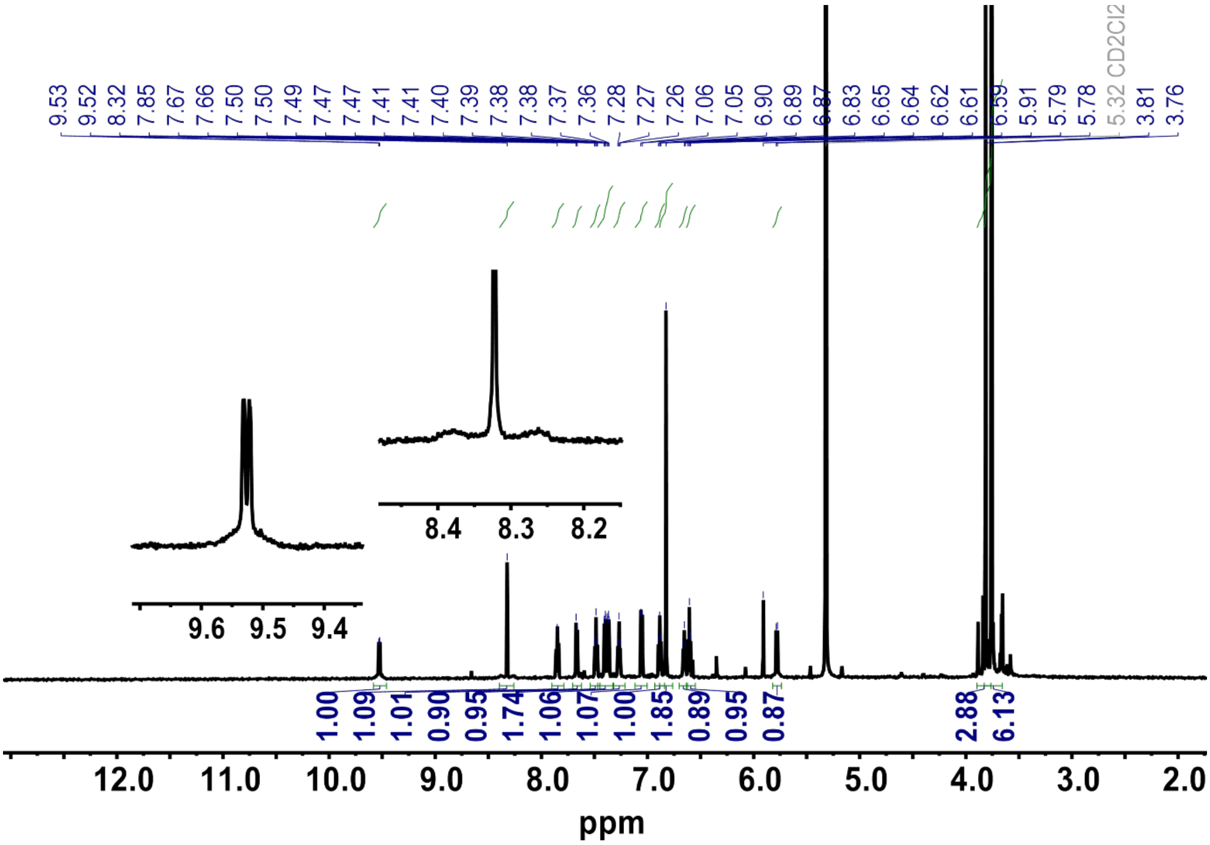

Figure S22. ^{1}H NMR spectrum of **1h** with insets to show ^{195}Pt satellites (600 MHz, CD_2Cl_2, 298K).

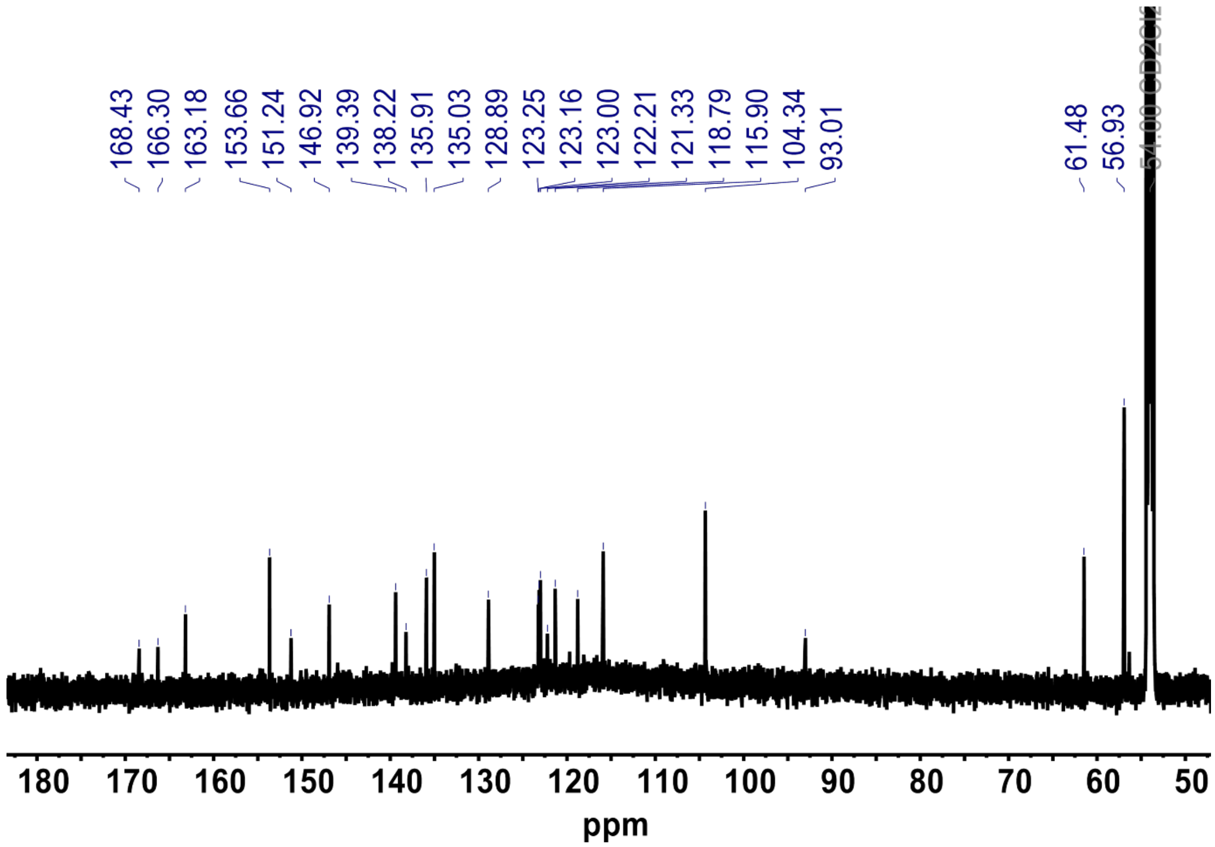

Figure S23. $^{13}C\{^{1}H\}$ NMR spectrum of **1h** (151 MHz, CD_2Cl_2, 298 K).

2.2.9 NMR spectra of d_1-hexamethylsiloxymethylsilane

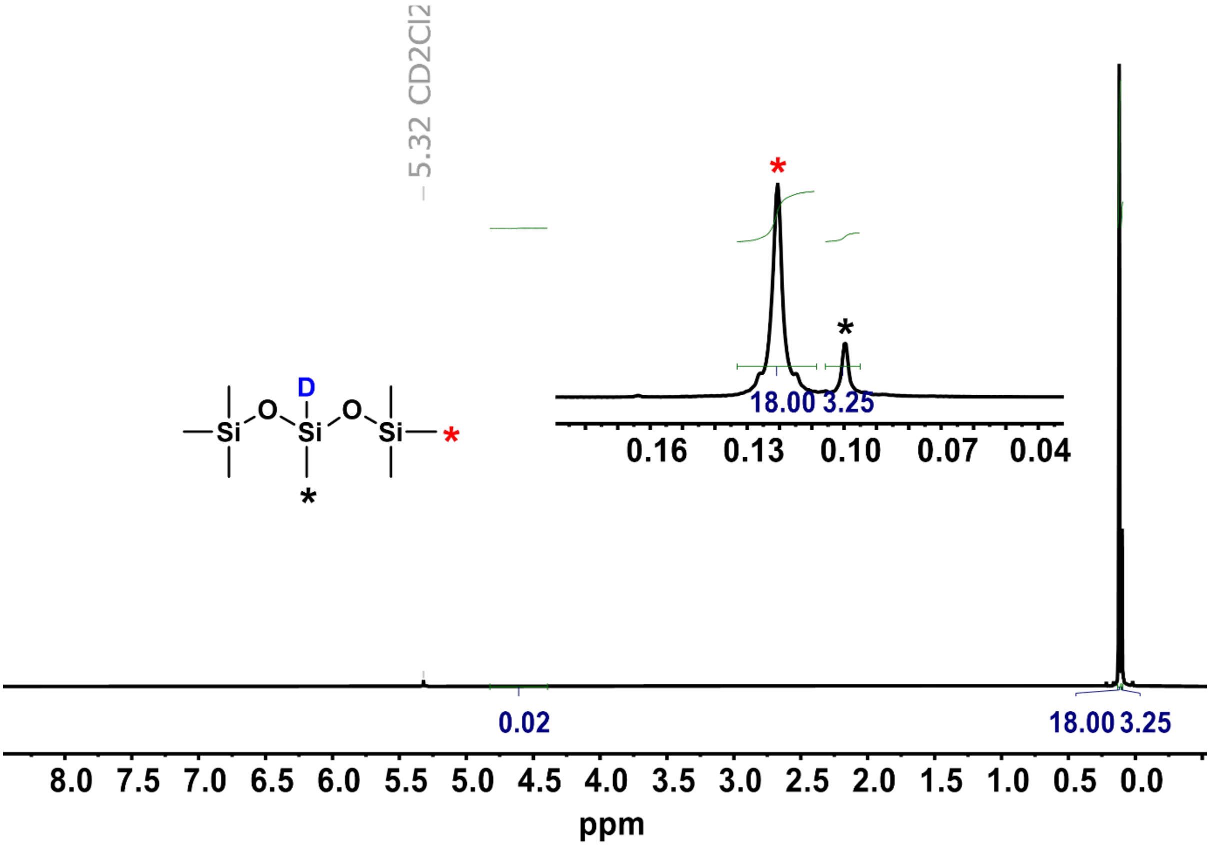

Figure S24. ^{1}H NMR spectrum of d_1-hexamethylsiloxymethylsilane (600 MHz, CD_2Cl_2, 298 K).

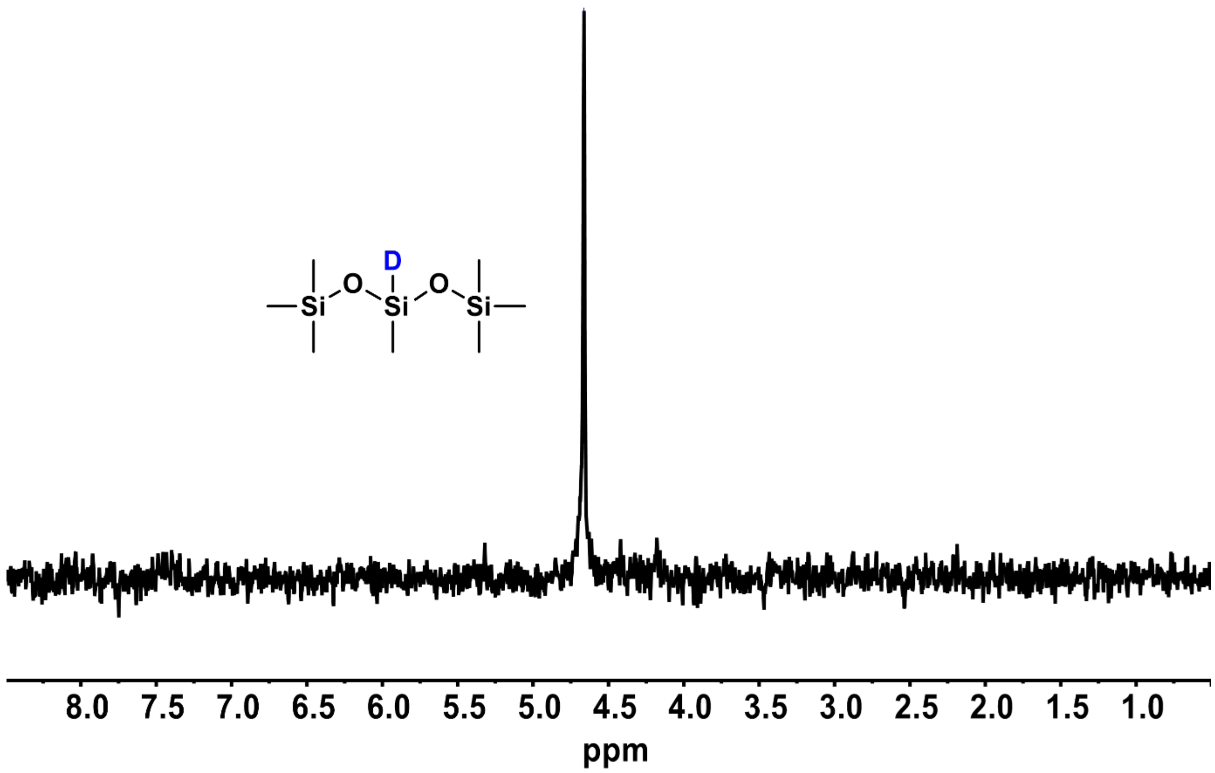

Figure S25. ^{2}H NMR of d_1-hexamethylsiloxymethylsilane (92 MHz, CH_2Cl_2, 298 K).

3 UV-Vis Spectra

3.1 dmso complexes

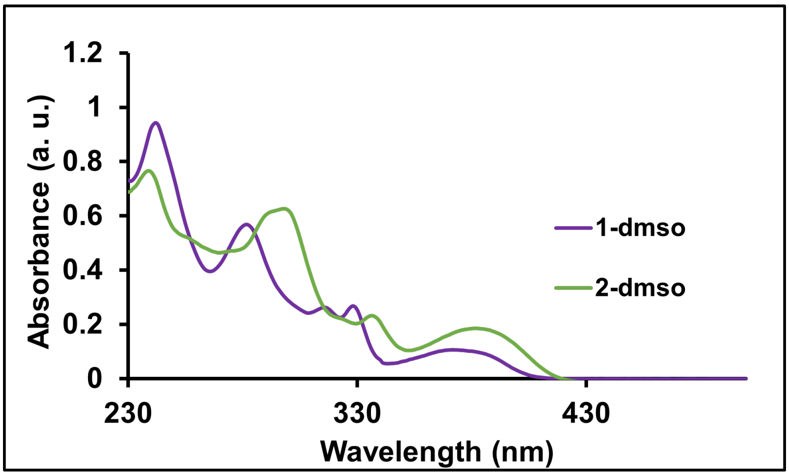

Figure S26. UV–vis absorption spectra of **1-dmso** and **2-dmso** in CH_2Cl_2 (1.6 x 10^{-5} M).

Table S1. Selected bands and absorption coefficients within the UV-vis spectra of **1-dmso** and **2-dmso**.

Complex	Absorption, λ_{max}, /nm [log(ε dm^3 mol^{-1} cm^{-1})]
**1-dmso**	242 (4.77), 282 (4.55), 316 (4.22), 328 (4.22)
**2-dmso**	239 (4.68), 298 (4.59), 336 (4.16), 381 (4.06)

3.2 Schiff base ligands

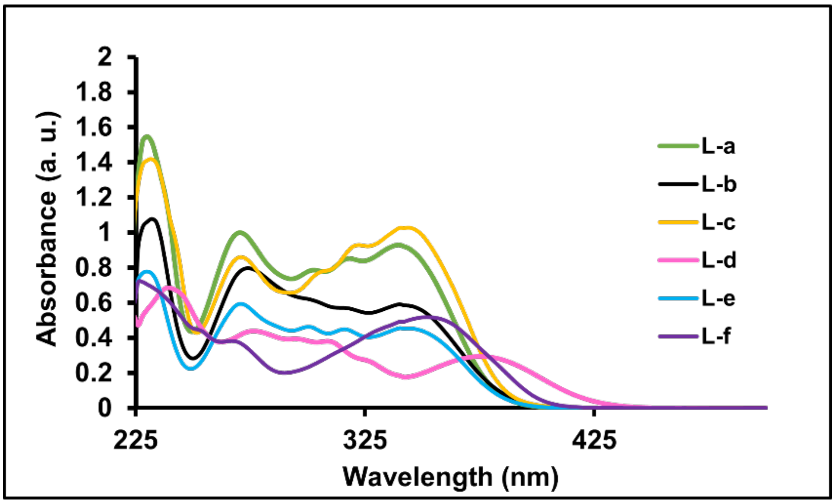

Figure S27. UV-vis absorption spectra of Schiff base ligands (**L-a**–**L-f**) in CH_2Cl_2 (1.6 x 10^{-5} M).

Table S2. Selected absorption bands and absorption coefficients within the UV–vis spectra of Schiff base ligands (**L-a**–**L-f**).

Ligand	Absorption, λ_{max}, /nm [log(ε dm^3 mol^{-1} cm^{-1})]
**L-a**	230 (4.99), 270 (4.80), 302 (4.69), 318 (4.73), 340 (4.76)
**L-b**	232 (4.83), 274 (4.70), 340 (4.57)
**L-c**	232 (4.95), 270 (4.73), 308 (4.69), 322 (4.76), 344 (4.81)
**L-d**	240 (4.63), 274 (4.43), 294 (4.39), 308 (4.38), 376 (4.26)
**L-e**	230 (4.69), 270 (4.57), 300 (4.46), 316 (4.45), 344 (4.45)
**L-f**	266 (4.38), 352 (4.51)

3.3 Pt(sal)(ppy) complexes

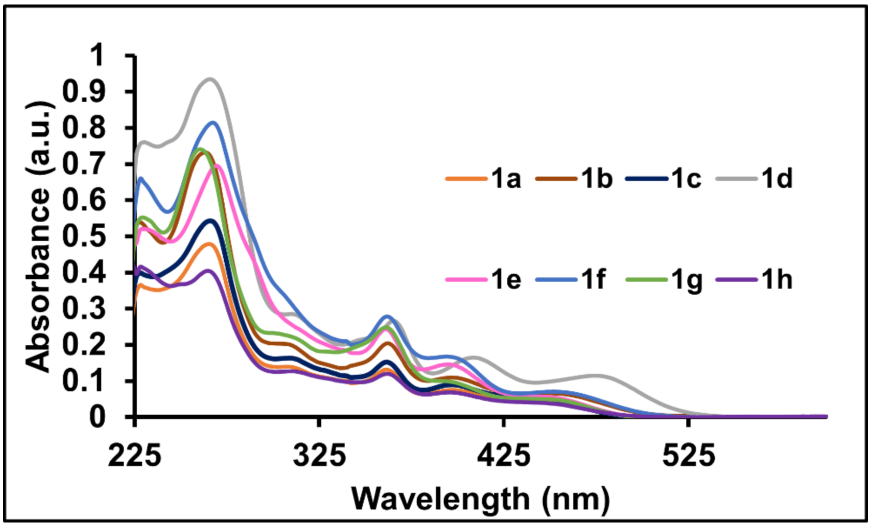

Figure S28. UV–vis absorption spectra of Pt(sal)(ppy) complexes (**1a–1h**) in CH_2Cl_2 (1.6 x 10^{-5} M).

Table S3. Selected absorption bands and extinction coefficients within the UV–vis absorption spectra of Pt(sal)(ppy) complexes (**1a–1h**) in CH_2Cl_2 (1.6 x 10^{-5} M).

Precatalyst	Absorption, λ_{max}, /nm [log(ε dm^3 mol^{-1} cm^{-1})]
**1a**	228 (4.33), 266 (4.45), 308 (3.91), 362 (3.89), 398 (3.64)
**1b**	228 (4.55), 264 (4.69), 362 (4.13), 396 (3.86)
**1c**	266 (4.54), 310 (4.02), 362 (3.99), 398 (3.76)
**1d**	230 (4.68), 266 (4.77), 310 (4.25), 366 (4.22), 408 (4.01), 476 (3.86)
**1e**	230 (4.51), 270 (4.63), 360 (4.18), 394 (3.96)
**1f**	228 (4.66), 268 (4.75), 362 (4.29), 394 (4.07)
**1g**	230 (4.54), 260 (4.67), 300 (4.17), 362 (4.19)
**1h**	228 (4.45), 264 (4.44), 310 (3.94), 362 (3.92), 396 (3.67)

4 In-situ catalysis

4.1 Method for in-situ reaction monitoring

All manipulations were performed under a nitrogen atmosphere using standard Schlenk line and glove-box techniques. Glassware was oven-dried at 130 °C overnight and flamed under vacuum prior to use. NMR tubes for hydrosilylation procedures were wrapped in foil during transportation to avoid light activation. The d1 time was set as 45 s to ensure accurate quantitative analysis. To assess and compare the catalytic activity of precatalysts **1a**–**1h** in the model hydrosilylation reaction, the precatalyst (0.0025 M) was loaded into a J. Youngs NMR tube and to it was added d_2-dichloromethane, along with hexamethylsiloxymethylsilane (0.8 – 1.0 M), vinyltrimethylsilane (0.8 – 1.0 M) and mesitylene (as internal standard, 0.10 M) from a stock solution. These molarities represent the final molarities after the dichloromethane has been added. The sample was inserted into the NMR spectrometer and an array of ~20 ^{1}H NMR spectra was acquired to determine the thermal latency period over ~1 h. The sample was then removed from the spectrometer and irradiated using the bespoke 365 nm LED reactor for either 10, 60 or 120 s before being returned to the spectrometer (note that this step is omitted for experiments conducted under thermal conditions). An array of 10–100 ^{1}H NMR spectra was acquired with appropriate delays between each acquisition (depending on the catalytic activity) and the concentrations of hexamethylsiloxymethylsilane, vinyltrimethylsilane and linear product were calculated using the absolute quantitation method. The absolute NMR integrals of the Si–**H** (δ 4.6), olefinic C**H** (δ 6.2) and Si–C$\mathbf{H}_2$–C$\mathbf{H}_2$–Si (δ 0.40) resonances were compared with the (C$\mathbf{H}_3$)$_3$ (δ 2.3) resonance of the internal standard. The kinetics were probed using the initial rates method, monitoring the rate of linear product formation over the first 3–5 data points after catalytic activity had been established. For the thermal profiles with an associated induction period, the initial rate was measured after the induction period, at the maximum rate of turnover. Errors were calculated using the linear regression model (LINEST) in Microsoft Excel. The reported errors are the estimated standard error from the model.

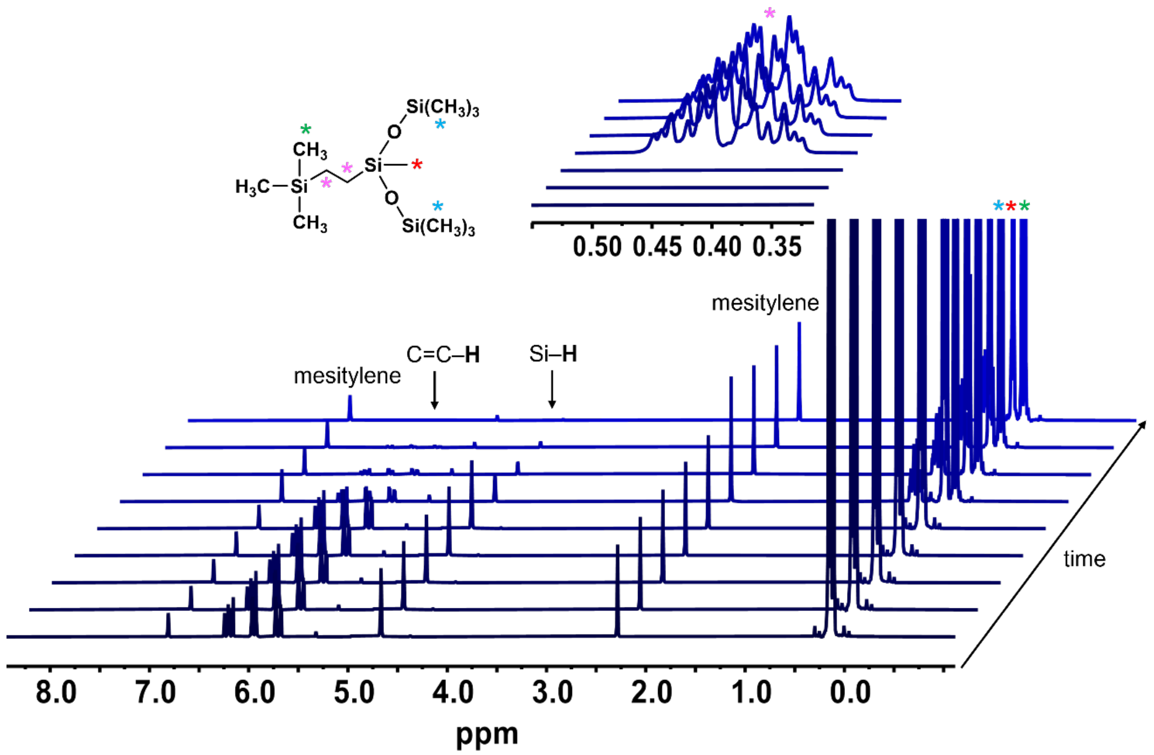

Figure S29. Representative stacked ^{1}H NMR spectra of the thermal in-situ hydrosilylation reaction between hexamethylsiloxymethylsilane and vinyltrimethylsilane over time using precatalyst **1b** to show consumption of substrates and formation of the linear product. First spectrum is after 30 min and final spectrum is after 23 h (400 MHz, CD_2Cl_2, 298 K).

4.2 Thermally activated catalysis

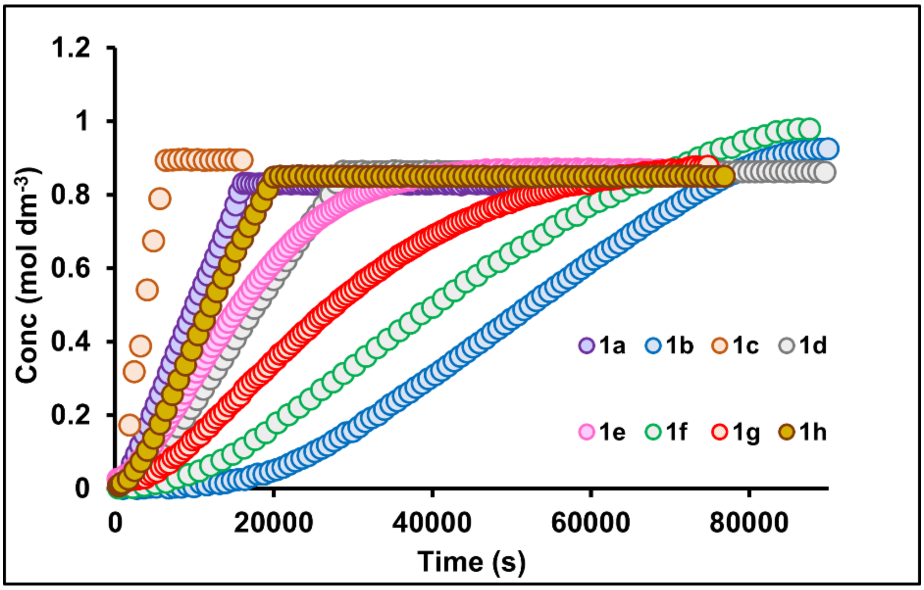

Figure S30. Overlaid concentration–time profiles of linear product formation from all precatalysts (**1a**–**1h**) in the thermal hydrosilylation reaction between hexamethylsiloxymethylsilane and vinyltrimethylsilane. Reaction conditions: 0.0025 M precatalyst, 0.8 – 1.0 M hexamethylsiloxymethylsilane and vinyltrimethylsilane in d_2-dichloromethane, 298 K.

Alkene and Silane concentrations

**Catalyst**	Alkene/M	Silane/M
**1a**	0.82	0.83
**1b**	0.90	0.91
**1c**	0.90	0.96
**1d**	0.83	0.87
**1e**	0.92	0.83
**1f**	1.00	1.06
**1h**	0.80	0.84

4.3 Photoactivated catalysis

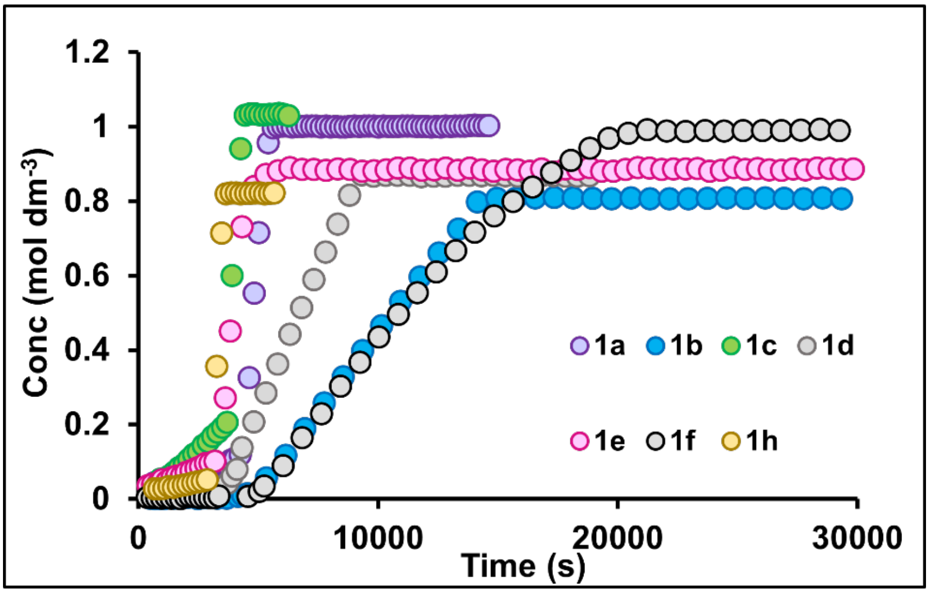

Figure S31. Overlaid concentration–time profiles of linear product formation from all precatalysts (**1a**–**1h**) in the photoactivated hydrosilylation reaction between hexamethylsiloxymethylsilane and vinyltrimethylsilane. Reaction conditions: 0.0025 M precatalyst, 0.8 – 1.0 M hexamethylsiloxymethylsilane and vinyltrimethylsilane in d_2-dichloromethane, 298 K. All reactions were monitored for *ca.* 1 h to examine the latency period and then irradiated for 10 s with the 365 nm LED reactor.

Alkene and Silane concentrations

**Catalyst**	Alkene/M	Silane/M
**1a**	1.04	1.03
**1b**	0.80	0.85
**1c**	1.04	1.03
**1d**	0.86	0.87
**1e**	0.92	0.90
**1f**	1.00	1.06
**1h**	0.85	0.85

4.4 ^{1}H NMR data of precatalyst after photoactivation and catalysis

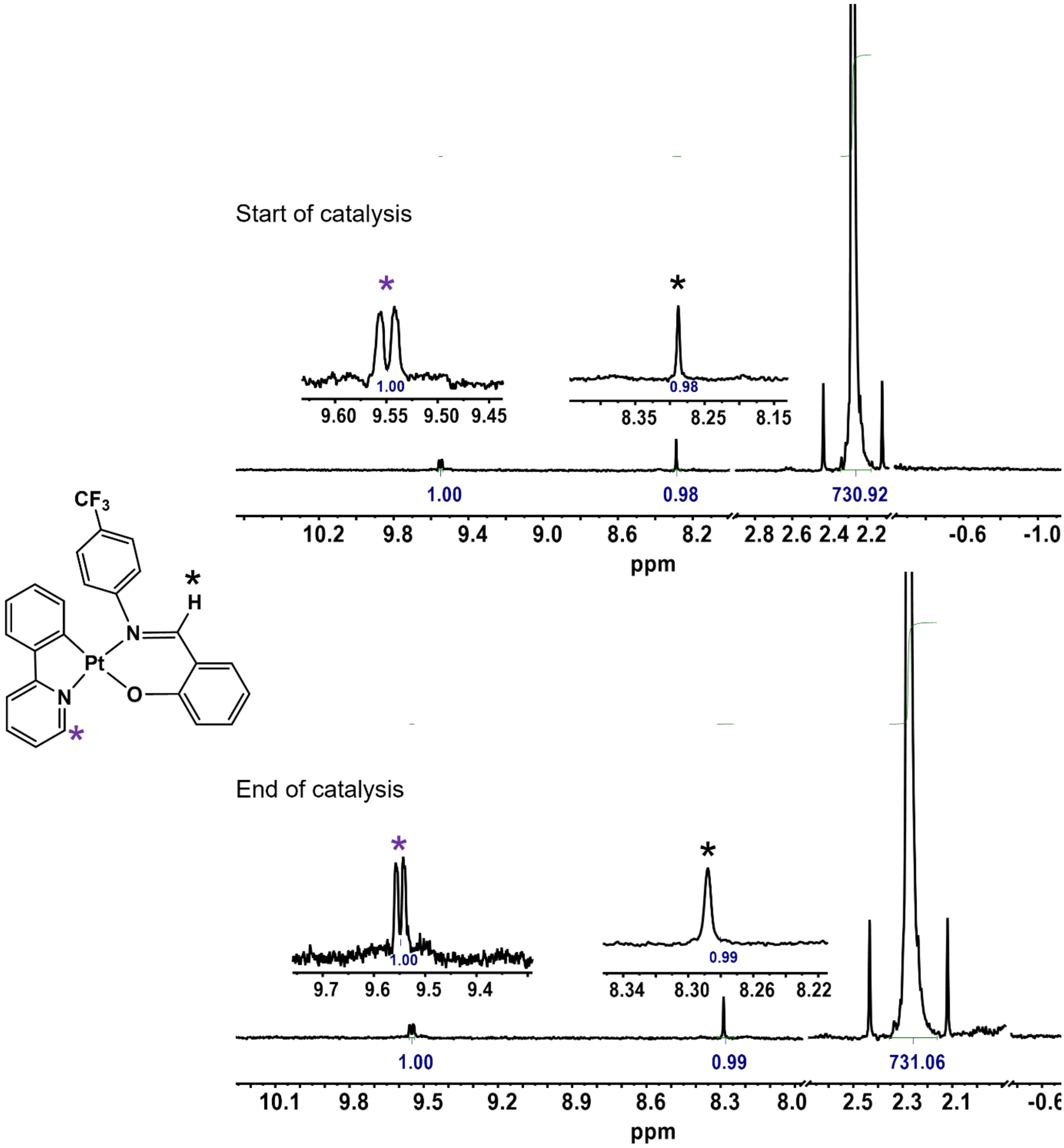

Figure S32. ^{1}H NMR spectra of the hydrosilylation reaction between hexamethylsiloxymethylsilane and vinyltrimethylsilane using **1b** as a precatalyst (400 MHz, CD_2Cl_2, 298 K, irradiation time = 10s). Reaction conditions as stated in the method for in-situ reaction monitoring section 4.1.The top spectrum is the reaction mixture at the start of catalysis and the bottom spectrum is at the end of catalysis. The inset shows that there is no observable change in the catalyst after catalysis has finished.

4.5 Substrate Scope

4.5.1 Oct-1-ene

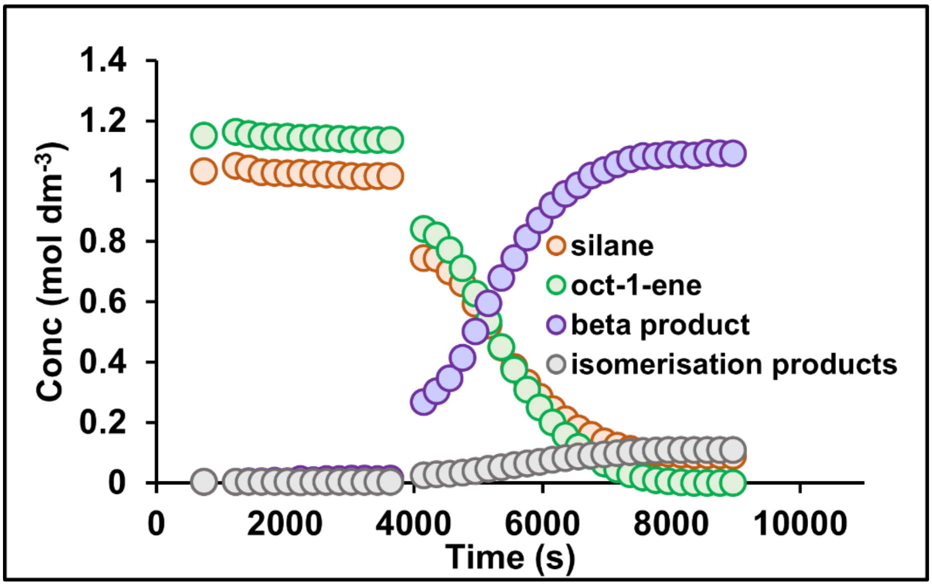

Figure S33. Concentration-time profile of hexamethylsiloxymethylsilane and oct-1-ene consumption and beta and isomerisation product formation during the photoactivated hydrosilylation reaction using **1b** as a precatalyst. Reaction conditions (0.0025 M **1b**, 1.1 M hexamethylsiloxymethylsilane and 1.1 M oct-1-ene in d_2-dichloromethane, left for ~1 h thermally then irradiated for 120 s).

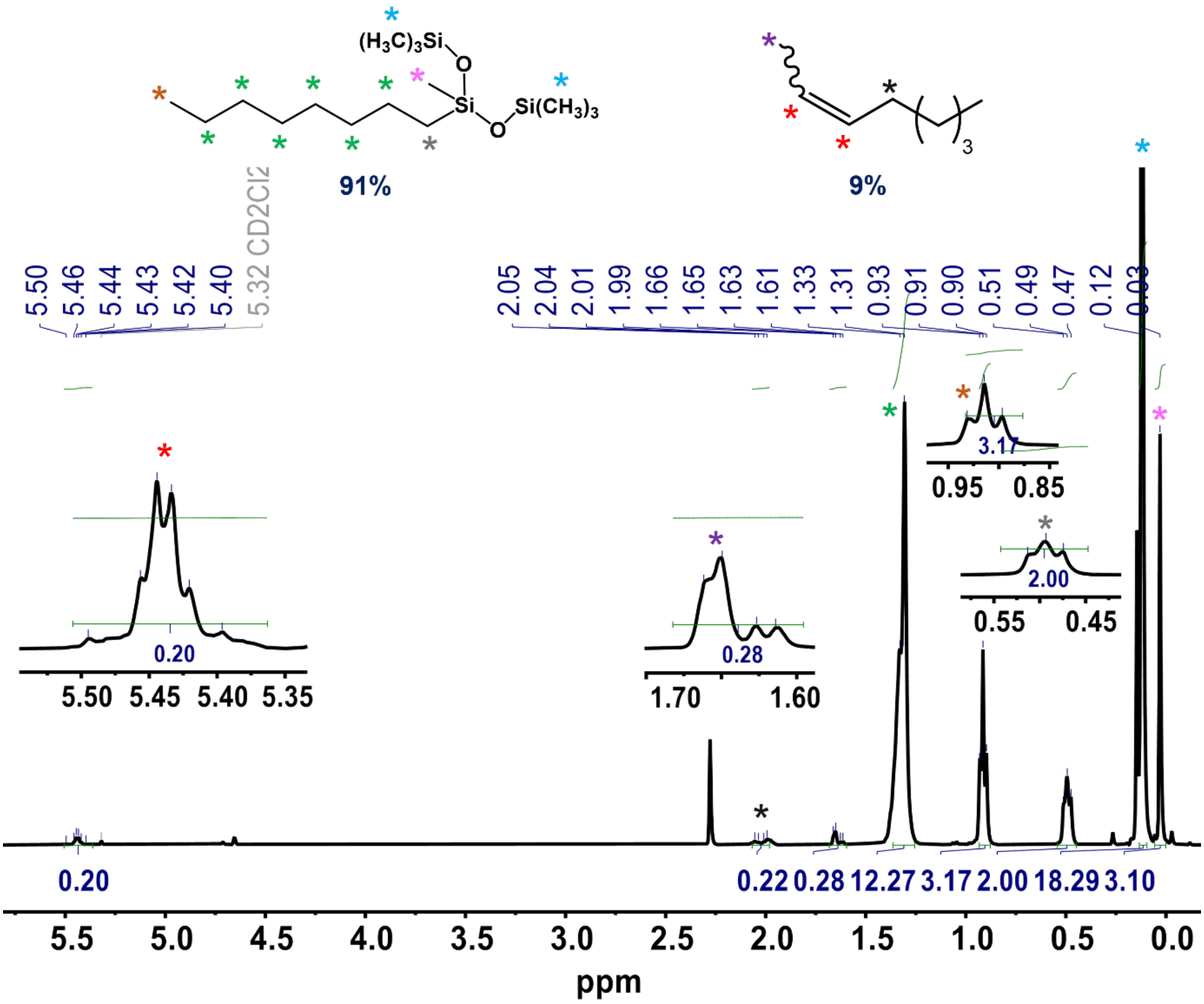

Figure S34. ^{1}H NMR spectrum of the beta and isomerisation products formed in the in-situ hydrosilylation reaction of hexamethylsiloxymethylsilane and oct-1-ene using precatalyst **1b** (400 MHz, d_2-dichloromethane, 298 K). Reaction conditions (0.0025 M **1b**, 1.1 M hexamethylsiloxymethylsilane, 1.1 M oct-1-ene, left for ~1 h thermally then irradiated for 120 s). Selectivity: 91% beta product and 9% isomerisation products.

4.5.2 Styrene

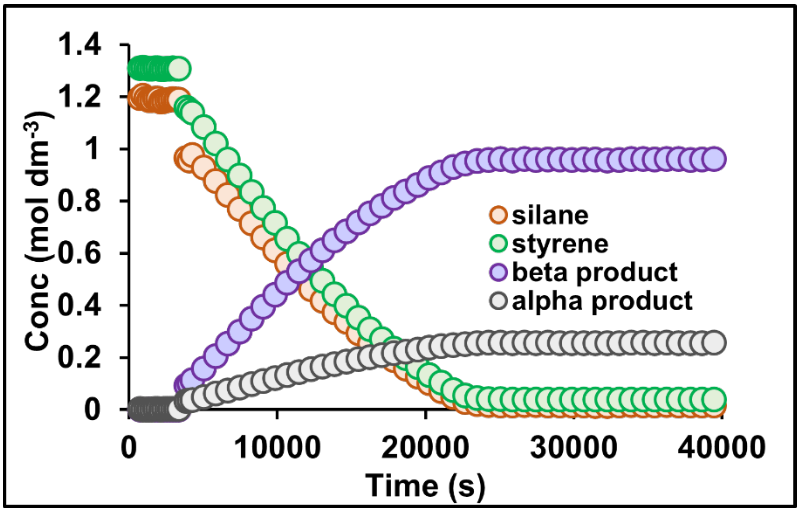

Figure S35. Concentration-time profile of hexamethylsiloxymethylsilane and styrene consumption and alpha and beta product formation during the photoactivated hydrosilylation reaction using **1b** as a precatalyst. Reaction conditions (0.0025 M **1b**, 1.2 M hexamethylsiloxymethylsilane and 1.2 M styrene in d_2-dichloromethane, left for ~1 h thermally then irradiated for 120 s).

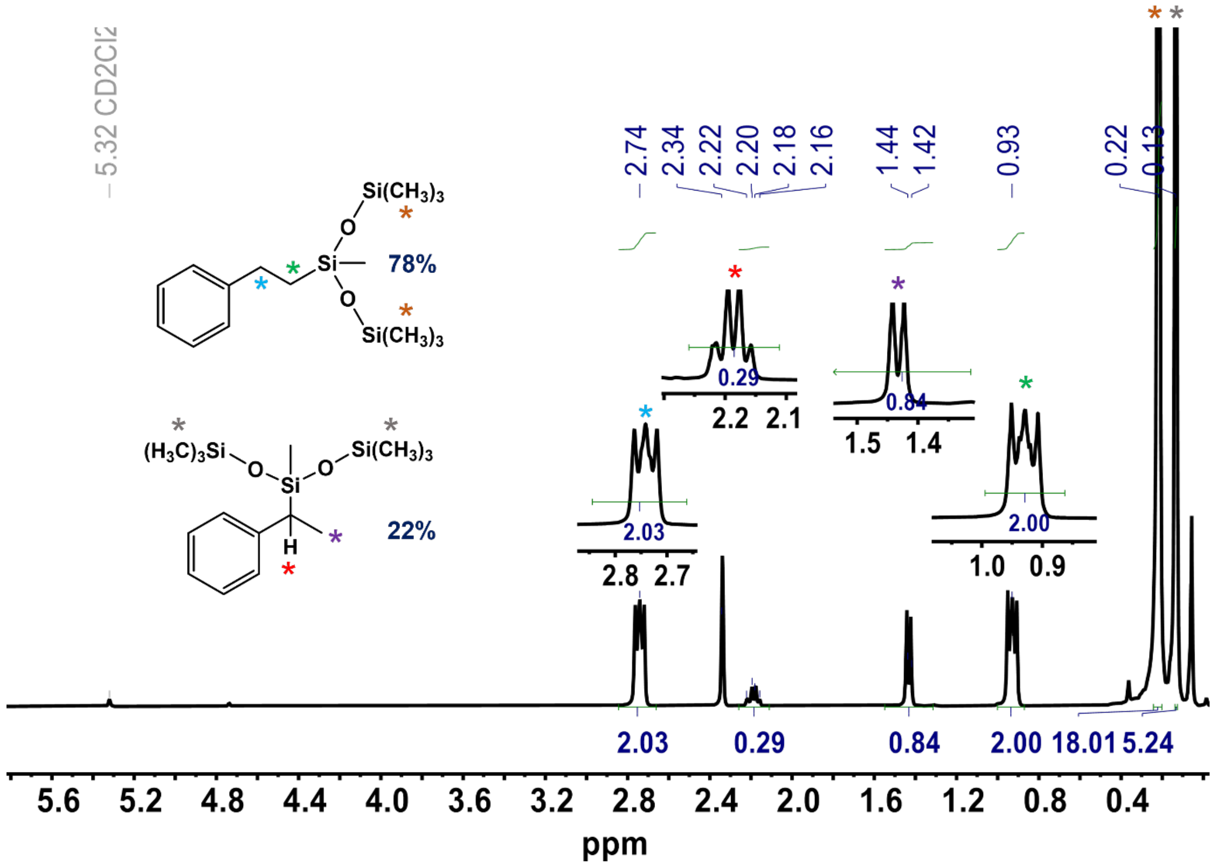

Figure S36. ^{1}H NMR spectrum of the alpha and beta products formed in the photoactivated hydrosilylation reaction of hexamethylsiloxymethylsilane and styrene using precatalyst **1b** (400 MHz, d_2-dichloromethane, 298 K). Reaction conditions (0.0025 M **1b**, 1.2 M hexamethylsiloxymethylsilane, 1.2 M styrene, left for ~1 h thermally then irradiated for 120 s). Selectivity: 78% beta product and 22% alpha product.

4.6 Addition of methanol

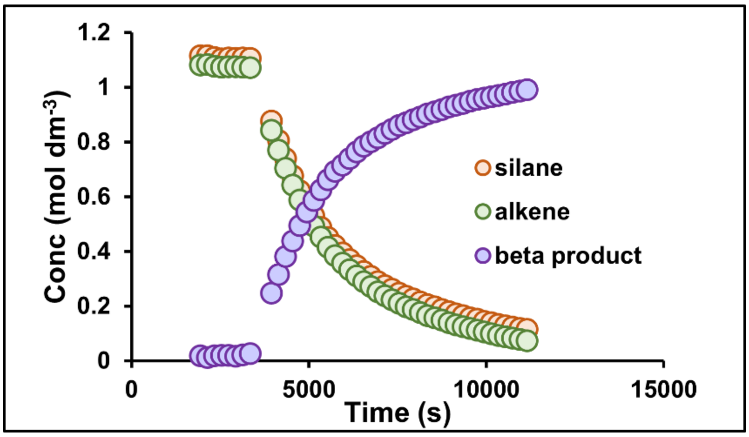

Figure S37. Concentration-time profile of hexamethylsiloxymethylsilane and vinyltrimethylsilane consumption and beta product formation during the photoactivated hydrosilylation reaction using **1b** as a precatalyst in the presence of excess methanol. Reaction conditions (0.0025 M **1b**, 1.1 M hexamethylsiloxymethylsilane, 1.1 M vinyltrimethylsilane and 0.02 M methanol in d_2-dichloromethane, left for ~1 h thermally then irradiated for 120 s).

4.7 Addition of a different Schiff base ligand

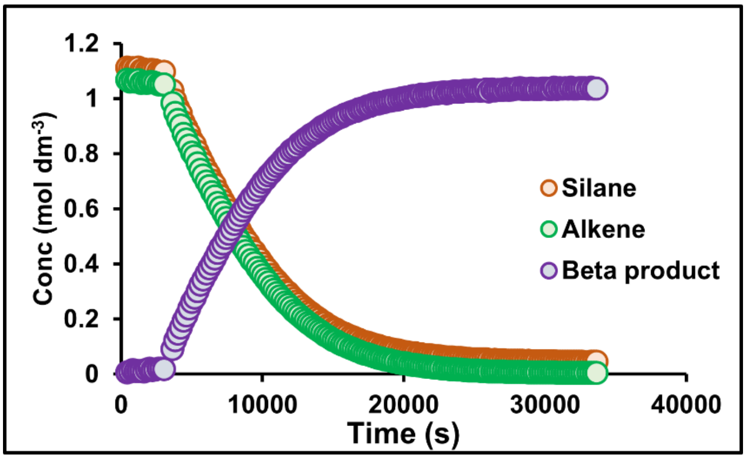

Figure S38. Concentration-time profile of hexamethylsiloxymethylsilane and vinyltrimethylsilane consumption and beta product formation during the photoactivated hydrosilylation reaction using **1b** as a precatalyst in the presence of a different Schiff base ligand. Reaction conditions (0.0025 M **1b**, 1.1 M hexamethylsiloxymethylsilane, 1.1 M vinyltrimethylsilane and 0.005 M Schiff base ligand of precatalyst **1h** in d_2-dichloromethane, left for ~1 h thermally then irradiated for 120 s).

4.8 Addition of TEMPO

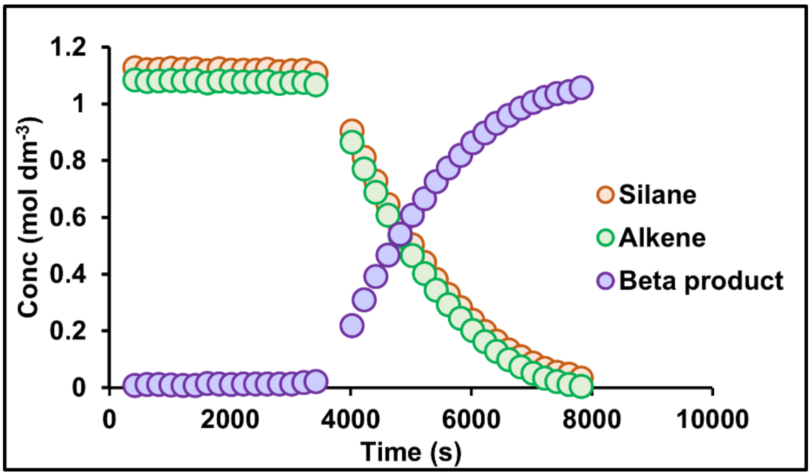

Figure S39. Concentration-time profile of hexamethylsiloxymethylsilane and vinyltrimethylsilane consumption and beta product formation during the photoactivated hydrosilylation reaction using **1b** as a precatalyst in the presence of excess TEMPO. Reaction conditions (0.0025 M **1b**, 1.1 M hexamethylsiloxymethylsilane, 1.1 M vinyltrimethylsilane and 0.02 M TEMPO in d_2-dichloromethane, left for ~1 h thermally then irradiated for 120 s).

5 Poisoning experiments for thermally activated catalysis

5.1 Hg poisoning

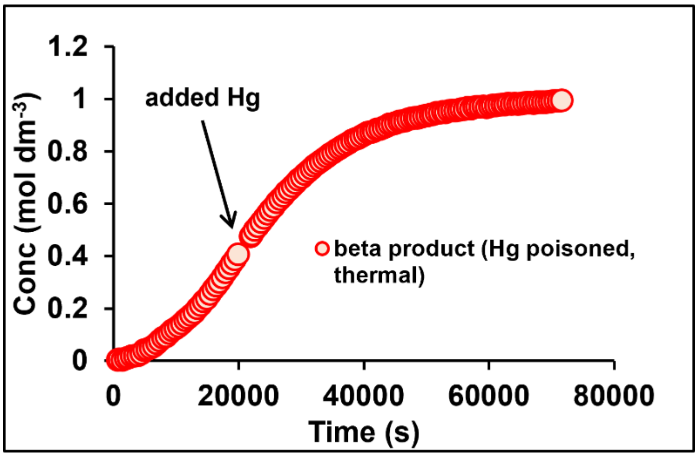

Figure S40. Concentration-time profile of linear product formation during the thermal hydrosilylation reaction between hexamethylsiloxymethylsilane and vinyltrimethylsilane using **1b** as a precatalyst with a drop of Hg added after *ca.* 20,000 s. Reaction conditions as stated in the method for in-situ reaction monitoring section 4.1.

Table S4. Initial rates for linear product formed via the thermal hydrosilylation reaction between hexamethylsiloxymethylsilane and vinyltrimethylsilane using **1b** as a precatalyst before and after the addition of mercury. Reaction conditions as stated in the method for in-situ reaction monitoring section 4.1.

	Initial Rate (mol dm^{-3} s^{-1})
rate before Hg	(2.20 ± 0.08) x 10^{-5}
rate after Hg	(2.57 ± 0.04) x 10^{-5}

5.2 DBCOT poisoning

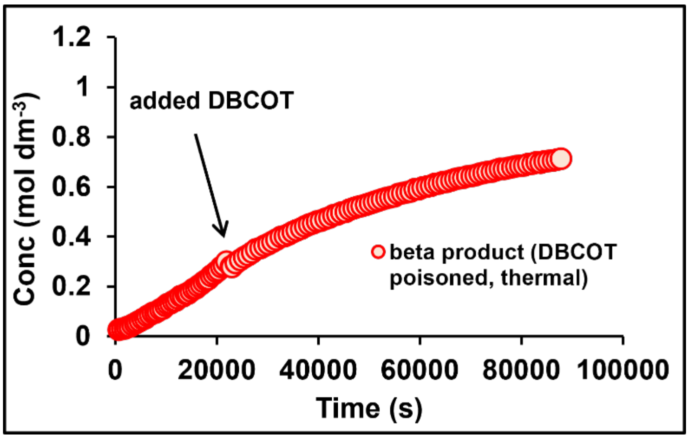

Figure S41. Concentration-time profile of linear product formation during the thermal hydrosilylation reaction between hexamethylsiloxymethylsilane and vinyltrimethylsilane using **1b** as a precatalyst with DBCOT added after *ca.* 20,000 s. Reaction conditions as stated in the method for in-situ reaction monitoring section 4.1

Table S5. Initial rates for linear product formed via the thermal hydrosilylation reaction between hexamethylsiloxymethylsilane and vinyltrimethylsilane using **1b** as a precatalyst before and after the addition of DBCOT. Reaction conditions as stated in the method for in situ reaction monitoring section 4.1.

	Initial Rate (mol dm^{-3} s^{-1})
rate before DBCOT	(2.06 ± 0.11) x 10^{-5}
rate after DBCOT	(1.55 ± 0.06) x 10^{-5}

6 Adding substrates before and after irradiation

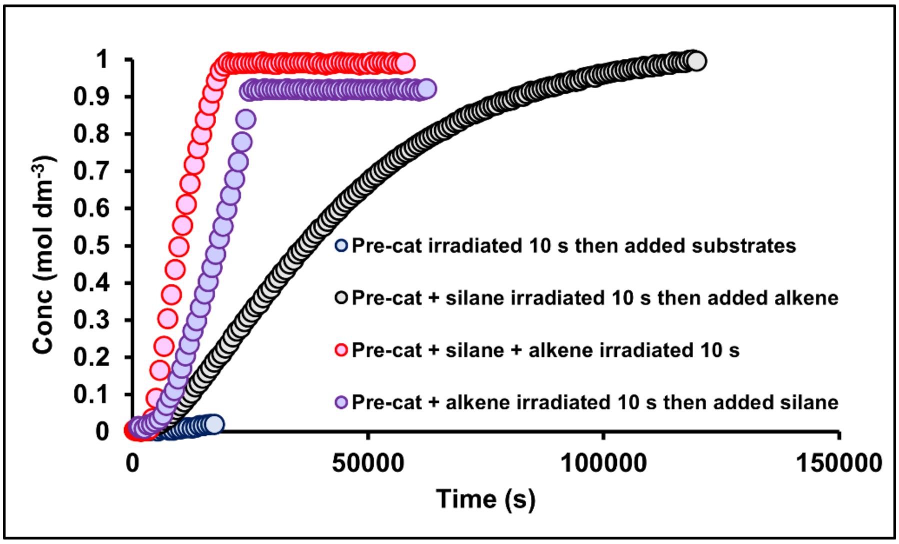

Figure S42. Concentration-time profiles of linear product formation during the photoactivated hydrosilylation reaction between hexamethylsiloxymethylsilane and vinyltrimethylsilane using precatalyst **1f**. Reaction conditions as stated in the method for in-situ reaction monitoring section 4.1. Blue: precatalyst **1f** irradiated 10 s then added hexamethylsiloxymethylsilane and vinyltrimethylsilane, black: precatalyst **1f** and hexamethylsiloxymethylsilane irradiated 10 s then added vinyltrimethylsilane, pink: precatalyst **1f**, hexamethylsiloxymethylsilane and vinyltrimethylsilane irradiated 10 s, purple: precatalyst **1f** and vinyltrimethylsilane irradiated 10 s then added hexamethylsiloxymethylsilane.

Table S6. Initial rates following irradiation in the photoactivated hydrosilylation reaction between hexamethylsiloxymethylsilane and vinyltrimethylsilane using **1f** as a precatalyst. Reaction conditions as stated in the method for in-situ reaction monitoring section 4.1, with 10 s irradiation.

Order of addition	Initial Rate (mol dm^{-3} s^{-1})
Pre-cat irradiated 10 s then added substrates	-
Pre-cat and silane irradiated 10 s then added alkene	$(1.25 \pm 0.05) \times 10^{-5}$
Pre-cat, silane and alkene irradiated 10 s	$(8.7 \pm 0.1) \times 10^{-5}$
Pre-cat and alkene irradiated 10 s then added silane	$(2.9 \pm 0.12) \times 10^{-5}$

7 TEM images

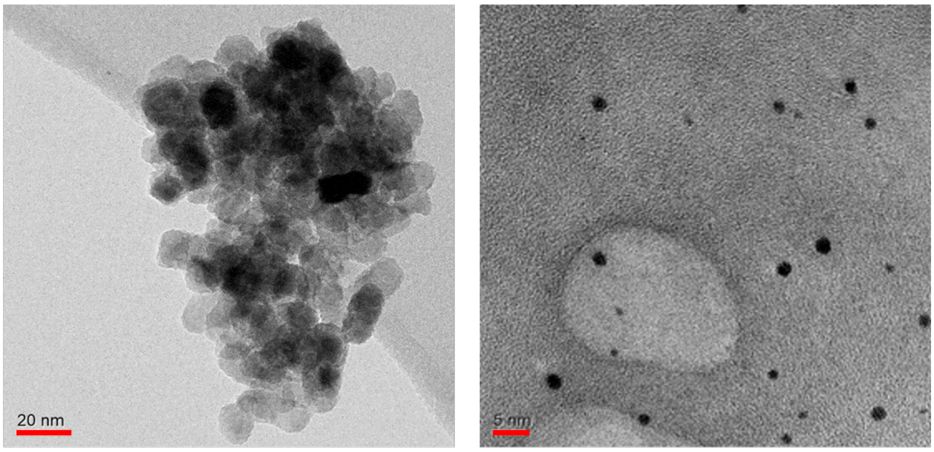

20 nm ***5 nm***

Figure S43. TEM images of the post-catalysis mixture (2 h). Reaction conditions as stated in the method for in-situ reaction monitoring section 4.1, with 10 s irradiation.

8 Dynamic light scattering

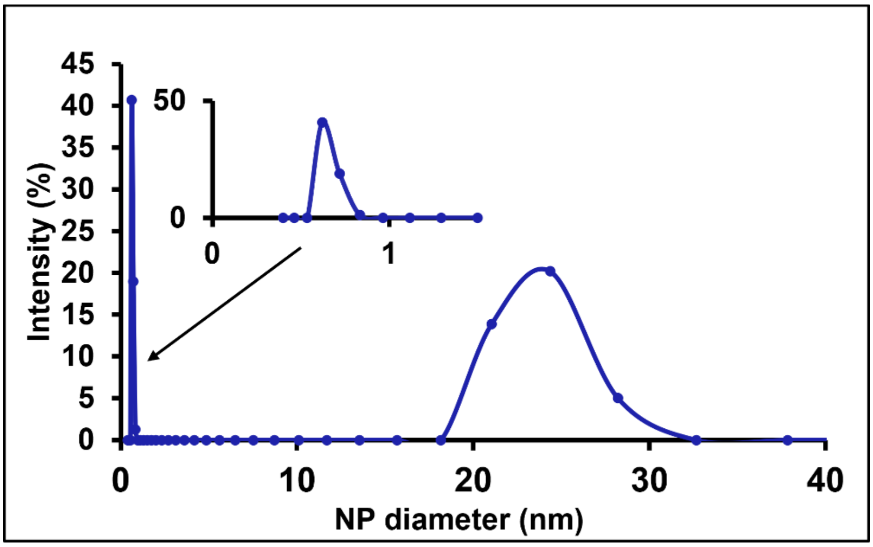

Figure S44. DLS data of the post-catalysis mixture (2 h). Reaction conditions as stated in the method for in-situ reaction monitoring section 4.1, with 10 s irradiation.

9 Determining order in silane and alkene post irradiation

9.1 Precatalyst 1b order in silane (post 120 s irradiation)

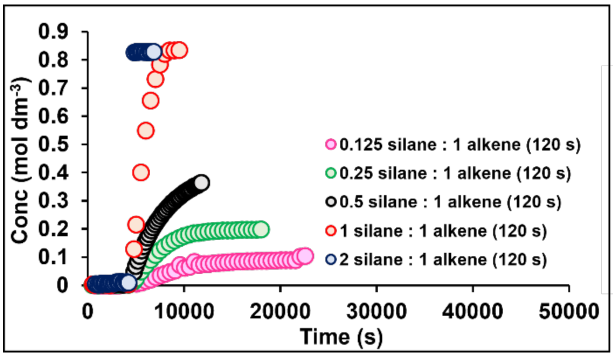

Ratio	Initial Rate (mol dm^{-3} s^{-1})
0.125 silane : 1 alkene	$(1.17 \pm 0.11) \times 10^{-5}$
0.25 silane : 1 alkene	$(4.5 \pm 0.1) \times 10^{-5}$
0.5 silane : 1 alkene	$(9.4 \pm 0.2) \times 10^{-5}$
1 silane : 1 alkene	$(5.23 \pm 0.12) \times 10^{-4}$
2 silane : 1 alkene	-

Figure S45. Concentration-time profiles of linear product formation during the photoactivated hydrosilylation reaction between hexamethylsiloxymethylsilane and vinyltrimethylsilane using **1b** as a precatalyst. Reaction conditions: 0.0025 M **1b**, 0.11 M, 0.22 M, 0.44 M, 0.88 M and 1.76 M hexamethylsiloxymethylsilane, 0.88 M vinyltrimethylsilane in d_2-dichloromethane, left for ~1 h thermally then irradiated 120 s with 365 nm LED reactor, 298 K). All plots are time-shifted to show the same latency period. The table shows the initial rates following irradiation. Concentrations determined from the signal integrals from the in-situ monitored ^{1}H NMR spectra and are relative to mesitylene (integration standard).

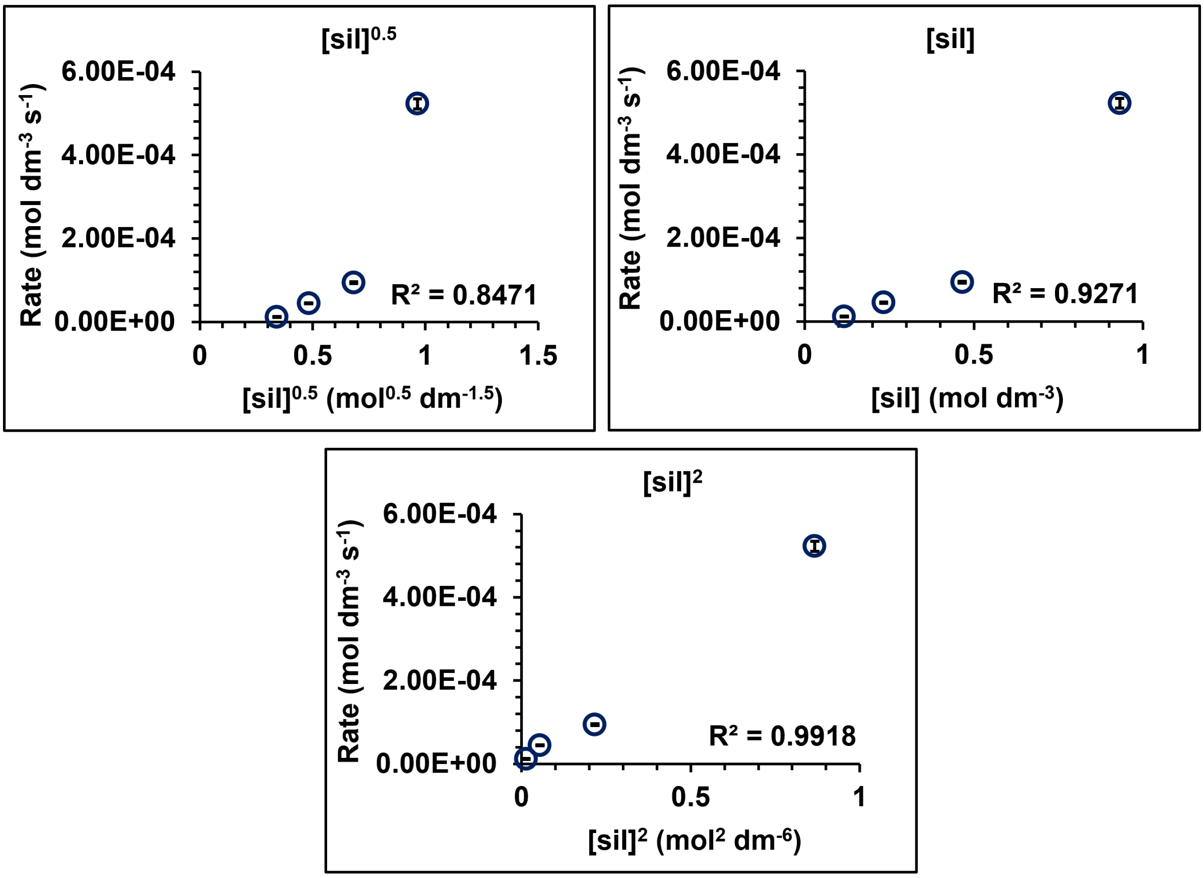

Figure S46. Rate-concentration profiles for half, first and second order in hexamethylsiloxymethylsilane using the rates for linear product formation in the hydrosilylation reaction between hexamethylsiloxymethylsilane and vinyltrimethylsilane with precatalyst **1b**. Reaction conditions: 0.0025 M **1b**, 0.11 M, 0.22 M, 0.44 M, and 0.88 M hexamethylsiloxymethylsilane, 0.88 vinyltrimethylsilane in d_2-dichloromethane, left for ~1 h thermally then irradiated 120 s with 365 nm LED reactor, 298 K).

9.2 Precatalyst 1f order in silane (post 10 s irradiation)

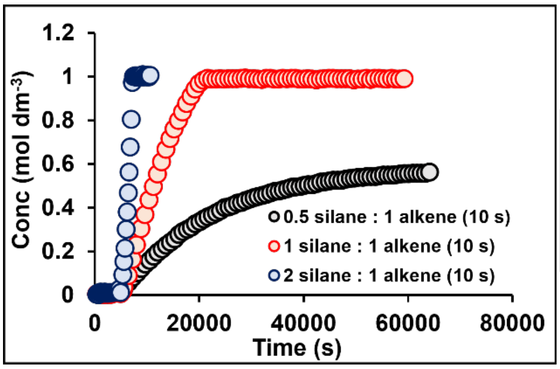

Ratio	Initial Rate (mol dm^{-3} s^{-1})
0.5 silane : 1 alkene	(2.53 ± 0.05) x 10^{-5}
1 silane : 1 alkene	(8.7 ± 0.1) x 10^{-5}
2 silane : 1 alkene	(3.6 ± 0.2) x 10^{-4}

Figure S47. Concentration-time profiles of linear product formation during the photoactivated hydrosilylation reaction between hexamethylsiloxymethylsilane and vinyltrimethylsilane using **1f** as a precatalyst. Reaction conditions: 0.0025 M **1f**, 0.5 M, 1.0 M and 2.0 M hexamethylsiloxymethylsilane, 1.0 M vinyltrimethylsilane in d_2-dichloromethane, left for ~1 h thermally then irradiated 10 s with 365 nm LED reactor, 298 K. All plots are time-shifted to show the same latency period. The table shows the initial rates following irradiation. Concentrations determined from the signal integrals from the in-situ monitored ^{1}H NMR spectra and are relative to mesitylene (integration standard).

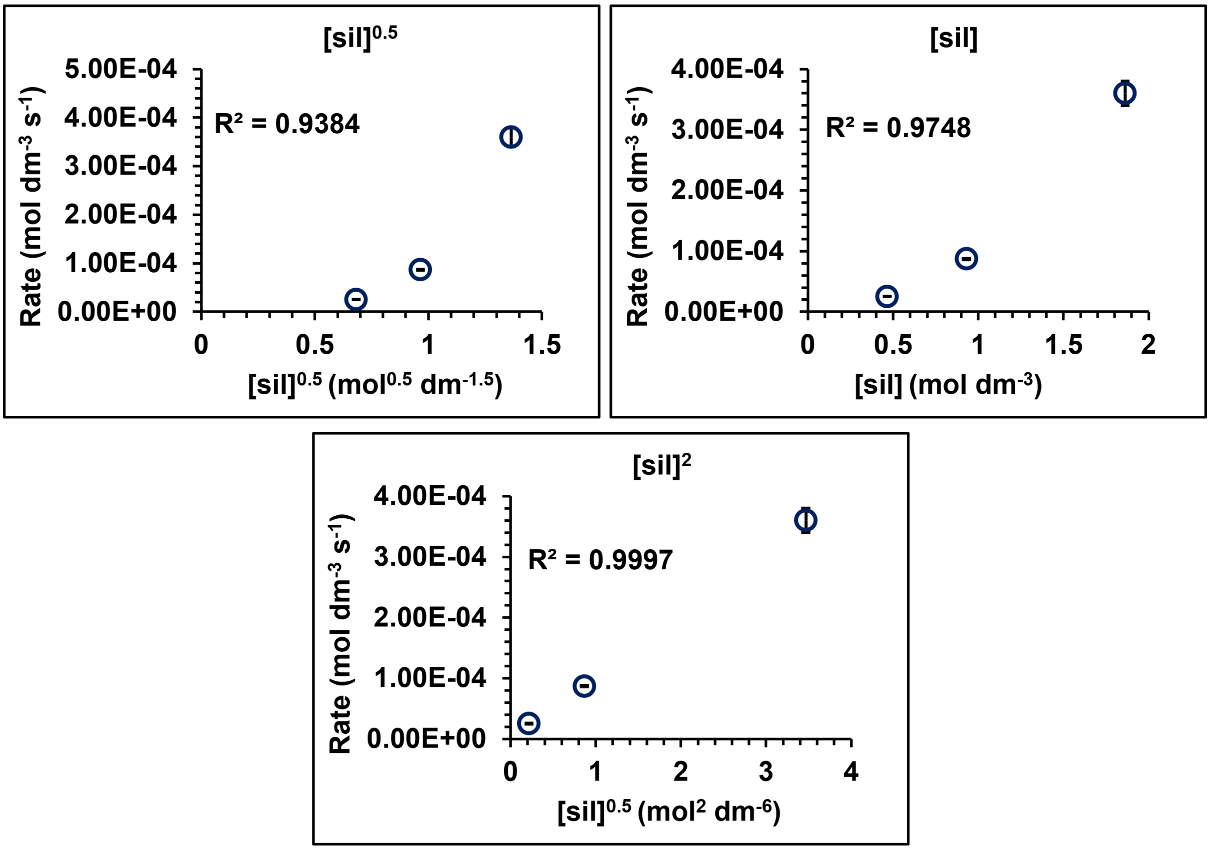

Figure S48. Rate-concentration profiles for half, first and second order in hexamethylsiloxymethylsilane using the rates for linear product formation in the hydrosilylation reaction between hexamethylsiloxymethylsilane and vinyltrimethylsilane with precatalyst **1f**. Reaction conditions: 0.0025 M **1f**, 0.5 M, 1.0 M and 2.0 M hexamethylsiloxymethylsilane, 1.0 M vinyltrimethylsilane in d_2-dichloromethane, left for ~1 h thermally then irradiated 10 s with 365 nm LED reactor, 298 K.

9.3 Precatalyst 1f order in alkene (post 10 s irradiation)

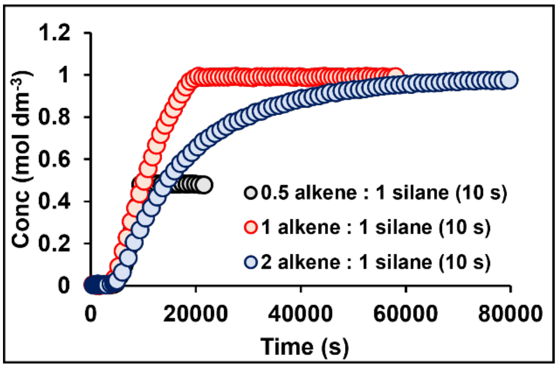

Ratio	Initial Rate (mol dm^{-3} s^{-1})
0.5 alkene : 1 silane	$(1.03 \pm 0.08) \times 10^{-4}$
1 alkene : 1 silane	$(8.7 \pm 0.1) \times 10^{-5}$
2 alkene : 1 silane	$(5.9 \pm 0.2) \times 10^{-5}$

Figure S49. Concentration-time profiles of linear product formation during the photoactivated hydrosilylation reaction between hexamethylsiloxymethylsilane and vinyltrimethylsilane using **1f** as a precatalyst. Reaction conditions: 0.0025 M **1f**, 0.5 M, 1.0 M and 2.0 M vinyltrimethylsilane, 1.0 M hexamethylsiloxymethylsilane in d_2-dichloromethane, left for ~1 h thermally then irradiated 10 s with 365 nm LED reactor, 298 K. All plots are time-shifted to show the same latency period. The table shows the initial rates following irradiation. Concentrations determined from the signal integrals from the in-situ monitored ^{1}H NMR spectra and are relative to mesitylene (integration standard).

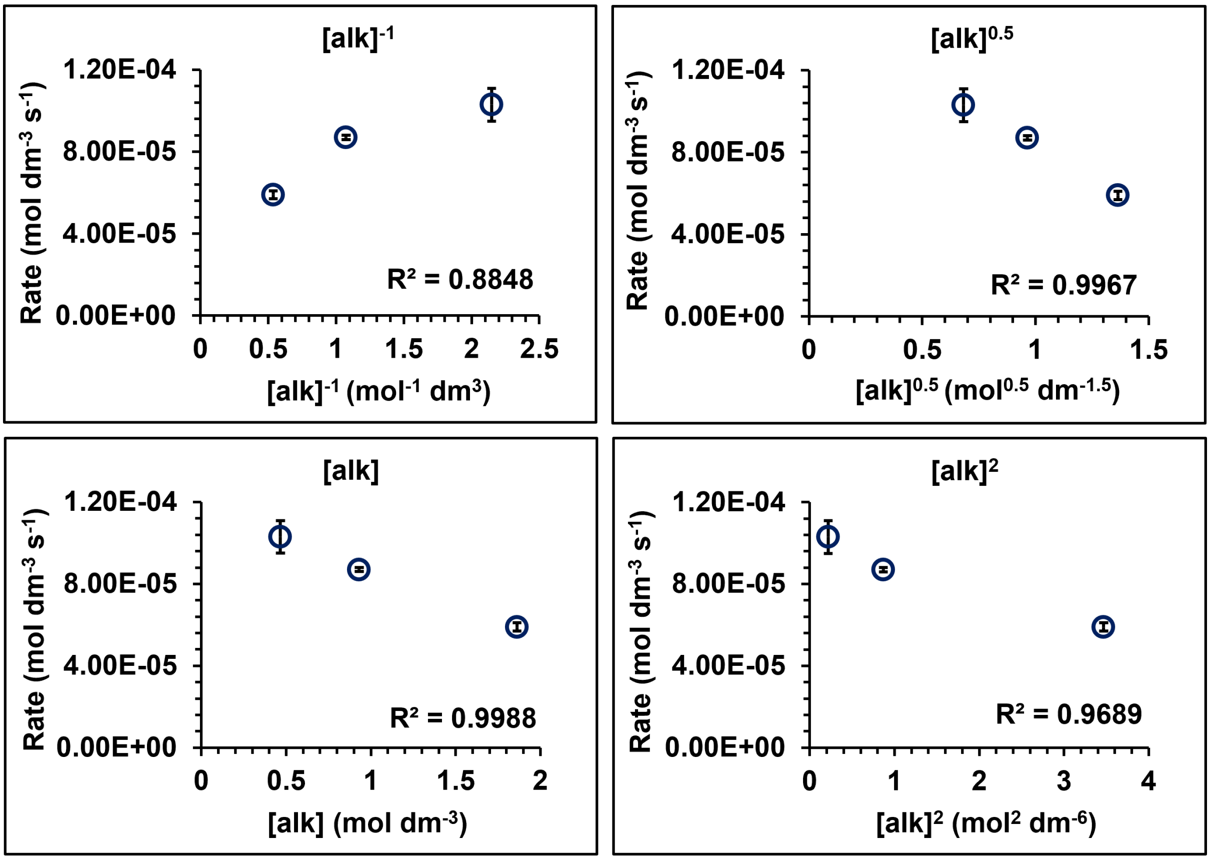

Figure S50. Rate-concentration profiles for negative first, half, first and second order in vinyltrimethylsilane using the rates for linear product formation in the hydrosilylation reaction between hexamethylsiloxymethylsilane and vinyltrimethylsilane with precatalyst **1f**. Reaction conditions 0.0025 M **1f**, 0.5 M, 1.0 M and 2.0 M vinyltrimethylsilane, 1.0 M hexamethylsiloxymethylsilane in d_2-dichloromethane, left for ~1 h thermally then irradiated 10 s with 365 nm LED reactor, 298 K).

10 Kinetic Isotope Effect

10.1 Recharges

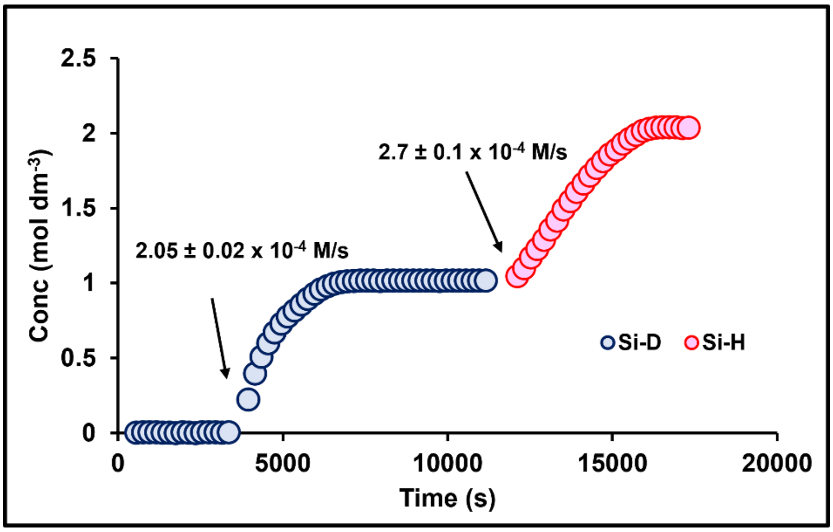

Figure S51. Concentration-time profiles of linear product formation during the photoactivated hydrosilylation reaction between d_1-hexamethylsiloxymethylsilane and vinyltrimethylsilane with a recharge of the same amount of hexamethylsiloxymethylsilane and vinyltrimethylsilane after completion. Reaction conditions: 0.0025 M **1b**, 1.0 M d_1-hexamethylsiloxymethylsilane, 1.1 M vinyltrimethylsilane in d_2-dichloromethane, left for ~1 h thermally then irradiated 120 s with 365 nm LED reactor, 298 K. Concentrations determined from the signal integrals from the in-situ monitored ^{1}H NMR spectra and are relative to mesitylene (integration standard).

11 COPASI simulations

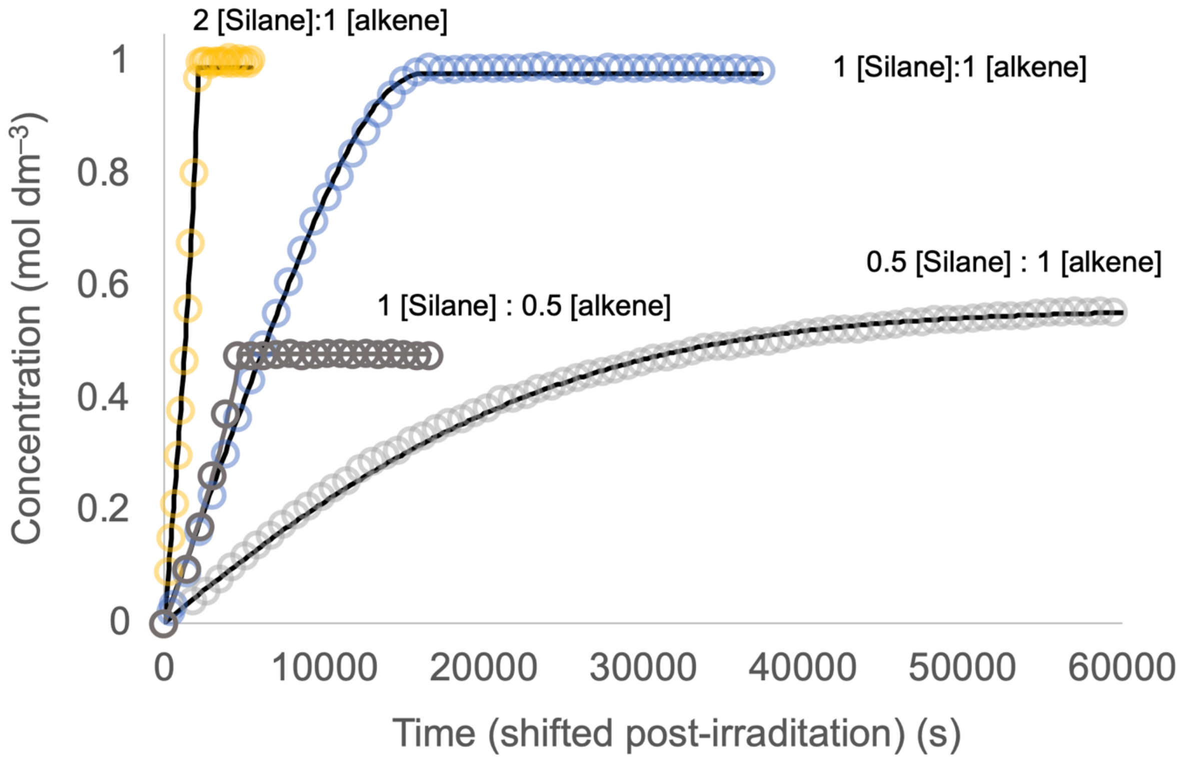

Figure S52. Concentration of linear product versus time for a variety of starting concentrations of alkene and silane substrates (~0.5 M to 2 M) and pre-catalyst **1f** (0.0025 M, 10 s irradiation time) showing the evolution of the linear product. Open circles = experimental data, solid lines = holistically simulated data derived from the catalytic manifold outlined in Scheme 5. Data is time shifted to remove the latent periods prior to irradiation.

Table S7. Relationship between $[cat]_{effective}$ used in the COPSAI model and [alkene]/[silane] concentrations.

Catalyst	[alkene]/M	[silane]/M	[alkene]x[silane]	COPASI $[cat]_{effective}$/M
	0	0	0	0
**1b**	0.81	0.84	0.6804	$6.00x10^{-6}$
	0.79	0.4	0.316	$2.40 x10^{-6}$
	0.99	1.94	1.9206	$1.50 x10^{-5}$
	0.45	0.97	0.4365	$3.30 x10^{-6}$
**1f**	0.98	0.99	0.9702	$7.00 x10^{-6}$
	0.96	0.56	0.5376	$3.50 x10^{-6}$
	0.99	2.10	2.079	$1.50 x10^{-5}$
	0.48	1.10	0.528	$3.50 x10^{-6}$

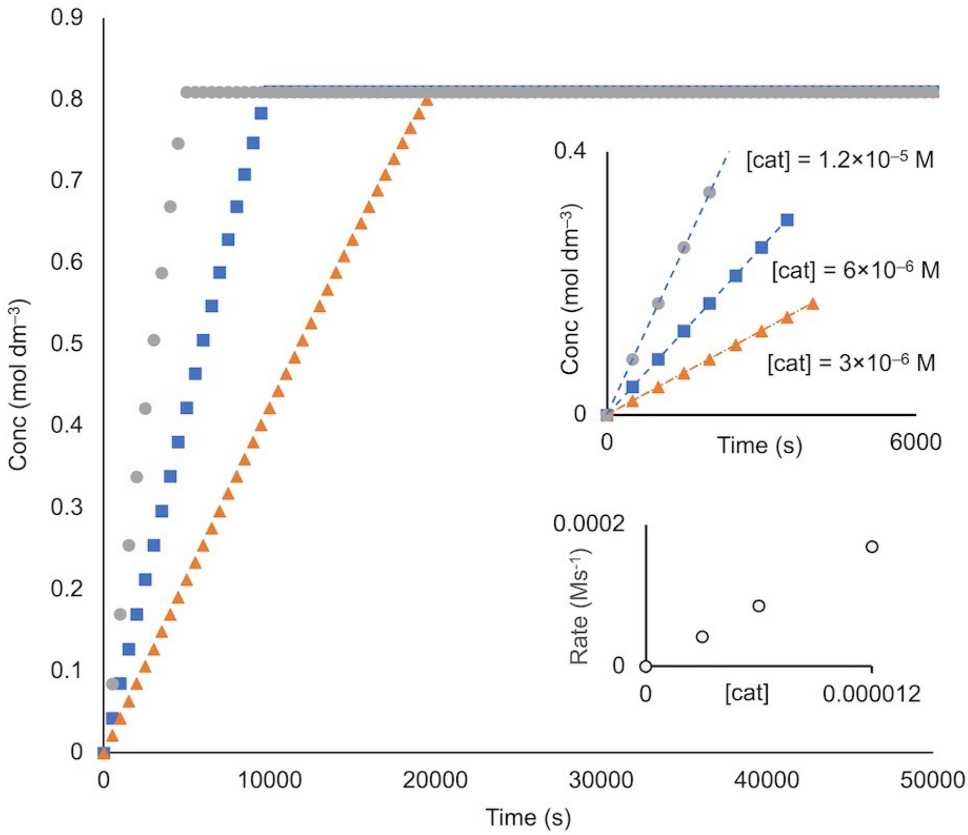

Figure S53. COPASI simulated models for the concentration of linear product, using the derived rate constants from fitting **1b** (Figure 8) for three different $[cat]_{effective}$ chosen to reflect the concentration range in the experimental data: ● = 1.2×10^{-5} M; ■ = 6.0×10^{-6} M; ▲ = 3.0×10^{-6} M; [alkene] = 0.81 M; [silane] = 0.84 M. Simulated post irradiation. Inset: (top) expansion of the first 5000 s of simulation, showing the three different starting $[cat]_{effective}$ and linear fits. Inset (bottom): relationship between simulated rates and $[cat]_{effective}$.

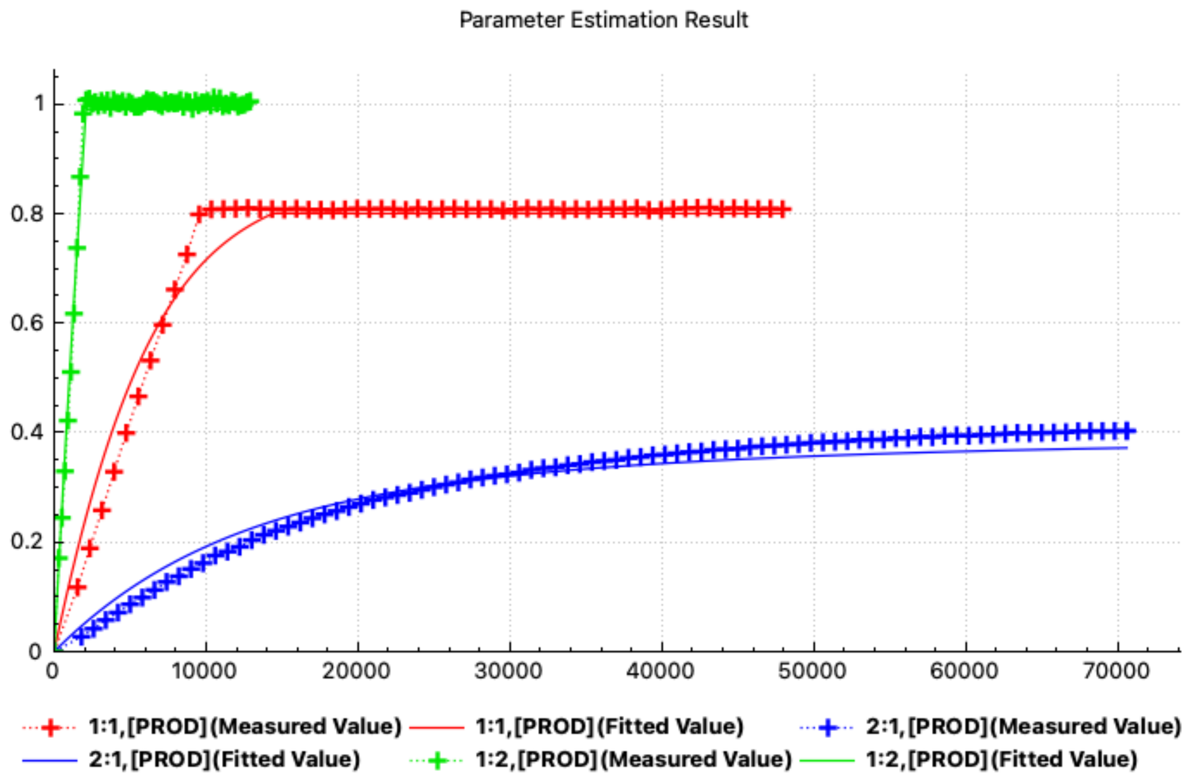

Figure S54. COPASI simulated models using the Kühn/Girolami model that requires two equivalents of silane to be involved in the catalytic cycle. [cat **1b**] = 2.5 x 10^{-6} M. Concentrations as in Figure 8.

12 Crystallographic Information

Single-crystal X-Ray diffraction data were collected on a Rigaku SuperNova diffractometer, with Cu-K_α (λ = 1.54184 Å) radiation, equipped with a nitrogen gas Oxford Cryosystems Cryostream unit[12] at the University of York. Diffraction images from raw frame data were reduced using the CrysAlisPro suite of programmes.[13] The structures were solved using SHELXT[14] and refined by full convergence on F^2 against all independent reflections by full-matrix least-squares using SHELX[15] (version 2018/3) through the Olex2 GUI.[16] All non-hydrogen atoms were refined ansiotropically and hydrogen atoms were geometrically placed and allowed to ride on their parent atoms. Distances and angles were calculated using the full covariance matrix. Crystallographic data are available free of charge *via* the Cambridge Crystallographic Data Centre, under deposition numbers 2313861-2313863.

Table S8. Crystallographic information for **1b**, **1f** and **1g**.

	**1b**	**1f**	**1g**
Chemical formula	$C_{25}H_{17}F_3N_2OPt$	$C_{26}H_{19}F_3N_2O_2Pt$	$C_{24}H_{16}F_2N_2OPt$
Formula weight	613.49	643.52	581.48
Temperature (K)	110.00(10)	110.00(10)	110.05(10)
Crystal system	Monoclinic	Monoclinic	Monoclinic
Space group	$P2_1/c$	$P2_1/c$	$P2_1/n$
a (Å)	22.76126(10)	23.8137(2)	15.4703(2)
b (Å)	5.95793(2)	6.10822(5)	7.43550(10)
c (Å)	14.84159(7)	15.27755(13)	16.5876(2)
α *(°)*	90	90	90
β *(°)*	98.2179(4)	107.8118(9)	100.7560(10)
γ *(°)*	90	90	90
V *(Å^3)*	1991.999(15)	2115.74(3)	1874.54(4)
Z	4	4	4
r_{calc} *(g cm^{-3})*	2.046	2.020	2.060
μ *(mm^{-1})*	13.615	12.893	14.343
Reflections collected	52402	19796	12228
Unique reflections	4195	4347	3581
Restraints/ parameters	0/289	0/308	0/271
R_{int}	0.0363	0.0281	0.0322
R_1 $[I > 2\sigma(I)]$	0.0179	0.0200	0.0192
wR_2 $[I > 2\sigma(I)]$	0.0466	0.0531	0.0435
GooF	1.086	1.071	1.054
Residual electron density (e Å^{-3})	0.51/ -0.94	1.21/ -0.63	0.47/ -0.68
CCDC no.	2313861	2313862	2313863

13 References

(1) Newman, C. P.; Casey-Green, K.; Clarkson, G. J.; Cave, G. W. V.; Errington, W.; Rourke, J. P. Cyclometallated platinum(ii) complexes: oxidation to, and C–H activation by, platinum(iv). *Dalton Trans.* **2007**, 3170-3182.

(2) Santoro, A.; Whitwood, A. C.; Williams, J. A. G.; Kozhevnikov, V. N.; Bruce, D. W. Synthesis, Mesomorphism, and Luminescent Properties of Calamitic 2-Phenylpyridines and Their Complexes with Platinum(II). *Chem. Mater.* **2009**, *21*, 3871-3882.

(3) Godbert, N.; Pugliese, T.; Aiello, I.; Bellusci, A.; Crispini, A.; Ghedini, M. Efficient, Ultrafast, Microwave-Assisted Syntheses of Cycloplatinated Complexes. *Eur. J. Inorg. Chem.* **2007**, *32*, 5105-5111.

(4) Altmann, P.; Cokoja, M.; Kuhn, F. E. Halide substituted Schiff-bases: Different activities in methyltrioxorhenium(VII) catalyzed epoxidation via different substitution patterns. *J. Organomet. Chem.* **2012**, *701*, 51-55.

(5) Belay, Y.; Muller, A.; Ndinteh, D. T.; Kolawole, O. A.; Adeyinka, S. A.; Fonkui, T. Y. Synthesis, antibacterial activities, cytotoxicity, and molecular docking studies of Salicyledene derivatives. *J. Mol. Struct.* **2023**, *1275*.

(6) I. Cassells, T. S., A. T. Hutton, S. Prince, G. S. Smith Impact of various lipophilic substituents on ruthenium(II), rhodium(III) and iridium(III) salicylaldimine-based complexes: synthesis, in vitro cytotoxicity studies and DNA interactions. *J. Biol. Inorg. Chem.* **2018**, *23*, 763-774.

(7) Li, S. Y.; Wang, X. B.; Kong, L. Y. Design, synthesis and biological evaluation of imine resveratrol derivatives as multi-targeted agents against Alzheimer's disease. *Eur. J. Med. Chem.* **2014**, *71*, 36-45.

(8) Parssinen, A.; Luhtanen, T.; Klinga, M.; Pakkanen, T.; Leskela, M.; Repo, T. Bis(salicylaldiminato)titanium Complexes Containing Bulky Imine Substituents: Synthesis, Characterization and Ethene Polymerization Studies. *Eur. J. Inorg. Chem.* **2005**, *2005*, 2100-2109.

(9) Acharya, S.; Maji, M.; Ruturaj, R.; Purkait, K.; Gupta, A.; Mukherjee, A. Synthesis, Structure, Stability, and Inhibition of Tubulin Polymerization by RuII–p-Cymene Complexes of Trimethoxyaniline-Based Schiff Bases. *Inorg. Chem.* **2019**, *58*, 9213-9224.

(10) Lin, S.; Pan, H.; Li, L.; Liao, R.; Yu, S.; Zhao, Q.; Sun, H.; Huang, W. AIPE-active platinum(ii) complexes with tunable photophysical properties and their application in constructing thermosensitive probes used for intracellular temperature imaging. *J. Mater. Chem. C.* **2019**, *7*, 7893.

(11) Cheng, C.; Hartwig, J. F. Mechanism of the rhodium-catalyzed silylation of arene C-H bonds. *J. Am Chem. Soc.* **2014**, *136*, 12064-12072.

(12) Cosier, J.; Glazer, A. M. A nitrogen-gas-stream cryostat for general X-ray diffraction studies. *J. Appl. Crystallogr.* **1986**, *19*, 105-107.

(13) CrysAlisPro, Oxford Diffraction/Agilent Technologies UK Ltd, Yarnton, England.

(14) Sheldrick, G. SHELXT - Integrated space-group and crystal-structure determination. *Acta Crystallogr. A.* **2015**, *71*, 3-8.

(15) Sheldrick, G. A short history of SHELX. *Acta Crystallogr. A.* **2008**, *64*, 112-122.

(16) Dolomanov, O. V.; Bourhis, L. J.; Gildea, R. J.; Howard, J. A. K.; Puschmann, H. OLEX2: a complete structure solution, refinement and analysis program. *J. Appl. Crystallogr.* **2009**, *42*, 339-341.
